# Optimisation of oral anticoagulation for stroke prevention: a scoping review of factors influencing implementation

**DOI:** 10.1136/bmjopen-2024-097847

**Published:** 2025-12-29

**Authors:** Jo Catherine Weldon, Emma P Bray, Josephine Gibson, Munirah Bangee, Brigit Chesworth, Alison Doherty, Yasemin Hirst, Deirdre Lane, Cath Harris, Aasima Saeed Patel, Caroline Watkins

**Affiliations:** 1 Stroke Research Team, School of Nursing and Midwifery, University of Lancashire, Preston, UK; 2 Applied Health Research hub, University of Lancashire, Preston, UK; 3 University of Liverpool, Liverpool, UK

**Keywords:** STROKE MEDICINE, Implementation Science, Review, PREVENTIVE MEDICINE

## Abstract

**Abstract:**

**Background:**

For people whose stroke risk would be reduced by taking a long-term oral anticoagulant (OAC), it is important to implement effective strategies to support medication initiation, adherence and persistence. To do this, a better understanding of the factors associated with implementation of interventions to optimise OAC management is needed.

**Objectives:**

This scoping review aimed to summarise the evidence-based characteristics associated with implementing interventions designed to optimise long-term OAC adherence.

**Eligibility criteria:**

Primary research (published post-2000) evaluating any intervention designed to optimise implementation of long-term OAC for stroke prevention by way of change in OAC services, staff or patient behaviour.

**Sources of evidence:**

Five databases (MEDLINE, Embase, Cumulative Index to Nursing and Allied Health Literature (CINAHL), PsycInfo, Cochrane Library) were searched from 1 January 2000 to 4 August 2023 using a combination of terms relating to population, intervention and study design.

**Charting methods:**

Titles/abstracts were screened by at least one reviewer. Data from each full text were abstracted (with 20% double-checked for accuracy) and its implementation content reviewed, guided by the Expert Recommendations for Implementing Change strategies.

**Results:**

216 studies were included, with varying descriptive reporting of implementation strategies, and only 61 (28%) self-identifying as an implementation study. The median number of implementation strategies used was three, with recently published studies (2015 onwards), those including patients receiving either direct OACs (DOACs) or vitamin K antagonists (VKAs) and those including multiple intervention targets (service, staff or patients) associated with using more implementation strategies. ‘Train and educate stakeholders’ strategies were the most commonly used, and ‘Adapt and tailor to the context’ strategies were the least used by included studies. Conversely, self-defined implementation studies were less likely to use ‘Train and educate stakeholders’ strategies, although they were positively associated with use of ‘Adapt and tailor to the context’. ‘Use evaluative & iterative’ strategies were used more frequently in studies where patients used either VKAs or DOACs, or were published more recently.

**Conclusions:**

Studies need to self-define as implementation studies, improve implementation strategy reporting and be transparently registered, alongside conducting process evaluations or more richly describing implementation processes. Future research could explore why some implementation strategies are used more than others and whether aligning strategy clusters with intervention targets results in clinically significant differences in patient care.

**STRENGTHS AND LIMITATIONS OF THIS STUDY**

- First study we are aware of in this area, to analyse data based on the Expert Recommendations for Implementing Change (ERIC) strategy clusters, making the list of ERIC strategies more accessible and understandable.
- Broad inclusion of study designs evaluating implementation of any intervention to improve oral anticoagulant uptake or adherence.
- Included research from 2000 onwards to support identification of studies applicable to current clinical practice or research methods.
- Limited to studies published in English only, due to resource constraints.
- Study authors were not contacted for missing data as this would have potentially biased the findings.

## Introduction

 Stroke is the UK’s fourth most common cause of death and main cause of severe disability.^1^ The risk of stroke from atrial fibrillation (AF), valvular heart disease or valve replacement can be substantially reduced by long-term oral anticoagulation (OAC)^2^; however, many potential OAC beneficiaries are not prescribed them,^3 4^ do not take them as instructed^5^ or prematurely discontinue them.^6^ It is imperative to implement effective methods for supporting initiation, adherence and persistence to OACs to reduce risk of death and stroke associated with non-adherence.

Until 2009, the sole options for long-term OAC treatment were vitamin K antagonists (VKAs, predominantly prescribed as warfarin). VKAs present numerous management challenges, owing to dosage variability, narrow therapeutic range and interactions with other medications and foods, which can potentially lead to avoidable death, disability and hospitalisation from strokes and serious bleeds.^7^

Since 2016, direct oral anticoagulants (DOACs; initially described from 2009 as NOACs, novel/new oral anticoagulants) have been the preferred option for the majority of AF patients given their non-inferiority to warfarin, and for some DOACs superiority, with a significant reduction in intracranial haemorrhage, with less complex dosing and monitoring^8^,^11^; however, they are not problem-free. Correct and consistent adherence and annual monitoring are still important^12^ to minimise the risk of major bleeding^13^ as well as stroke.^14^

Despite the development of these new drugs, along with tools to aid risk stratification, decision-making, choice of drug and ongoing OAC management,^8^ the initial uptake and continuation of long-term OAC in stroke prevention is still suboptimal. Improving OAC use to prevent stroke requires effective methods to be implemented to help patients and clinical staff support the initiation, adherence, persistence and long-term management of OAC.

To achieve this, a better understanding of the factors associated with implementation of interventions to optimise OAC management is needed. Therefore, this scoping review sought to (i) map published evaluations of strategies to optimise OAC for stroke prevention and (ii) examine the characteristics of published evidence associated with implementation of OAC stroke prevention interventions.

## Methods

### Design

The review is reported in accordance with the Preferred Reporting Items for Systematic Reviews and Meta-Analyses (PRISMA) Extension for Scoping Reviews (PRISMA-ScR) guidance.^15^ A PRISMA-ScR checklist is provided in online supplemental appendix 1, and the protocol can be requested from the authors.

### Patient and public involvement

There was no patient and public involvement involvement in this scoping review.

### Eligibility criteria

Primary research evaluating the effects of any intervention designed to optimise uptake and/or implementation of long-term OAC for stroke prevention via change in practitioner or patient behaviour was included. The Population, Intervention, Comparison, Outcomes, Study design (PICOS) eligibility criteria^8^ (table 1) were designed to be inclusive to capture experiences across a range of conditions and settings. Studies published in English and from the year 2000 onwards were eligible for inclusion; the latter to capture current clinical practice or research methods.

**Table 1:** PICOS criteria

Participants	Community-dwelling adults (18 years and older) on long-term oral anticoagulation for the purpose of stroke prevention and/or practitioners prescribing long-term oral anticoagulation for stroke prevention.
Intervention	Any intervention designed to optimise uptake and/or implementation of oral anticoagulation in adults at risk of stroke. Interventions which primarily target either practitioner or patient behaviours will be eligible for inclusion in the scoping review.
Comparator	Any comparator, or usual care (ie, no intervention).
Outcome	Changes in practitioner or patient behaviour and/or outcomes which reflect optimisation of oral anticoagulation management (eg, time in therapeutic range).
Study designs/setting	All study designs and settings will be eligible for inclusion in the scoping review.

### Search strategy

To identify relevant articles, five bibliographic databases (MEDLINE (Ovid), Embase (Ovid), CINAHL (EBSCOhost), PsycInfo (EBSCOhost) and Cochrane Database of Systematic Reviews (via Wiley)) were searched from 1 January 2000 to 4 August 2023 using a combination of Medical Subject Headings and keywords. The search strategy was developed by the research team in collaboration with an expert information specialist, adapted for each database (see online supplemental appendix 2). Searches were supplemented with limited retrieval of any relevant ‘sister’ publications, such as process evaluations referenced by included studies. As commonly employed in scoping reviews, we iteratively refined the eligibility criteria in response to the different types of studies retrieved in our searches; for example, studies which included patients on antithrombotic drugs and those on OACs were identified, and it was decided after discussion that such studies should be included provided that disaggregated data was available.

### Study selection

All retrieved citations were collated and deduplicated in EndNote and then transferred to Rayyan for screening. Titles and abstracts were assessed for eligibility by one reviewer (JCW, BC, EPB, AD, CH, ASP or YH), with 70% of citations progressing to full-text screening being considered by two independent reviewers, and the remaining citations were assessed by one reviewer (JCW, EPB, AD, CH, AP, YH, DL or BC). Any disagreements were resolved through discussions with a third reviewer (JCW or EPB). Decisions were discussed and agreed by the review team.

### Data abstraction

For included studies, the following data were abstracted: study information (author, year, title, country, etc); intervention details (classified into one of four types; service reorganisation, (staff) provider-focused, patient-focused or multi-category); associated Expert Recommendations for Implementing Change (ERIC) implementation strategies^16^ and author-interpreted favourability of study primary outcome. Data were abstracted using a bespoke Microsoft Excel spreadsheet, developed a priori and pilot-tested by the research team until there was good agreement. Each study was abstracted by one reviewer, with a full check performed for >20% of articles by a second independent reviewer to verify consistency and accuracy. Based on scoping review methodology, we did not perform a quality or risk of bias assessment of the included studies.^17^

### Data synthesis

**Figure 1:**
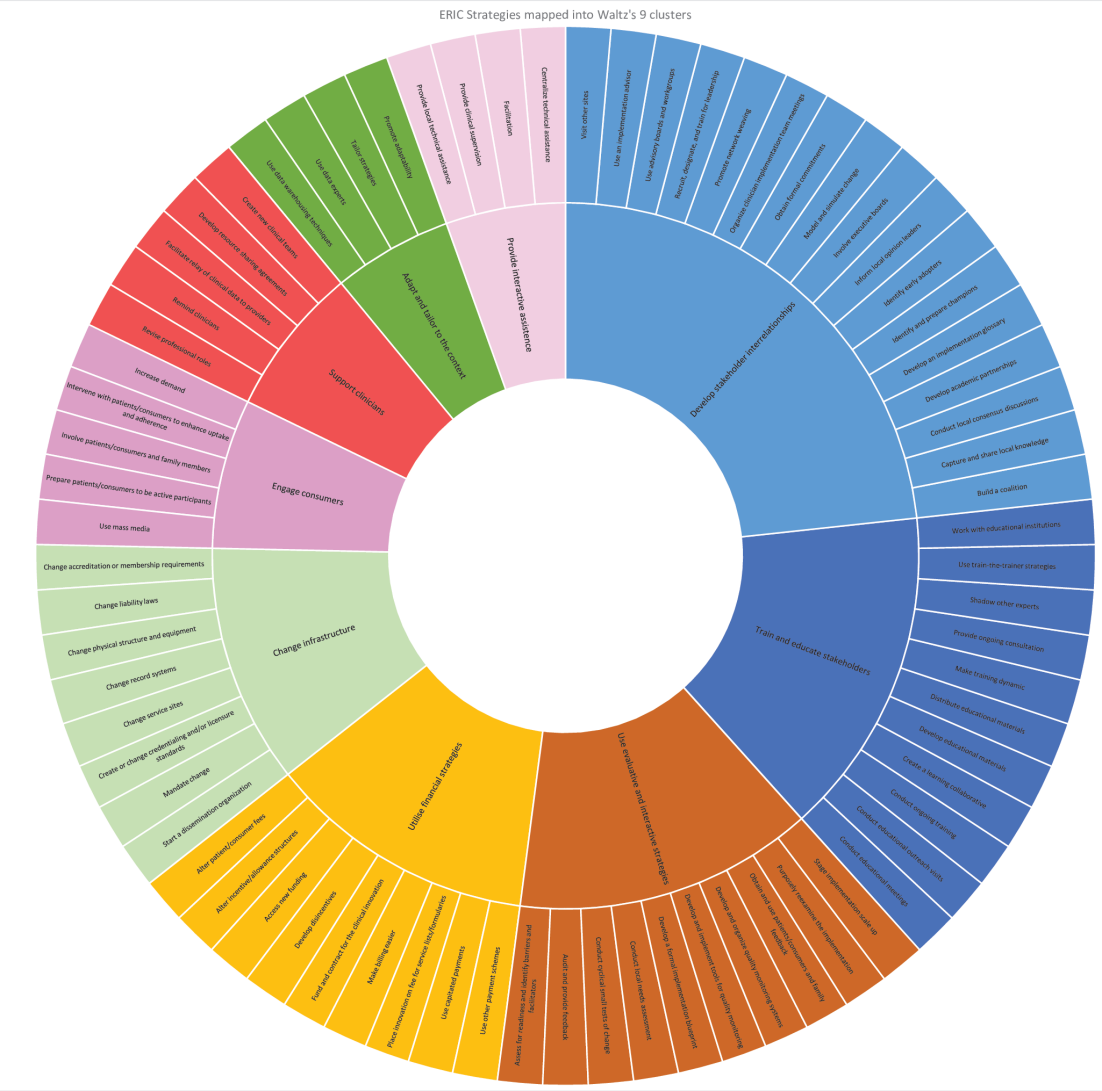
The mapping of 73 Expert Recommendations for Implementing Change (ERIC) strategies into nine clusters. Diagram stating the 73 ERIC strategies and showing how they map on to the nine clusters proposed by Waltz.^18^

Abstracted data were coded using the 73 comprehensive ERIC implementation strategies^16^ (see figure 1) which provide a clear and consistent framework for classifying how interventions are implemented into practice, removing the problem of wide and varied implementation terms and nomenclature. These were categorised into the nine clusters devised by the *ERIC compilation group* which are recommended for ‘ease of engagement and application’ of implementation strategies^18^ (figure 1). The definitions of each ERIC strategy can be found in online supplemental appendix 3.

Study characteristics were described using numbers and percentages (%). To determine whether using a greater number of ERIC strategies was associated with specific study characteristics, the median number of strategies (median=3; 3 or less=0; 4 or more=1) used was chosen to dichotomise the data, due to the skewed distribution of data, and to ease understanding of the differences between study characteristics.

Unadjusted and adjusted logistic regression models were conducted to investigate associations between study characteristics and the use of ≥4 ERIC strategies with results expressed as odds ratios or adjusted odds ratios, with corresponding 95% CIs, and p-values less than ≤0.05 to establish statistical significance.

## Results

**Figure 2:**
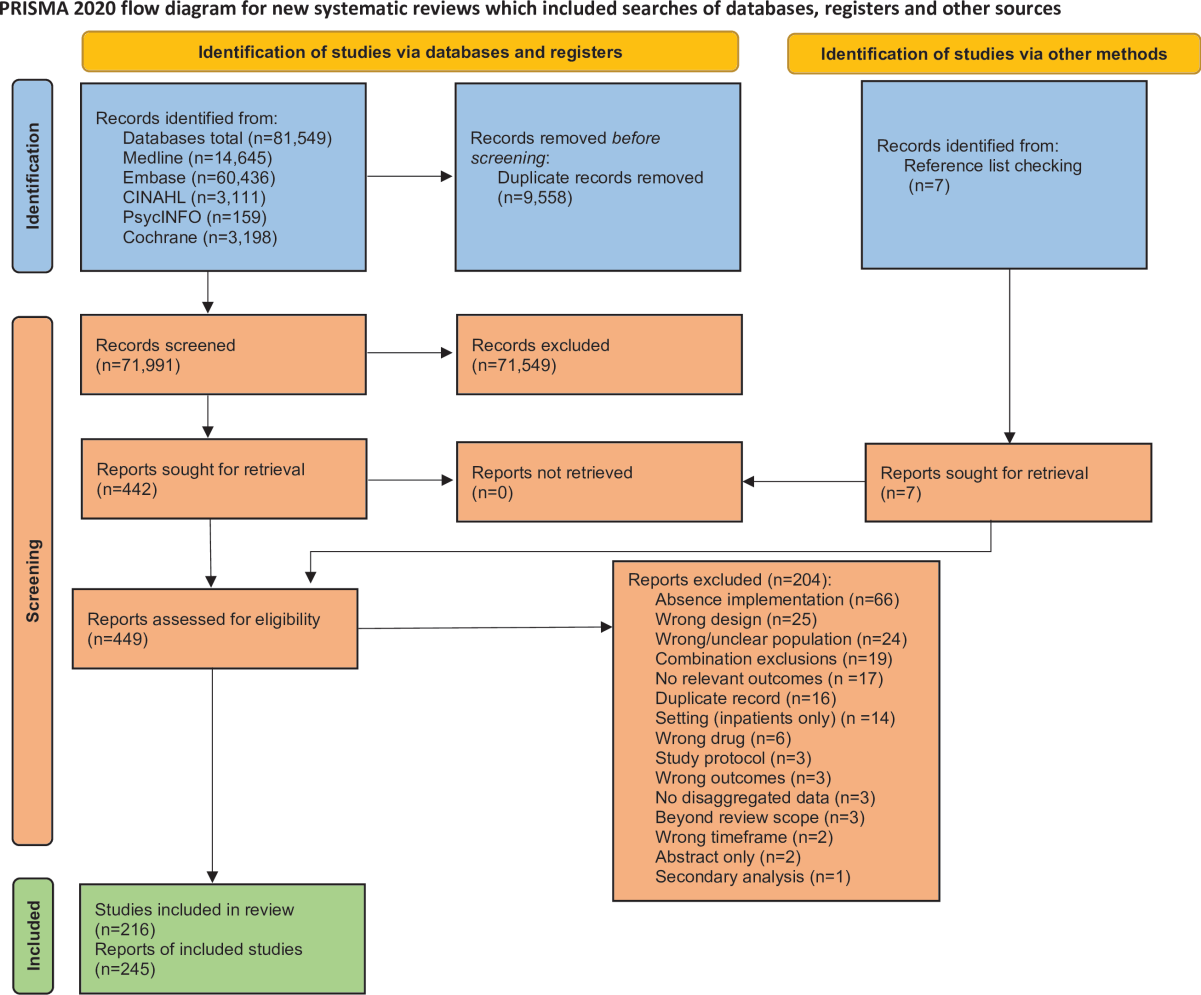
Preferred Reporting Items for Systematic Reviews and Meta-Analyses (PRISMA) flow diagram. Study selection process following PRISMA 2020 guidelines showing the flow of information through different phases of the scoping review. Numbers of studies are provided for identification through database searching, screening of titles/abstracts, full-text assessment for eligibility and final inclusion. Reasons for exclusion at full-text review are categorised and quantified.

The flow of citations through the study is shown in figure 2. Searches identified 81 549 citations, with a further seven texts retrieved through reference list checks. After the removal of duplicates, 71 998 citations were screened on title and abstract. Of these, 449 (0.6%) articles progressed to full-text assessment; 245 reports (216 individual studies) met the inclusion criteria. A full list of the 245 references assessed for included studies, and the 204 excluded citations, can be found in online supplemental appendix 4.

### Characteristics of included studies

More than half (57%) were published since 2015 (n=124), with most originating (57%, n=123) from Anglophone countries, North America (37%, n=80) and Europe (34%, n=75) (table 2). Forty-one percent (n=88) were randomised controlled trials (RCTs) (or variant of), and 28% (n=61) were on a clinical trials or similar register. Most studies (62%, n=134) were conducted solely in secondary care, and 24% (n=52) were conducted exclusively in primary care. Almost two-thirds of available evidence concentrated solely on VKA treatments (n=140, 65%), and over three-quarters focused on patient populations with AF (AF+other=48%, n=104; AF only: 29%, n=62).

**Table 2:** Descriptive characteristics of the included studies

		Total N (%)	3 or fewer ERIC strategies used	4 or more ERIC strategies used
			N papers (%)	N papers (%)
ERIC strategies		216 (100%)	123 (56.9%)	93 (43.1%)
Study outcomes	Favourable	156 (72.2%)	82 (66.7%)	74 (79.6%)
Identifies as an implementation study	Yes	61 (28.2%)	27 (22%)	34 (36.6%)
Year of publication	2000–2004	22 (10.2%)	19 (15.4%)	3 (3.2%)
	2005–2009	29 (13.4%)	19 (15.4%)	10 (10.8%)
	2010–2014	41 (19%)	30 (24.4%)	11 (11.8%)
	2015–2019	58 (26.9%)	35 (28.5%)	23 (24.7%)
	2020–2023	66 (30.6%)	20 (16.3%)	46 (49.5%)
Anglophone country	Yes	123 (56.9%)	67 (54.5%)	56 (60.2%)
Study location	Africa	3 (1.4%)	2 (1.6%)	1 (1.1%)
	Asia	33 (15.3%)	17 (13.8%)	16 (17.2%)
	Australasia	18 (8.3%)	8 (6.5%)	10 (10.8%)
	North America	80 (37%)	43 (35%)	37 (39.8%)
	South America	5 (2.3%)	4 (3.3%)	1 (1.1%)
	Rest of Europe	56 (25.6%)	36 (29.3%)	20 (21.5%)
	United Kingdom	19 (8.8%)	12 (9.8%)	7 (7.5%)
	Multi-country	2 (0.9%)	1 (0.8%)	1 (1.1%)
Type of OAC	VKAs and DOACs	40 (18.5%)	13 (10.6%)	27 (29%)
	DOACs only	22 (10.6%)	11 (8.9%)	12 (12.9%)
	Not specified	13 (6%)	9 (7.3%)	4 (4.3%)
Population	AF and another indication for OAC	104 (48.1%)	56 (45.5%)	48 (51.6%)
	AF only	62 (28.7%)	40 (32.5%)	22 (23.7%)
	NVAF	10 (4.6%)	2 (1.6%)	8 (8.6%)
	Other indications	18 (8.3%)	13 (10.6%)	5 (5.4%)
	Population indication unclear	22 (10.2%)	12 (9.8%)	12 (9.8%)
Setting	Primary and secondary care	30 (13.9%)	17 (13.8%)	13 (14%)
	Secondary care	134 (62%)	81 (60.4%)	53 (39.6%)
	Primary care	52 (24.1%)	25 (48.1%)	27 (51.9%)
Study design	Observational	47 (21.8%)	21 (17.1%)	26 (28%)
	Quasi-experimental	81 (37.5%)	42 (34.1%)	39 (41.9%)
	RCT or variant-RCT	88 (40.7%)	60 (48.8%)	28 (30.1%)
	VKAs only (warfarin)	140 (64.8%)	90 (73.2%)	50 (53.8%)
Registered	Yes	61 (28.2%)	41 (33.3%)	20 (21.5%)
Number of interventions tested *M* (SD)	Range (1–6)	1.44 (.91)	1.32 (.74)	1.59 (1.09)
Intervention type	Service reorganisation	45 (20.8%)	25 (20.3%)	20 (21.5%)
	Provider-focused (staff)	68 (31.5%)	42 (34.1%)	26 (28%)
	Patient-focused	88 (40.7%)	53 (43.1%)	35 (37.6%)
	Multicategory	15 (6.9%)	3 (2.4%)	12 (12.9%)
Reasons for primary outcome reported	Yes	131 (60.6%)	64 (52.0%)	67 (72.0%)

AF, atrial fibrillation; DOACs, direct oral anticoagulants; ERIC, Expert Recommendations for Implementing Change; M, mean; NVAF, non-valvular atrial fibrillation; OAC, oral anticoagulation; RCT, randomised controlled trial; SD, standard deviation; VKA, vitamin K antagonists.

Included studies tested between one to six interventions, with a mean of 1.44 (SD 0.91). Tested interventions were most commonly patient-focused (41%, n=88). Most (72%, n=156) were reported by study authors as attaining wholly favourable primary outcomes; of these, two-thirds (61%, n=131) suggested possible reasons for their study’s findings.

### Analysis of implementation strategies

Only 61 studies (28%) explicitly self-identified as a quality improvement (QI) and/or implementation study. Additionally, the descriptive reporting of the strategies to support implementation varied, with some providing a detailed account of their implementation strategies, while others gave only very limited details (see online supplemental appendix 5 for examples).

### Factors associated with using four or more Expert Recommendations for Implementing Change strategies

Adjusted regression analyses indicated that studies using ≥4 ERIC strategies were associated with three characteristics: more recently published (since 2015), including patients taking either VKA or DOAC, compared with single treatment populations and using a combination of intervention targets (multicategory), compared with singularly focused interventions (service reorganisation; staff-focused; patient-focused) (table 3).

**Table 3:** Factors associated with using four or more ERIC strategies

	Unadjusted model		Adjusted model	
	OR	95% CI	aOR	95% CI
Study outcomes				
Not favourable/partially favourable	Ref		Ref	
Favourable	1.95	1.04 to 3.65*	2.01	0.89 to 4.55
Identifies as an implementation study				
No	Ref		Ref	
Yes	2.05	1.12 to 3.74*	1.17	0.54 to 2.55
Year of publication				
2000–2004	Ref		Ref	
2005–2009	3.33	0.79 to 14.05	5.00	0.93 to 26.81
2010–2014	2.32	0.57 to 9.42	4.11	0.77 to 21.97
2015–2019	4.16	1.11 to 15.68	5.94	1.11 to 31.70*
2020–2023	14.57	3.87 to 54.86†	20.98	3.69 to 119.32†
Study design				
Observational	Ref		Ref	
Quasi-experimental	0.75	0.36 to 1.54	1.24	0.48 to 3.24
RCT or variant-RCT	0.38	0.18 to 0.78*	0.62	0.23 to 1.67
Type of OAC				
VKAs only (warfarin)	Ref		Ref	
VKAs and DOACs	3.74	1.77 to 7.89†	4.77	1.56 to 14.57*
DOACs only	1.96	0.81 to 4.77	1.50	0.44 to 5.16
Not specified	0.80	0.23 to 2.73	0.68	0.13 to 3.60
Population				
AF and another indication for OAC	Ref		Ref	
AF only	0.64	0.34 to 1.23	0.41	0.16 to 1.06
NVAF	4.67	0.95 to 23.04	1.44	0.21 to 9.71
Other indications	0.45	0.15 to 1.35	0.61	0.15 to 2.41
Population indication unclear	0.97	0.38 to 2.45	1.21	0.39 to 3.72
Setting				
Both settings	Ref		Ref	
Secondary care	0.86	0.38 to 1.91	0.73	0.28 to 1.92
Primary care	1.41	0.57 to 3.49	1.24	0.39 to 3.72
Number of interventions tested M (SD)				
Range (1–6)1.43 (0.91)	1.39	1.02 to 1.89*	1.16	0.75 to 1.79
Intervention type				
Service reorganisation	Ref		Ref	
Provider-focused	0.77	0.36 to 1.66	0.59	0.21 to 1.62
Patient-focused	0.83	0.39 to 1.71	1.01	0.39 to 2.61
Multicategory	5.00	1.24 to 20.18*	13.90	2.02 to 95.69*

* p≤0.05.

† p≤0.001.

aOR, adjusted odds ratio; DOACs, direct oral anticoagulants; ERIC, Expert Recommendations for Implementing Change; M, mean; NVAF, non-valvular atrial fibrillation; OR, odds ratio; RCT, randomised controlled trial; VKAs, vitamin K antagonists.

### Factors associated with the use of implementation strategies based on Expert Recommendations for Implementing Change strategy clusters

**Figure 3:**
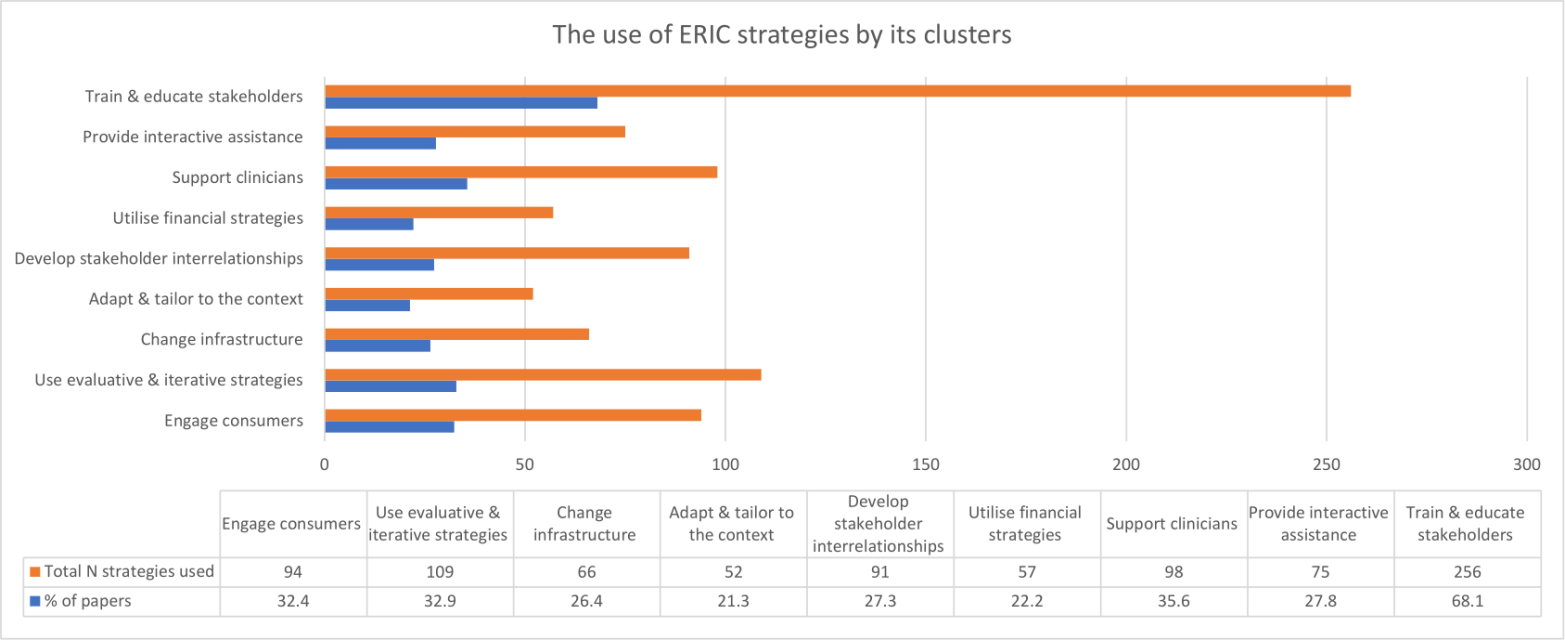
The use of Expert Recommendations for Implementing Change (ERIC) strategies by its clusters. 898 ERIC strategies were found in the included papers. A bar graph and table visualise the distribution of those ERIC strategies across the nine clusters, along with the percentage of papers containing strategies related to each cluster.

The most commonly used ERIC cluster was ‘Train and educate stakeholders’ (68%, n=256), while the least applied were ‘Adapt and tailor to the context’ (21%, n=52) and ‘Utilise financial strategies’ (22%, n=57) (figure 3).

‘Engage consumer’ strategies were positively associated, and ‘Change infrastructure’ strategies negatively associated, with patient-focused rather than service-focused interventions, whereas ‘Developing stakeholder interrelationships’ strategies were less likely to be associated with staff-focused than patient-focused studies. ‘Developing stakeholder interrelationships’ strategies were also more likely to be seen in studies evaluating a higher number of interventions (online supplemental appendix 6).

‘Use evaluative & iterative’ strategies were used more frequently in studies where the population used either VKAs or DOACs, or were published more recently; however, these strategies were used less often in studies including only AF patients. More recent studies, and those reporting a favourable primary outcome, were associated with the use of ‘Provide interactive assistance’ strategies.

‘Adapt & tailor to the context’ strategies were positively associated with reports that identified as implementation studies, but negatively associated with having a quasi-experimental design over observational designs. Studies that used ‘Train & educate stakeholders’ strategies were less likely to be used in self-defined implementation studies and studies conducted solely in secondary care.

Studies using ‘Utilise financial strategies were less likely to be conducted solely in primary care, and those employing ‘Support clinicians’ strategies were less likely to be an RCT rather than an observational study.

## Discussion

This scoping review explored the characteristics and implementation strategies of 216 studies that tested interventions designed to optimise oral anticoagulation for stroke prevention. A wide range of implementation strategies was used, with ‘Train and educate stakeholders’ dominating, followed by those related to ‘Support clinicians’ and ‘Engage consumers’. Fewer studies focused on ‘Adapt and tailor to the context’, ‘Utilise financial strategies’ or ‘Change infrastructure’, which highlights an emphasis on staff and patients over wider environmental or resource factors when improving uptake and adherence to OACs.

Although VKA prescription has declined, and DOACs increased, since 2016,^19 20^ a seismic shift in OAC prescribing practice occurred during the COVID-19 pandemic,^21^,^23^ when DOACs became the recommended first-line treatment option, and patients on warfarin were switched to a DOAC where suitable. The included studies do not reflect this clinical reality; although 60% of the included studies were published since 2015, two-thirds of them only recruited patients prescribed a VKA. Many studies included in this review would have been conceived and designed pre-pandemic, and as such, the sample likely reflects research-practice lag. The results also possibly reflect beliefs that DOAC management is less problematic; some studies counter this assumption^6 14^ identifying suboptimal uptake, persistence and adherence with this class of OAC.

This review identified that 43% of studies incorporated ≥4 ERIC strategies, with these associated with being conducted more recently, having a patient mix on VKAs or DOACs and using a combination of intervention targets (service, staff, patient). These positive indications may suggest a growing academic and practical understanding of, and engagement with, implementation science in recent years. However, as reported in similar research,^24^,^26^ only just over a quarter of included studies explicitly identified as QI or implementation studies, with implementation strategies often absent or minimally reported, and implementation theory, models or frameworks not systematically incorporated into study design. This could hinder healthcare staff readily identifying relevant evidence and may mean implementation efforts are not optimally designed or operationalised. However, it was promising that identifiable implementation studies were associated with ‘Adapt & tailor to the context’ strategies, as adaptation consideration is central to implementation efforts.

Almost three-quarters of included studies reported wholly favourable primary outcomes. Having a favourable outcome and being more recently published (since 2010) were both associated with studies using ‘Provide interactive assistance’ implementation strategies, which is encouraging as embedding technological support within interventions has recently increased significantly, aiding more consistent adherence.

To our knowledge, this is the first scoping review to explicitly use the ERIC implementation strategy clusters^18^ to describe evidence for implementing OAC optimisation interventions across settings and patient populations. Using nine coherently overarching clusters, rather than 73 discrete and unstructured strategies, aided the analysis and interpretation. However, this review was limited to studies published in English due to resource constraints and covers the literature from 2000 onwards only. We did not contact authors for missing data to mitigate within-review bias.

### Implications for future research

Further research could explore why some implementation strategies are used more than others, especially to understand why system- and policy-level strategies are used the least. Future studies could also explore whether aligning strategy clusters with intervention targets results in clinically significant differences in patient care, for example, does using ‘Train & educate stakeholders’ strategies for staff-focused interventions and ‘Engage consumers’ strategies for patient-focused interventions result in improved uptake and adherence to OACs?

Studies could explicitly consider appropriate implementation or QI approaches via process evaluation and describe implementation components in detail in their reporting. Improved implementation strategy reporting (eg, Standards for Reporting Implementation studies^27^ or Standards for Quality Improvement Reporting Excellence^28^) would also enable clinicians and policymakers to identify salient components of effective interventions and consider how they might be translated into routine practice, in addition to appropriate use of ERIC strategies^16^ for aiding design of comprehensive implementation. Authors were likely unaware they were using implementation strategies, rather than neglecting to describe them.^25^ Therefore, exploring why studies fail to self-identify as implementation research could be fruitful to improve researchers’ knowledge and skills in this field and increase the visibility of implementation science.

## Conclusions

This review mapped characteristics and implementation strategies reported in the available evidence base and identified an absence of self-defined implementation studies and transparently registered research. Conducting process evaluations and more richly describing implementation processes would support the evidence base, as would exploring whether using ‘Train and educate stakeholders’ strategies results in clinically important differences for staff-focused interventions and whether ‘Engaging consumers’ strategies do likewise for patient-focused interventions.

## Supplementary material

10.1136/bmjopen-2024-097847 online supplemental file 1

## Data Availability

Data are available upon reasonable request.

## References

[1] Patel A, Berdunov V, King D (2017). Current, future and avoidable costs of stroke in the UK. Executive summary Part 2: Societal costs of stroke in the next 20 years and potential returns from increased spending on research. Centre for Primary Care & Public Health, Queen Mary University of London and the Personal Social Services Research Unit. London School of Economics and Political Science for the Stroke Association.

[2] Mant J, Hobbs FR, Fletcher K (2007). Warfarin versus aspirin for stroke prevention in an elderly community population with atrial fibrillation (the Birmingham Atrial Fibrillation Treatment of the Aged Study, BAFTA): a randomised controlled trial. The Lancet.

[3] Shantsila E, Wolff A, Lip GYH (2015). Optimising stroke prevention in patients with atrial fibrillation: application of the GRASP-AF audit tool in a UK general practice cohort. Br J Gen Pract.

[4] Turner GM, Calvert M, Feltham MG (2016). Under-prescribing of Prevention Drugs and Primary Prevention of Stroke and Transient Ischaemic Attack in UK General Practice: A Retrospective Analysis. PLoS Med.

[5] Salmasi S, Loewen PS, Tandun R (2020). Adherence to oral anticoagulants among patients with atrial fibrillation: a systematic review and meta-analysis of observational studies. BMJ Open.

[6] Banerjee A, Benedetto V, Gichuru P (2020). Adherence and persistence to direct oral anticoagulants in atrial fibrillation: a population-based study. Heart.

[7] Wan Y, Heneghan C, Perera R (2008). Anticoagulation control and prediction of adverse events in patients with atrial fibrillation: a systematic review. Circ Cardiovasc Qual Outcomes.

[8] National Institute for Health and Care Excellence (NICE) (2021). NICE guideline [NG196] Atrial fibrillation: diagnosis and management; Published: 27 April 2021 Last updated: 30 June 2021.

[9] National Institute for Health and Care Excellence (NICE) (2021). NICE guideline [NG208] Heart valve disease presenting in adults: investigation and management; Published: 17 November 2021.

[10] Gelder IC, Rienstra M, Bunting KV (2024). 2024 ESC Guidelines for the management of atrial fibrillation developed in collaboration with the European Association for Cardio-Thoracic Surgery (EACTS): Developed by the task force for the management of atrial fibrillation of the European Society of Cardiology (ESC), with the special contribution of the European Heart Rhythm Association (EHRA) of the ESC. Endorsed by the European Stroke Organisation (ESO). Eur Heart J.

[11] Vahanian A, Beyersdorf F, Praz F (2022). Corrigendum to: 2021 ESC/EACTS Guidelines for the management of valvular heart disease: Developed by the Task Force for the management of valvular heart disease of the European Society of Cardiology (ESC) and the European Association for Cardio-Thoracic Surgery (EACTS). Eur Heart J.

[12] Rodriguez RA, Carrier M, Wells PS (2013). Non-adherence to new oral anticoagulants: a reason for concern during long-term anticoagulation?. J Thromb Haemost.

[13] Inohara T, Holmes DN, Pieper K (2020). Decline in renal function and oral anticoagulation dose reduction among patients with atrial fibrillation. Heart.

[14] Maura G, Pariente A, Alla F (2017). Adherence with direct oral anticoagulants in nonvalvular atrial fibrillation new users and associated factors: a French nationwide cohort study. Pharmacoepidemiol Drug Saf.

[15] Tricco AC, Lillie E, Zarin W (2018). PRISMA Extension for Scoping Reviews (PRISMA-ScR): Checklist and Explanation. Ann Intern Med.

[16] Powell BJ, Waltz TJ, Chinman MJ (2015). A refined compilation of implementation strategies: results from the Expert Recommendations for Implementing Change (ERIC) project. Implement Sci.

[17] Munn Z, Peters MDJ, Stern C (2018). Systematic review or scoping review? Guidance for authors when choosing between a systematic or scoping review approach. BMC Med Res Methodol.

[18] Waltz TJ, Powell BJ, Matthieu MM (2015). Use of concept mapping to characterize relationships among implementation strategies and assess their feasibility and importance: results from the Expert Recommendations for Implementing Change (ERIC) study. Implement Sci.

[19] Afzal S, Zaidi STR, Merchant HA (2021). Prescribing trends of oral anticoagulants in England over the last decade: a focus on new and old drugs and adverse events reporting. J Thromb Thrombolysis.

[20] Alkhameys S, Barrett R (2022). Impact of the COVID-19 pandemic on England’s national prescriptions of oral vitamin K antagonist (VKA) and direct-acting oral anticoagulants (DOACs): an interrupted time series analysis (January 2019–February 2021). Curr Med Res Opin.

[21] NHS England (2023). Community pharmacy oral anticoagulant safety audit 2021/22. https://www.england.nhs.uk/long-read/community-pharmacy-oral-anticoagulant-safety-audit-2021-22/#:~:text=The%20most%20commonly%20prescribed%20DOACs,with%2027%2C276%20patients%20(20.8%25).

[22] Curtis HJ, MacKenna B, Walker AJ (2021). OpenSAFELY: impact of national guidance on switching anticoagulant therapy during COVID-19 pandemic. Open Heart.

[23] NHS England Clinical guide for the management of anticoagulant services during the coronavirus pandemic.

[24] Lovero KL, Kemp CG, Wagenaar BH (2023). Application of the Expert Recommendations for Implementing Change (ERIC) compilation of strategies to health intervention implementation in low- and middle-income countries: a systematic review. Implement Sci.

[25] Chen JI, Roth B, Dobscha SK (2024). Implementation strategies in suicide prevention: a scoping review. Implement Sci.

[26] Murrell JE, Pisegna JL, Juckett LA (2021). Implementation strategies and outcomes for occupational therapy in adult stroke rehabilitation: a scoping review. Implement Sci.

[27] Pinnock H, Barwick M, Carpenter CR (2017). Standards for Reporting Implementation Studies (StaRI) Statement. BMJ.

[28] Ogrinc G, Mooney SE, Estrada C (2008). The SQUIRE (Standards for QUality Improvement Reporting Excellence) guidelines for quality improvement reporting: explanation and elaboration. *Quality and Safety in Health Care*.

