# Supplementary material for: Optimisation of oral anticoagulation for stroke prevention: a scoping review of factors influencing implementation
Source: BMJ Open. 2025 Dec 29;15(12):e097847. doi: 10.1136/bmjopen-2024-097847 (PMC12750798; doi:10.1136/bmjopen-2024-097847)
Supplement: online supplemental file 1 [file bmjopen-15-12-s001.docx]

**Appendices**

# Appendix 1. Preferred Reporting Items for Systematic reviews and Meta-Analyses extension for Scoping Reviews (PRISMA-ScR) Checklist

| **SECTION** | **ITEM** | **PRISMA-ScR CHECKLIST ITEM** | **REPORTED ON PAGE #** |
| --- | --- | --- | --- |
| **TITLE** | | | |
| Title | 1 | Identify the report as a scoping review. | 1 |
| **ABSTRACT** | | | |
| Structured summary | 2 | Provide a structured summary that includes (as applicable): background, objectives, eligibility criteria, sources of evidence, charting methods, results, and conclusions that relate to the review questions and objectives. | 2-3 |
| **INTRODUCTION** | | | |
| Rationale | 3 | Describe the rationale for the review in the context of what is already known. Explain why the review questions/objectives lend themselves to a scoping review approach. | 5-6 |
| Objectives | 4 | Provide an explicit statement of the questions and objectives being addressed with reference to their key elements (e.g., population or participants, concepts, and context) or other relevant key elements used to conceptualize the review questions and/or objectives. | 7 |
| **METHODS** | | | |
| Protocol and registration | 5 | Indicate whether a review protocol exists; state if and where it can be accessed (e.g., a Web address); and if available, provide registration information, including the registration number. | 6 |
| Eligibility criteria | 6 | Specify characteristics of the sources of evidence used as eligibility criteria (e.g., years considered, language, and publication status), and provide a rationale. | 7 |
| Information sources* | 7 | Describe all information sources in the search (e.g., databases with dates of coverage and contact with authors to identify additional sources), as well as the date the most recent search was executed. | 7 |
| Search | 8 | Present the full electronic search strategy for at least 1 database, including any limits used, such that it could be repeated. | Appendix 2 |
| Selection of sources of evidence† | 9 | State the process for selecting sources of evidence (i.e., screening and eligibility) included in the scoping review. | 7-8 |
| Data charting process‡ | 10 | Describe the methods of charting data from the included sources of evidence (e.g., calibrated forms or forms that have been tested by the team before their use, and whether data charting was done independently or in duplicate) and any processes for obtaining and confirming data from investigators. | 8 |
| Data items | 11 | List and define all variables for which data were sought and any assumptions and simplifications made. | 8-9 |
| Critical appraisal of individual sources of evidence§ | 12 | If done, provide a rationale for conducting a critical appraisal of included sources of evidence; describe the methods used and how this information was used in any data synthesis (if appropriate). | 8 |
| Synthesis of results | 13 | Describe the methods of handling and summarizing the data that were charted. | 8-9 |
| **RESULTS** | | | |
| Selection of sources of evidence | 14 | Give numbers of sources of evidence screened, assessed for eligibility, and included in the review, with reasons for exclusions at each stage, ideally using a flow diagram. | Figure 2 |
| Characteristics of sources of evidence | 15 | For each source of evidence, present characteristics for which data were charted and provide the citations. | 9-10;  Appendix 4: References to included and excluded studies |
| Critical appraisal within sources of evidence | 16 | If done, present data on critical appraisal of included sources of evidence (see item 12). | n/a |
| Results of individual sources of evidence | 17 | For each included source of evidence, present the relevant data that were charted that relate to the review questions and objectives. | Available on request |
| Synthesis of results | 18 | Summarize and/or present the charting results as they relate to the review questions and objectives. | 11-13 and Appendix 6 |
| **DISCUSSION** | | | |
| Summary of evidence | 19 | Summarize the main results (including an overview of concepts, themes, and types of evidence available), link to the review questions and objectives, and consider the relevance to key groups. | 14-15 |
| Limitations | 20 | Discuss the limitations of the scoping review process. | 14-15 |
| Conclusions | 21 | Provide a general interpretation of the results with respect to the review questions and objectives, as well as potential implications and/or next steps. | 14-15 |
| **FUNDING** | | | |
| Funding | 22 | Describe sources of funding for the included sources of evidence, as well as sources of funding for the scoping review. Describe the role of the funders of the scoping review. | 17 |

JBI = Joanna Briggs Institute; PRISMA-ScR = Preferred Reporting Items for Systematic reviews and Meta-Analyses extension for Scoping Reviews.

* Where *sources of evidence* (see second footnote) are compiled from, such as bibliographic databases, social media platforms, and Web sites.

† A more inclusive/heterogeneous term used to account for the different types of evidence or data sources (e.g., quantitative and/or qualitative research, expert opinion, and policy documents) that may be eligible in a scoping review as opposed to only studies. This is not to be confused with *information sources* (see first footnote).

‡ The frameworks by Arksey and O’Malley (6) and Levac and colleagues (7) and the JBI guidance (4, 5) refer to the process of data extraction in a scoping review as data charting*.*

§ The process of systematically examining research evidence to assess its validity, results, and relevance before using it to inform a decision. This term is used for items 12 and 19 instead of "risk of bias" (which is more applicable to systematic reviews of interventions) to include and acknowledge the various sources of evidence that may be used in a scoping review (e.g., quantitative and/or qualitative research, expert opinion, and policy document).

*From:* Tricco AC, Lillie E, Zarin W, O'Brien KK, Colquhoun H, Levac D, et al. PRISMA Extension for Scoping Reviews (PRISMA-ScR): Checklist and Explanation. Ann Intern Med. ;169:467–473. doi: 10.7326/M18-0850

# Appendix 2: Database search strategies

## MEDLINE version (adapted for other databases).

| **Search Number** | **Query** |
| --- | --- |
| 1 | Stroke/ |
| 2 | stroke.mp. |
| 3 | cerebrovascular event.mp. |
| 4 | cerebrovascular accident.mp. |
| 5 | Atrial Fibrillation/ |
| 6 | atrial fibrillation.mp. |
| 7 | Heart Valve Diseases/ |
| 8 | valvular heart disease.mp. |
| 9 | structural heart disease.mp. |
| 10 | Peripheral Vascular Diseases/ |
| 11 | peripheral vascular disease.mp. |
| 12 | Anticoagulants/ |
| 13 | anticoagu*.mp. |
| 14 | Warfarin/ |
| 15 | warfarin.mp. |
| 16 | non-vitamin K antagonist.mp. |
| 17 | noac*.mp. |
| 18 | doac*.mp. |
| 19 | Rivaroxaban/ |
| 20 | rivaroxaban.mp. |
| 21 | apixaban.mp. |
| 22 | Dabigatran/ |
| 23 | dabigatran.mp. |
| 24 | edoxaban.mp. |
| 25 | Factor Xa Inhibitors/ |
| 26 | Platelet Aggregation Inhibitors/ |
| 27 | platelet aggregation inhibitors.mp. |
| 28 | Aspirin/ |
| 29 | aspirin.mp. |
| 30 | antiplatelets.mp. |
| 31 | antithrombotics.mp. |
| 32 | anti-thrombotics.mp. |
| 33 | or/1-32 |
| 34 | International Normalized Ratio/ |
| 35 | international normalised ratio.mp. |
| 36 | international normalized ratio.mp. |
| 37 | inr.mp. |
| 38 | time in therapeutic range.mp. |
| 39 | ttr.mp. |
| 40 | Clinical Decision Making/ |
| 41 | clinical decision making.mp. |
| 42 | Decision Support Systems, Clinical/ |
| 43 | clinical decision support.mp. |
| 44 | Decision Support Techniques/ |
| 45 | decision aids.mp. |
| 46 | "Delivery of Health Care, Integrated"/ |
| 47 | integrated care.mp. |
| 48 | nurse led.mp. |
| 49 | nurse managed.mp. |
| 50 | pharmacist led.mp. |
| 51 | pharmacist managed.mp. |
| 52 | Pharmacists/ |
| 53 | pharmacist*.mp. |
| 54 | Pharmacy Service, Hospital/ |
| 55 | Drug Monitoring/ |
| 56 | Prescription Drug Monitoring Programs/ |
| 57 | Electronic Prescribing/ |
| 58 | Inappropriate Prescribing/ |
| 59 | Prescriptions/ |
| 60 | Electronic Health Records/ |
| 61 | electronic health records.mp. |
| 62 | Guideline/ |
| 63 | Practice Guideline/ |
| 64 | Practice Guidelines as Topic/ |
| 65 | Guideline Adherence/ |
| 66 | Medication Adherence/ |
| 67 | "Treatment Adherence and Compliance"/ |
| 68 | Interdisciplinary Communication/ |
| 69 | Patient Education as Topic/ |
| 70 | Practice Patterns, Physicians'/ |
| 71 | Reminder Systems/ |
| 72 | Patient Compliance/ |
| 73 | Risk Management/ |
| 74 | Telemedicine/ |
| 75 | Self Care/ |
| 76 | self monitoring.mp. |
| 77 | Self-Management/ |
| 78 | self management.mp. |
| 79 | or/34-78 |
| 80 | Randomized Controlled Trial/ |
| 81 | Clinical Trial/ |
| 82 | randomised controlled trial.mp. |
| 83 | randomized controlled trial.mp. |
| 84 | randomised trial.mp. |
| 85 | randomized trial.mp. |
| 86 | Retrospective Studies/ |
| 87 | Prospective Studies/ |
| 88 | Follow-Up Studies/ |
| 89 | "before and after".mp. |
| 90 | Evaluation Studies/ |
| 91 | Program Evaluation/ |
| 92 | Evaluation Studies as Topic/ |
| 93 | Treatment Outcome/ |
| 94 | "Outcome Assessment (Health Care)"/ |
| 95 | Patient Outcome Assessment/ |
| 96 | Problem-Based Learning/ |
| 97 | Implementation Science/ |
| 98 | Quality Improvement/ |
| 99 | or/80-98 |
| 100 | 33 and 79 and 99 |
| 101 | limit 100 to yr="2000 -Current" |

## EMBASE (OVID) search strategy

| 1 | Stroke/ |
| --- | --- |
| 2 | stroke.mp. |
| 3 | cerebrovascular event.mp. |
| 4 | cerebrovascular accident.mp. |
| 5 | Atrial Fibrillation/ |
| 6 | atrial fibrillation.mp. |
| 7 | Heart Valve Diseases/ |
| 8 | valvular heart disease.mp. |
| 9 | structural heart disease.mp. |
| 10 | Peripheral Vascular Diseases/ |
| 11 | peripheral vascular disease.mp. |
| 12 | Anticoagulants/ |
| 13 | anticoagu*.mp. |
| 14 | Warfarin/ |
| 15 | warfarin.mp. |
| 16 | non-vitamin K antagonist.mp. |
| 17 | noac*.mp. |
| 18 | doac*.mp. |
| 19 | Rivaroxaban/ |
| 20 | rivaroxaban.mp. |
| 21 | apixaban.mp. |
| 22 | Dabigatran/ |
| 23 | dabigatran.mp. |
| 24 | edoxaban.mp. |
| 25 | Factor Xa Inhibitors/ |
| 26 | Platelet Aggregation Inhibitors/ |
| 27 | platelet aggregation inhibitors.mp. |
| 28 | Aspirin/ |
| 29 | aspirin.mp. |
| 30 | antiplatelets.mp. |
| 31 | antithrombotics.mp. |
| 32 | anti-thrombotics.mp. |
| 33 | 1 or 2 or 3 or 4 or 5 or 6 or 7 or 8 or 9 or 10 or 11 or 12 or 13 or 14 or 15 or 16 or 17 or 18 or 19 or 20 or 21 or 22 or 23 or 24 or 25 or 26 or 27 or 28 or 29 or 30 or 31 or 32 |
| 34 | International Normalized Ratio/ |
| 35 | international normalised ratio.mp. |
| 36 | international normalized ratio.mp. |
| 37 | inr.mp. |
| 38 | time in therapeutic range.mp. |
| 39 | ttr.mp. |
| 40 | Clinical Decision Making/ |
| 41 | clinical decision making.mp. |
| 42 | Decision Support Systems, Clinical/ |
| 43 | clinical decision support.mp. |
| 44 | Decision Support Techniques/ |
| 45 | decision aids.mp. |
| 46 | Delivery of Health Care, Integrated/ |
| 47 | integrated care.mp. |
| 48 | nurse led.mp. |
| 49 | nurse managed.mp. |
| 50 | pharmacist led.mp. |
| 51 | pharmacist managed.mp. |
| 52 | Pharmacists/ |
| 53 | pharmacist*.mp. |
| 54 | Pharmacy Service, Hospital/ |
| 55 | Drug Monitoring/ |
| 56 | Prescription Drug Monitoring Programs/ |
| 57 | Electronic Prescribing/ |
| 58 | Inappropriate Prescribing/ |
| 59 | Prescriptions/ |
| 60 | Electronic Health Records/ |
| 61 | electronic health records.mp. |
| 62 | Guideline/ |
| 63 | Practice Guideline/ |
| 64 | Practice Guidelines as Topic/ |
| 65 | Guideline Adherence/ |
| 66 | Medication Adherence/ |
| 67 | Treatment Adherence and Compliance/ |
| 68 | Interdisciplinary Communication/ |
| 69 | Patient Education as Topic/ |
| 70 | Practice Patterns, Physicians'/ |
| 71 | Reminder Systems/ |
| 72 | Patient Compliance/ |
| 73 | Risk Management/ |
| 74 | Telemedicine/ |
| 75 | Self Care/ |
| 76 | self monitoring.mp. |
| 77 | Self-Management/ |
| 78 | self management.mp. |
| 79 | 34 or 35 or 36 or 37 or 38 or 39 or 40 or 41 or 42 or 43 or 44 or 45 or 46 or 47 or 48 or 49 or 50 or 51 or 52 or 53 or 54 or 55 or 56 or 57 or 58 or 59 or 60 or 61 or 62 or 63 or 64 or 65 or 66 or 67 or 68 or 69 or 70 or 71 or 72 or 73 or 74 or 75 or 76 or 77 or 78 |
| 80 | Randomized Controlled Trial/ |
| 81 | Clinical Trial/ |
| 82 | randomised controlled trial.mp. |
| 83 | randomized controlled trial.mp. |
| 84 | randomised trial.mp. |
| 85 | randomized trial.mp. |
| 86 | Retrospective Studies/ |
| 87 | Prospective Studies/ |
| 88 | Follow-Up Studies/ |
| 89 | before and after.mp. |
| 90 | Evaluation Studies/ |
| 91 | Program Evaluation/ |
| 92 | Evaluation Studies as Topic/ |
| 93 | Treatment Outcome/ |
| 94 | Outcome Assessment (Health Care)/ |
| 95 | Patient Outcome Assessment/ |
| 96 | Problem-Based Learning/ |
| 97 | Implementation Science/ |
| 98 | Quality Improvement/ |
| 99 | 80 or 81 or 82 or 83 or 84 or 85 or 86 or 87 or 88 or 89 or 90 or 91 or 92 or 93 or 94 or 95 or 96 or 97 or 98 |
| 100 | 33 and 79 and 99 |
| 101 | limit 100 to yr="2000 -Current" |

## CINAHL Ultimate (EBSCOhost) search strategy

| S1 | (MH Stroke) |
| --- | --- |
| S2 | stroke |
| S3 | cerebrovascular event |
| S4 | cerebrovascular accident |
| S5 | (MH Atrial Fibrillation) |
| S6 | atrial fibrillation |
| S7 | (MH Heart Valve Diseases) |
| S8 | valvular heart disease |
| S9 | structural heart disease |
| S10 | (MH Peripheral Vascular Diseases) |
| S11 | peripheral vascular disease |
| S12 | (MH Anticoagulants) |
| S13 | anticoagu* |
| S14 | (MH Warfarin) |
| S15 | warfarin |
| S16 | non-vitamin K antagonist |
| S17 | noac* |
| S18 | doac* |
| S19 | (MH Rivaroxaban) |
| S20 | rivaroxaban |
| S21 | apixaban |
| S22 | (MH Dabigatran) |
| S23 | dabigatran |
| S24 | edoxaban |
| S25 | (MH Factor Xa Inhibitors) |
| S26 | ( MH Platelet Aggregation Inhibitors) |
| S27 | platelet aggregation inhibitors |
| S28 | (MH Aspirin) |
| S29 | aspirin |
| S30 | antiplatelet |
| S31 | antithrombotic |
| S32 | anti-thrombotics |
| S33 | S1 OR S2 OR S3 OR S4 OR S5 OR S6 OR S7 OR S8 OR S9 OR S10 OR S11 OR S12 OR S13 OR S14 OR S15 OR S16 OR S17 OR S18 OR S19 OR S20 OR S21 OR S22 OR S23 OR S24 OR S25 OR S26 OR S27 OR S28 OR S29 OR S30 OR S31 OR S32 |
| S34 | (MH International Normalized Ratio) |
| S35 | international normalised ratio |
| S36 | international normalized ratio |
| S37 | inr |
| S38 | time in therapeutic range |
| S39 | ttr |
| S40 | (MH Clinical Decision Making) |
| S41 | clinical decision making |
| S42 | (MH Decision Support Systems, Clinical) |
| S43 | clinical decision support |
| S44 | (MH Decision Support Techniques) |
| S45 | decision aids |
| S46 | (MH "Delivery of Health Care, Integrated") |
| S47 | integrated care |
| S48 | nurse led |
| S49 | nurse manager |
| S50 | pharmacist led |
| S51 | pharmacist managed |
| S52 | (MH Pharmacists) |
| S53 | pharmacist* |
| S54 | (MH Pharmacy Service, Hospital) |
| S55 | (MH Drug Monitoring) |
| S56 | (MH Prescription Drug Monitoring Programs) |
| S57 | electronic prescribing |
| S58 | (MH Inappropriate Prescribing) |
| S59 | (MH Prescriptions) |
| S60 | (MH Electronic Health Records) |
| S61 | electronic health records |
| S62 | (MH Guideline) |
| S63 | (MH Practice Guideline) |
| S64 | (MH Practice Guidelines as Topic) |
| S65 | (MH Guideline Adherence) |
| S66 | (MH Medication Adherence) |
| S67 | (MH "Treatment Adherence and Compliance") |
| S68 | (MH Interdisciplinary Communication) |
| S69 | (MH Patient Education as Topic) |
| S70 | (MH Practice Patterns, Physicians') |
| S71 | (MH Reminder Systems) |
| S72 | (MH Patient Compliance) |
| S73 | (MH Risk Management) |
| S74 | (MH Telemedicine) |
| S75 | (MH Self Care) |
| S76 | self monitoring |
| S77 | (MH Self-Management) |
| S78 | self management |
| S79 | S34 OR S35 OR S36 OR S37 OR S38 OR S39 OR S40 OR S41 OR S42 OR S43 OR S44 OR S45 OR S46 OR S47 OR S48 OR S49 OR S50 OR S51 OR S52 OR S53 OR S54 OR S55 OR S56 OR S57 OR S58 OR S59 OR S60 OR S61 OR S62 OR S63 OR S64 OR S65 OR S66 OR S67 OR S68 OR S69 OR S70 OR S71 OR S72 OR S73 OR S74 OR S75 OR S76 OR S77 OR S78 |
| S80 | (MH Randomized Controlled Trial) |
| S81 | (MH Clinical Trial) |
| S82 | randomised controlled trial |
| S83 | randomized controlled trials |
| S84 | randomised trial |
| S85 | randomized trial |
| S86 | (MH Retrospective Studies) |
| S87 | (MH Prospective Studies) |
| S88 | (MH Follow-Up Studies) |
| S89 | "before and after" |
| S90 | (MH Evaluation Studies) |
| S91 | (MH Program Evaluation) |
| S92 | (MH Evaluation Studies as Topic) |
| S93 | (MH Treatment Outcome) |
| S94 | (MH "Outcome Assessment (Health Care)") |
| S95 | (MH Patient Outcome Assessment) |
| S96 | (MH Problem-Based Learning) |
| S97 | (MH Implementation Science) |
| S98 | (MH Quality Improvement) |
| S99 | S80 OR S81 OR S82 OR S83 OR S84 OR S85 OR S86 OR S87 OR S88 OR S89 OR S90 OR S91 OR S92 OR S93 OR S94 OR S95 OR S96 OR S97 OR S98 |
| S100 | S33 and S79 and S99 Date limited to 2000- |

## PsycINFO (EBSCOhost) search strategy

| S1 | (MH Stroke) |
| --- | --- |
| S2 | stroke |
| S3 | cerebrovascular event |
| S4 | cerebrovascular accident |
| S5 | (MH Atrial Fibrillation) |
| S6 | atrial fibrillation |
| S7 | (MH Heart Valve Diseases) |
| S8 | valvular heart disease |
| S9 | structural heart disease |
| S10 | (MH Peripheral Vascular Diseases) |
| S11 | peripheral vascular disease |
| S12 | (MH Anticoagulants) |
| S13 | anticoagu* |
| S14 | (MH Warfarin) |
| S15 | warfarin |
| S16 | non-vitamin K antagonist |
| S17 | noac* |
| S18 | doac* |
| S19 | (MH Rivaroxaban) |
| S20 | rivaroxaban |
| S21 | apixaban |
| S22 | (MH Dabigatran) |
| S23 | dabigatran |
| S24 | edoxaban |
| S25 | (MH Factor Xa Inhibitors) |
| S26 | ( MH Platelet Aggregation Inhibitors) |
| S27 | platelet aggregation inhibitors |
| S28 | (MH Aspirin) |
| S29 | aspirin |
| S30 | antiplatelet |
| S31 | antithrombotics |
| S32 | anti-thrombotics |
| S33 | S1 OR S2 OR S3 OR S4 OR S5 OR S6 OR S7 OR S8 OR S9 OR S10 OR S11 OR S12 OR S13 OR S14 OR S15 OR S16 OR S17 OR S18 OR S19 OR S20 OR S21 OR S22 OR S23 OR S24 OR S25 OR S26 OR S27 OR S28 OR S29 OR S30 OR S31 OR S32 |
| S34 | (MH International Normalized Ratio) |
| S35 | international normalized ratio |
| S36 | international normalised ratio |
| S37 | inr |
| S38 | time in therapeutic range |
| S39 | ttr |
| S40 | (MH Clinical Decision Making) |
| S41 | clinical decision making |
| S42 | (MH Decision Support Systems, Clinical) |
| S43 | clinical decision support |
| S44 | (MH Decision Support Techniques) |
| S45 | decision aids |
| S46 | (MH "Delivery of Health Care, Integrated") |
| S47 | integrated care |
| S48 | nurse led |
| S49 | nurse managed |
| S50 | pharmacist led |
| S51 | pharmacist managed |
| S52 | (MH Pharmacists) |
| S53 | pharmacist* |
| S54 | (MH Pharmacy Service, Hospital) |
| S55 | (MH Drug Monitoring) |
| S56 | (MH Prescription Drug Monitoring Programs) |
| S57 | (MH Electronic Prescribing) |
| S58 | (MH Inappropriate Prescribing) |
| S59 | (MH Prescriptions) |
| S60 | (MH Electronic Health Records) |
| S61 | electronic health records |
| S62 | (MH Guideline) |
| S63 | (MH Practice Guideline) |
| S64 | (MH Practice Guidelines as Topic) |
| S65 | (MH Guideline Adherence) |
| S66 | (MH Medication Adherence) |
| S67 | (MH "Treatment Adherence and Compliance") |
| S68 | (MH Interdisciplinary Communication) |
| S69 | (MH Patient Education as Topic) |
| S70 | (MH Practice Patterns, Physicians') |
| S71 | (MH Reminder Systems) |
| S72 | (MH Patient Compliance) |
| S73 | (MH Risk Management) |
| S74 | (MH Telemedicine) |
| S75 | (MH Self Care) |
| S76 | self monitoring |
| S77 | (MH Self-Management) |
| S78 | self management |
| S79 | S34 OR S35 OR S36 OR S37 OR S38 OR S39 OR S40 OR S41 OR S42 OR S43 OR S44 OR S45 OR S46 OR S47 OR S48 OR S49 OR S50 OR S51 OR S52 OR S53 OR S54 OR S55 OR S56 OR S57 OR S58 OR S59 OR S60 OR S61 OR S62 OR S63 OR S64 OR S65 OR S66 OR S67 OR S68 OR S69 OR S70 OR S71 OR S72 OR S73 OR S74 OR S75 OR S76 OR S77 OR S78 |
| S80 | (MH Randomized Controlled Trial) |
| S81 | (MH Clinical Trial) |
| S82 | randomised controlled trial |
| S83 | randomized controlled trials |
| S84 | randomised trial |
| S85 | randomized trial |
| S86 | (MH Retrospective Studies) |
| S87 | (MH Prospective Studies) |
| S88 | (MH Follow-Up Studies) |
| S89 | "before and after" |
| S90 | (MH Evaluation Studies) |
| S91 | (MH Program Evaluation) |
| S92 | (MH Evaluation Studies as Topic) |
| S93 | (MH Treatment Outcome) |
| S94 | (MH "Outcome Assessment (Health Care)") |
| S95 | (MH Patient Outcome Assessment) |
| S96 | (MH Problem-Based Learning) |
| S97 | (MH Implementation Science) |
| S98 | (MH Quality Improvement) |
| S99 | S80 OR S81 OR S82 OR S83 OR S84 OR S85 OR S86 OR S87 OR S88 OR S89 OR S90 OR S91 OR S92 OR S93 OR S94 OR S95 OR S96 OR S97 OR S98 |
| S100 | S33 and S79 and S99 Date limited to 2000- |

## Cochrane Database of Systematic Reviews (via Wiley) search strategy

| #1 | MeSH descriptor: [Stroke] explode all trees |
| --- | --- |
| #2 | stroke |
| #3 | cerebrovascular event |
| #4 | cerebrovascular accident |
| #5 | MeSH descriptor: [Atrial Fibrillation] explode all trees |
| #6 | atrial fibrillation |
| #7 | MeSH descriptor: [Heart Valve Diseases] explode all trees |
| #8 | valvular heart disease |
| #9 | structural heart disease |
| #10 | MeSH descriptor: [Peripheral Vascular Diseases] explode all trees |
| #11 | peripheral vascular disease |
| #12 | MeSH descriptor: [Anticoagulants] explode all trees |
| #13 | anticoagu* |
| #14 | MeSH descriptor: [Warfarin] explode all trees |
| #15 | warfarin |
| #16 | non-vitamin K antagonist |
| #17 | noac* |
| #18 | doac* |
| #19 | MeSH descriptor: [Rivaroxaban] explode all trees |
| #20 | rivaroxaban |
| #21 | apixaban |
| #22 | MeSH descriptor: [Dabigatran] explode all trees |
| #23 | dabigatran |
| #24 | edoxaban |
| #25 | MeSH descriptor: [Factor Xa Inhibitors] explode all trees |
| #26 | MeSH descriptor: [Platelet Aggregation Inhibitors] explode all trees |
| #27 | platelet aggregation inhibitors |
| #28 | MeSH descriptor: [Aspirin, Dipyridamole Drug Combination] explode all trees |
| #29 | aspirin |
| #30 | antiplatelets |
| #31 | antithrombotics |
| #32 | anti-thrombotics |
| #33 | {or #1-#32} |
| #34 | MeSH descriptor: [International Normalized Ratio] explode all trees |
| #35 | international normalised ratio |
| #36 | international normalized ratio |
| #37 | inr |
| #38 | time in therapeutic range |
| #39 | ttr |
| #40 | MeSH descriptor: [Clinical Decision-Making] explode all trees |
| #41 | clinical decision making |
| #42 | MeSH descriptor: [Decision Support Systems, Clinical] explode all trees |
| #43 | clinical decision support |
| #44 | MeSH descriptor: [Decision Support Techniques] explode all trees |
| #45 | decision aids |
| #46 | MeSH descriptor: [Delivery of Health Care, Integrated] explode all trees |
| #47 | integrated care |
| #48 | nurse led |
| #49 | nurse managed |
| #50 | pharmacist led |
| #51 | pharmacist managed |
| #52 | MeSH descriptor: [Pharmacists] explode all trees |
| #53 | pharmacist* |
| #54 | MeSH descriptor: [Pharmacy Service, Hospital] 3 tree(s) exploded |
| #55 | MeSH descriptor: [Drug Monitoring] explode all trees |
| #56 | MeSH descriptor: [Prescription Drug Monitoring Programs] explode all trees |
| #57 | MeSH descriptor: [Electronic Prescribing] explode all trees |
| #58 | MeSH descriptor: [Inappropriate Prescribing] explode all trees |
| #59 | MeSH descriptor: [Prescriptions] explode all trees |
| #60 | MeSH descriptor: [Electronic Health Records] 1 tree(s) exploded |
| #61 | electronic health records |
| #62 | MeSH descriptor: [Guideline] explode all trees |
| #63 | MeSH descriptor: [Practice Guideline] explode all trees |
| #64 | MeSH descriptor: [Practice Guidelines as Topic] explode all trees |
| #65 | MeSH descriptor: [Guideline Adherence] explode all trees |
| #66 | MeSH descriptor: [Medication Adherence] explode all trees |
| #67 | MeSH descriptor: [Treatment Adherence and Compliance] explode all trees |
| #68 | MeSH descriptor: [Interdisciplinary Communication] explode all trees |
| #69 | MeSH descriptor: [Patient Education as Topic] explode all trees |
| #70 | MeSH descriptor: [Practice Patterns, Physicians'] explode all trees |
| #71 | MeSH descriptor: [Reminder Systems] explode all trees |
| #72 | MeSH descriptor: [Patient Compliance] explode all trees |
| #73 | MeSH descriptor: [Risk Management] explode all trees |
| #74 | MeSH descriptor: [Telemedicine] 3 tree(s) exploded |
| #75 | MeSH descriptor: [Self Care] explode all trees |
| #76 | self monitoring |
| #77 | MeSH descriptor: [Self-Management] explode all trees |
| #78 | self management |
| #79 | {or #34- #78} |
| #80 | MeSH descriptor: [Randomized Controlled Trial] explode all trees |
| #81 | MeSH descriptor: [Clinical Trial] explode all trees |
| #82 | randomised controlled trial |
| #83 | randomized controlled trial |
| #84 | randomised trial |
| #85 | randomized trial |
| #86 | MeSH descriptor: [Retrospective Studies] explode all trees |
| #87 | MeSH descriptor: [Prospective Studies] explode all trees |
| #88 | MeSH descriptor: [Follow-Up Studies] explode all trees |
| #89 | before and after |
| #90 | MeSH descriptor: [Evaluation Study] explode all trees |
| #91 | MeSH descriptor: [Program Evaluation] explode all trees |
| #92 | MeSH descriptor: [Evaluation Studies as Topic] explode all trees |
| #93 | MeSH descriptor: [Treatment Outcome] explode all trees |
| #94 | MeSH descriptor: [Outcome Assessment, Health Care] explode all trees |
| #95 | MeSH descriptor: [Patient Outcome Assessment] explode all trees |
| #96 | MeSH descriptor: [Problem-Based Learning] explode all trees |
| #97 | MeSH descriptor: [Implementation Science] explode all trees |
| #98 | MeSH descriptor: [Quality Improvement] explode all trees |
| #99 | {or #80- #98} |
| #100 | #33 and #79 and #99 Limited by date 2000- |

# Appendix 3: Definition of each of the 73 ERIC strategies and their mapping into 9 clusters (as per Waltz et al. 2015).

| **Cluster** | **ERIC strategy** | **Definition** |
| --- | --- | --- |
| Train and educate stakeholders: 11 strategies | Conduct educational meetings | Hold meetings targeted toward different stakeholder groups (*e.g.*, providers, administrators, other organizational stakeholders, and community, patient/consumer, and family stakeholders) to teach them about the clinical innovation |
|  | Conduct educational outreach visits | Have a trained person meet with providers in their practice settings to educate providers about the clinical innovation with the intent of changing the provider’s practice |
|  | Conduct ongoing training | Plan for and conduct training in the clinical innovation in an ongoing way |
|  | Create a learning collaborative | Facilitate the formation of groups of providers or provider organizations and foster a collaborative learning environment to improve implementation of the clinical innovation |
|  | Develop educational materials | Develop and format manuals, toolkits, and other supporting materials in ways that make it easier for stakeholders to learn about the innovation and for clinicians to learn how to deliver the clinical innovation |
|  | Distribute educational materials | Distribute educational materials (including guidelines, manuals, and toolkits) in person, by mail, and/or electronically |
|  | Make training dynamic | Vary the information delivery methods to cater to different learning styles and work contexts, and shape the training in the innovation to be interactive |
|  | Provide ongoing consultation | Provide ongoing consultation with one or more experts in the strategies used to support implementing the innovation |
|  | Shadow other experts | Provide ways for key individuals to directly observe experienced people engage with or use the targeted practice change/innovation |
|  | Use train-the-trainer strategies | Train designated clinicians or organizations to train others in the clinical innovation |
|  | Work with educational institutions | Encourage educational institutions to train clinicians in the innovation |
| Provide interactive assistance: 4 strategies | Centralize technical assistance | Develop and use a centralized system to deliver technical assistance focused on implementation issues |
|  | Facilitation | A process of interactive problem solving and support that occurs in a context of a recognized need for improvement and a supportive interpersonal relationship |
|  | Provide clinical supervision | Provide clinicians with ongoing supervision focusing on the innovation. Provide training for clinical supervisors who will supervise clinicians who provide the innovation |
|  | Provide local technical assistance | Develop and use a system to deliver technical assistance focused on implementation issues using local personnel |
| Support Clinicians: 5 strategies | Create new clinical teams | Change who serves on the clinical team, adding different disciplines and different skills to make it more likely that the clinical innovation is delivered (or is more successfully delivered) |
|  | Develop resource sharing agreements | Develop partnerships with organizations that have resources needed to implement the innovation |
|  | Facilitate relay of clinical data to providers | Provide as close to real-time data as possible about key measures of process/outcomes using integrated modes/channels of communication in a way that promotes use of the targeted innovation |
|  | Remind clinicians | Develop reminder systems designed to help clinicians to recall information and/or prompt them to use the clinical innovation |
|  | Revise professional roles | Shift and revise roles among professionals who provide care, and redesign job characteristics |
| Utilise financial strategies: 9 strategies | Alter patient/consumer fees | Create fee structures where patients/consumers pay less for preferred treatments (the clinical innovation) and more for less-preferred treatments |
|  | Alter incentive/allowance structures | Work to incentivize the adoption and implementation of the clinical innovation |
|  | Access new funding | Access new or existing money to facilitate the implementation |
|  | Develop disincentives | Provide financial disincentives for failure to implement or use the clinical innovations |
|  | Fund and contract for the clinical innovation | Governments and other payers of services issue requests for proposals to deliver the innovation, use contracting processes to motivate providers to deliver the clinical innovation, and develop new funding formulas that make it more likely that providers will deliver the innovation |
|  | Make billing easier | Make it easier to bill for the clinical innovation |
|  | Place innovation on fee for service lists/formularies | Work to place the clinical innovation on lists of actions for which providers can be reimbursed (*e.g.*, a drug is placed on a formulary, a procedure is now reimbursable) |
|  | Use capitated payments | Pay providers or care systems a set amount per patient/consumer for delivering clinical care |
|  | Use other payment schemes | Introduce payment approaches (in a catch-all category) |
| Develop stakeholder interrelationships;16 strategies | Build a coalition | Recruit and cultivate relationships with partners in the implementation effort |
|  | Capture and share local knowledge | Capture local knowledge from implementation sites on how implementers and clinicians made something work in their setting and then share it with other sites |
|  | Conduct local consensus discussions | Include local providers and other stakeholders in discussions that address whether the chosen problem is important and whether the clinical innovation to address it is appropriate |
|  | Develop academic partnerships | Partner with a university or academic unit for the purposes of shared training and bringing research skills to an implementation project |
|  | Develop an implementation glossary | Develop and distribute a list of terms describing the innovation, implementation, and stakeholders in the organizational change |
|  | Identify and prepare champions | Identify and prepare individuals who dedicate themselves to supporting, marketing, and driving through an implementation, overcoming indifference or resistance that the intervention may provoke in an organization |
|  | Identify early adopters | Identify early adopters at the local site to learn from their experiences with the practice innovation |
|  | Inform local opinion leaders | Inform providers identified by colleagues as opinion leaders or “educationally influential” about the clinical innovation in the hopes that they will influence colleagues to adopt it |
|  | Involve executive boards | Involve existing governing structures (*e.g.*, boards of directors, medical staff boards of governance) in the implementation effort, including the review of data on implementation processes |
|  | Model and simulate change | Model or simulate the change that will be implemented prior to implementation |
|  | Obtain formal commitments | Obtain written commitments from key partners that state what they will do to implement the innovation |
|  | Organize clinician implementation team meetings | Develop and support teams of clinicians who are implementing the innovation and give them protected time to reflect on the implementation effort, share lessons learned, and support one another’s learning |
|  | Promote network weaving | Identify and build on existing high-quality working relationships and networks within and outside the organization, organizational units, teams, etc. to promote information sharing, collaborative problem-solving, and a shared vision/goal related to implementing the innovation |
|  | Recruit, designate, and train for leadership | Recruit, designate, and train leaders for the change effort |
|  | Use advisory boards and workgroups | Create and engage a formal group of multiple kinds of stakeholders to provide input and advice on implementation efforts and to elicit recommendations for improvements |
|  | Use an implementation advisor | Seek guidance from experts in implementation |
|  | Visit other sites | Visit sites where a similar implementation effort has been considered successful |
| Adapt and tailor to the context: 4 strategies | Promote adaptability | Identify the ways a clinical innovation can be tailored to meet local needs and clarify which elements of the innovation must be maintained to preserve fidelity |
|  | Tailor strategies | Tailor the implementation strategies to address barriers and leverage facilitators that were identified through earlier data collection |
|  | Use data experts | Involve, hire, and/or consult experts to inform management on the use of data generated by implementation efforts |
|  | Use data warehousing techniques | Integrate clinical records across facilities and organizations to facilitate implementation across systems |
| Change infrastructure: 8 strategies | Change accreditation or membership requirements | Strive to alter accreditation standards so that they require or encourage use of the clinical innovation. Work to alter membership organization requirements so that those who want to affiliate with the organization are encouraged or required to use the clinical innovation |
|  | Change liability laws | Participate in liability reform efforts that make clinicians more willing to deliver the clinical innovation |
|  | Change physical structure and equipment | Evaluate current configurations and adapt, as needed, the physical structure and/or equipment (*e.g.*, changing the layout of a room, adding equipment) to best accommodate the targeted innovation |
|  | Change record systems | Change records systems to allow better assessment of implementation or clinical outcomes |
|  | Change service sites | Change the location of clinical service sites to increase access |
|  | Create or change credentialing and/or licensure standards | Create an organization that certifies clinicians in the innovation or encourage an existing organization to do so. Change governmental professional certification or licensure requirements to include delivering the innovation. Work to alter continuing education requirements to shape professional practice toward the innovation |
|  | Mandate change | Have leadership declare the priority of the innovation and their determination to have it implemented |
|  | Start a dissemination organization | Identify or start a separate organization that is responsible for disseminating the clinical innovation. It could be a for-profit or non-profit organization |
| Use evaluative and iterative strategies: 10 strategies | Assess for readiness and identify barriers and facilitators | Assess various aspects of an organization to determine its degree of readiness to implement, barriers that may impede implementation, and strengths that can be used in the implementation effort |
|  | Audit and provide feedback | Collect and summarize clinical performance data over a specified time period and give it to clinicians and administrators to monitor, evaluate, and modify provider behaviour |
|  | Conduct cyclical small tests of change | Implement changes in a cyclical fashion using small tests of change before taking changes system-wide. Tests of change benefit from systematic measurement, and results of the tests of change are studied for insights on how to do better. This process continues serially over time, and refinement is added with each cycle |
|  | Conduct local needs assessment | Collect and analyse data related to the need for the innovation |
|  | Develop a formal implementation blueprint | Develop a formal implementation blueprint that includes all goals and strategies. The blueprint should include the following: 1) aim/purpose of the implementation; 2) scope of the change (*e.g.*, what organizational units are affected); 3) timeframe and milestones; and 4) appropriate performance/progress measures. Use and update this plan to guide the implementation effort over time |
|  | Develop and implement tools for quality monitoring | Develop, test, and introduce into quality-monitoring systems the right input—the appropriate language, protocols, algorithms, standards, and measures (of processes, patient/consumer outcomes, and implementation outcomes) that are often specific to the innovation being implemented |
|  | Develop and organize quality monitoring systems | Develop and organize systems and procedures that monitor clinical processes and/or outcomes for the purpose of quality assurance and improvement |
|  | Obtain and use patients/consumers and family feedback | Develop strategies to increase patient/consumer and family feedback on the implementation effort |
|  | Purposely reexamine the implementation | Monitor progress and adjust clinical practices and implementation strategies to continuously improve the quality of care |
|  | Stage implementation scale up | Phase implementation efforts by starting with small pilots or demonstration projects and gradually move to a system wide rollout |
| Engage consumers: 5 strategies | Increase demand | Attempt to influence the market for the clinical innovation to increase competition intensity and to increase the maturity of the market for the clinical innovation |
|  | Intervene with patients/consumers to enhance uptake and adherence | Develop strategies with patients to encourage and problem solve around adherence |
|  | Involve patients/consumers and family members | Engage or include patients/consumers and families in the implementation effort |
|  | Prepare patients/consumers to be active participants | Prepare patients/consumers to be active in their care, to ask questions, and specifically to inquire about care guidelines, the evidence behind clinical decisions, or about available evidence-supported treatments |
|  | Use mass media | Use media to reach large numbers of people to spread the word about the clinical innovation |

**Appendix 4:** References to included and excluded studies

**Included studies; references for n= 245 reports (includes sister/ related papers*)**

1. Ahmed NO, Osman B, Abdelhai YM, El-Hadiyah TMH. Impact of clinical pharmacist intervention in anticoagulation clinic in Sudan. International Journal of Clinical Pharmacy. 2017;39(4):769-73.
2. Al-Mahroos MI, Abdulridha MK, Alhaleem MR. Evaluation of a designed community-based post -discharge warfarin management protocol on Iraqi patients. International Journal of Pharmaceutical Sciences and Research. 2017;8(5):2323-32.
3. Alanazi Z, Almutairi N, AlDukkan L, Arafat AA, Albabtain MA. Time in therapeutic range for virtual anticoagulation clinic versus in-person clinic during the COVID-19 pandemic: a crossover study. Ann Saudi Med. 2022;42(5):305-8.
4. Alhmoud EN, Abd El Samad OB, Elewa H, Alkhozondar O, Soaly E, El Anany R. Drive-up INR testing and phone-based consultations service during COVID-19 pandemic in a pharmacist-lead anticoagulation clinic in Qatar: Monitoring, clinical, resource utilization, and patient- oriented outcomes. J Am Coll Clin Pharm. 2021;4(9):1117-25.
5. Alshaiban A, Alavudeen SS, Alshahrani I, Kardam AM, Alhasan IM, Alasiri SA, et al. Impact of Clinical Pharmacist Running Anticoagulation Clinic in Saudi Arabia. J Clin Med. 2023;12(12).
6. Arts DL, Abu-Hanna A, Medlock SK, van Weert HC. Effectiveness and usage of a decision support system to improve stroke prevention in general practice: A cluster randomized controlled trial. PLoS ONE. 2017;12(2):e0170974.
7. Ashburner JM, Atlas SJ, Khurshid S, Weng LC, Hulme OL, Chang Y, et al. Electronic physician notifications to improve guideline-based anticoagulation in atrial fibrillation: a randomized controlled trial. Journal of General Internal Medicine. 2018;33(12):2070-7.
8. Ashjian E, Kurtz B, Renner E, Yeshe R, Barnes GD. Evaluation of a pharmacist-led outpatient direct oral anticoagulant service. Am J Health Syst Pharm. 2017;74(7):483-9.
9. Bajorek BV, Krass I, Ogle SJ, Duguid MJ, Shenfield GM. Optimizing the use of antithrombotic therapy for atrial fibrillation in older people: a pharmacist-led multidisciplinary intervention. J Am Geriatr Soc. 2005;53(11):1912-20.
10. Bajorek BV, Magin PJ, Hilmer SN, Krass I. Optimizing Stroke Prevention in Patients With Atrial Fibrillation: A Cluster-Randomized Controlled Trial of a Computerized Antithrombotic Risk Assessment Tool in Australian General Practice, 2012-2013. Prev Chronic Dis. 2016;13:E90.
11. Baker C, Ghassemi E, Bowers R. Benefits of Utilizing Pharmacy Learners in an Inpatient Anticoagulation Education Service. Innov Pharm. 2021;12(3).
12. Barcellona D, Contu P, Marongiu F. A "two-step" educational approach for patients taking oral anticoagulants does not improve therapy control. J Thromb Thrombolysis. 2006;22(3):185-90.
13. Barmano N, Walfridsson U, Walfridsson H, Karlsson JE. Structured care of patients with atrial fibrillation improves guideline adherence. Journal of Atrial Fibrillation. 2017;9(4):2016-7.
14. Barnes GD, Kong X, Cole D, Haymart B, Kline-Rogers E, Almany S, et al. Extended International Normalized Ratio testing intervals for warfarin-treated patients. Journal of Thrombosis and Haemostasis. 2018;16(7):1307-12.
15. Baysal E, Midilli TS. Effects of structured patient education on knowledge level and inr control of patients receiving warfarin: Randomized controlled trial. Pakistan Journal of Medical Sciences. 2018;34(2):240-426.
16. Bereznicki LR, Jackson SL, Peterson GM. Supervised patient self-testing of warfarin therapy using an online system. Journal of medical Internet research. 2013;15(7):e138.
17. Bereznicki LRE, Jackson SL, Kromdijk W, Gee P, Fitzmaurice K, Bereznicki BJ, Peterson GM. Improving the management of warfarin in aged-care facilities utilising innovative technology: A proof-of-concept study. International Journal of Pharmacy Practice. 2014;22(1):84-91.
18. Bhatt S, McCurdy CE, Liew D, Russell DM. Effect of warfarin education on patient knowledge and anticoagulation control: a randomised controlled trial. Journal of Pharmacy Practice and Research. 2018;48(6):516-21.
19. Birk SE, Ingemi A, Bourassa P, Neumann K, Pine C, Seigh M, et al. Protocol-based anticoagulation management for mechanical circulatory support patients can be safe and efficient. Int J Artif Organs. 2022;45(6):564-70.
20. Blanch P, Freixa-Pamias R, Gambau M, Lafuente R, Basile L. Impact of an oral anticoagulation self-monitoring and self-management program in patients with mechanical heart valve prosthesis. J Comp Eff Res. 2021;10(4):307-14.
21. Bo S, Valpreda S, Scaglione L, Boscolo D, Piobbici M, Bo M, Ciccone G. Implementing hospital guidelines improves warfarin use in non-valvular atrial fibrillation: a before-after study. BMC Public Health. 2007;7:203.
22. Brasen CL, Madsen JS, Parkner T, Brandslund I. Home Management of Warfarin Treatment Through a Real-Time Supervised Telemedicine Solution: A Randomized Controlled Trial. Telemedicine journal and e-health : the official journal of the American Telemedicine Association. 2019;25(2):109-15.
23. Brunetti L, Lee SM, Doherty N, Suh D, Kim JE, Lee SH, et al. Impact of warfarin discharge education program on hospital readmission and treatment costs. International Journal of Clinical Pharmacy. 2018;40(3):721-9.
24. Bussey HI, Bussey M, Bussey-Smith KL, Frei CR. Evaluation of warfarin management with international normalized ratio self-testing and online remote monitoring and management plus low-dose vitamin k with genomic considerations: a pilot study. Pharmacotherapy. 2013;33(11):1136-46.
25. Cabral A, Bonaventura KR, Milner KA. Using a Patient Portal to Expand Warfarin Self-Management. The Journal for Nurse Practitioners. 2021;17(3):344-7.
26. Cao H, Jiang S, Lv M, Wu T, Chen W, Zhang J. Effectiveness of the Alfalfa App in Warfarin Therapy Management for Patients Undergoing Venous Thrombosis Prevention and Treatment: Cohort Study. JMIR Mhealth Uhealth. 2021;9(3):e23332.
27. Cardy C, Ardisson KM, Widmar SB. Atrial fibrillation clinical decision aid for emergency medicine providers: An initiative to improve quality healthcare outcomes in adults with new-onset atrial fibrillation. Heart Lung. 2018;47(4):314-21.
28. Carter L, Gardner M, Magee K, Fearon A, Morgulis I, Doucette S, et al. An Integrated Management Approach to Atrial Fibrillation. J Am Heart Assoc. 2016;5(1):25.
29. Carver HR, Studeny M, Gress TW, Wood KA. Quality improvement in an anticoagulation clinic: development of a new treatment protocol. J Nurs Care Qual. 2012;27(2):161-70.
30. Chan FW, Wong RS, Lau WH, Chan TY, Cheng G, You JH. Management of Chinese patients on warfarin therapy in two models of anticoagulation service - a prospective randomized trial. Br J Clin Pharmacol. 2006;62(5):601-9.
31. Chartrand M, Lalonde L, Cantin A, Lahaie A, Odobasic B, Tremblay MP, et al. Anticoagulation management services in community pharmacy: Feasibility of implementing a quality improvement programme through a practice-based research network. J Clin Pharm Ther. 2018;43(6):877-87.
32. Chaturvedi S, Kelly AG, Prabhakaran S, Saposnik G, Lee L, Malik A, et al. Electronic Decision support for Improvement of Contemporary Therapy for Stroke Prevention. J Stroke Cerebrovasc Dis. 2019;28(3):569-73.
33. Chau T, Rotbard M, King S, Munoz Li M, Leong WA. Implementation and evaluation of a warfarin dosing service for rehabilitation medicine: Report from a pilot project. Canadian Journal of Hospital Pharmacy. 2006;59(3):136-47.
34. Choumane NS, Malaeb DN, Malaeb B, Hallit S. A multicenter, prospective study evaluating the impact of the clinical pharmacist-physician counselling on warfarin therapy management in Lebanon. BMC Health Serv Res. 2018;18(1):80.
35. Christensen H, Lauterlein JJ, Sorensen PD, Petersen ER, Madsen JS, Brandslund I. Home management of oral anticoagulation via telemedicine versus conventional hospital-based treatment. Telemed J E Health. 2011;17(3):169-76.
36. Christensen TD, Attermann J, Pilegaard HK, Andersen NT, Maegaard M, Hasenkam JM. Self-management of oral anticoagulant therapy for mechanical heart valve patients. Scand Cardiovasc J. 2001;35(2):107-13.
37. Christensen TD, Maegaard M, Sorensen HT, Hjortdal VE, Hasenkam JM. Self-management versus conventional management of oral anticoagulant therapy: A randomized, controlled trial. European Journal of Internal Medicine. 2006;17(4):260-6.
38. Clarkesmith DE, Pattison HM, Lip GY, Lane DA. Educational intervention improves anticoagulation control in atrial fibrillation patients: the TREAT randomised trial. PLoS ONE. 2013;8(9):e74037.
39. Cope R, Fischetti B, Eladghm N, Elaskandrany M, Karam N. Outpatient management of chronic warfarin therapy at a pharmacist-run anticoagulation clinic during the COVID-19 pandemic. J Thromb Thrombolysis. 2021;52(3):754-8.
40. Cosmi B, Palareti G, Carpanedo M, Pengo V, Biasiolo A, Rampazzo P, et al. Assessment of patient capability to self-adjust oral anticoagulant dose: a multicenter study on home use of portable prothrombin time monitor (COAGUCHECK). Haematologica. 2000;85(8):826-31.
41. Cromheecke ME, Levi M, Colly LP, de Mol BJ, Prins MH, Hutten BA, et al. Oral anticoagulation self-management and management by a specialist anticoagulation clinic: a randomised cross-over comparison. Lancet. 2000;356(9224):97-102.
42. da Silva Saraiva S, Orsi FA, Santos MP, Machado T, Montalvao S, Costa-Lima C, et al. Home management of INR in the public health system: feasibility of self-management of oral anticoagulation and long-term performance of individual POC devices in determining INR. Journal of Thrombosis and Thrombolysis. 2016;42(1):146-53.
43. Darnell T, Hughes J, Turner B, Ragheb M, Wunderlich A. Effect of a novel pharmacist-led reporting system on appropriate use of direct-acting oral anticoagulants (DOACs) in a patient-centered medical home. J Thromb Thrombolysis. 2021;51(2):413-8.
44. Das M, Panter L, Wynn GJ, Taylor RM, Connor N, Mills JD, et al. Primary Care Atrial Fibrillation Service: outcomes from consultant-led anticoagulation assessment clinics in the primary care setting in the UK. BMJ Open. 2015;5(12):e009267.
45. Davidson T, Lindelof A, Wallen T, Lindahl TL, Hallert C. Point-of-care monitoring of warfarin treatment in community dwelling elderly--A randomised controlled study. J Telemed Telecare. 2015;21(5):298-301.
46. de Assis MC, Rabelo ER, Avila CW, Polanczyk CA, Rohde LE. Improved oral anticoagulation after a dietary vitamin k-guided strategy: a randomized controlled trial. Circulation. 2009;120(12):1115-22, 3 p following 22.
47. de Castro KP, Chiu HH, De Leon-Yao RC, Almelor-Sembrana L, Dans AM. A Patient Decision Aid for Anticoagulation Therapy in Patients With Nonvalvular Atrial Fibrillation: Development and Pilot Study. JMIR Cardio. 2021;5(2):e23464.
48. Derington CG, Goodrich GK, Xu S, Clark NP, Reynolds K, An J, et al. Association of Direct Oral Anticoagulation Management Strategies With Clinical Outcomes for Adults With Atrial Fibrillation. JAMA Netw Open. 2023;6(7):e2321971.
49. Desmond D, Kogan P, Underwood S, Brobbey E, Luib P. Performance improvement in managed long-term care: improving warfarin medication management. Home Healthc Nurse. 2009;27(3):150-9.
50. Desteghe L, Vijgen J, Koopman P, D DI-B, Schurmans J, Dendale P, Heidbuchel H. Telemonitoring-based feedback improves adherence to non-Vitamin K antagonist oral anticoagulants intake in patients with atrial fibrillation. European Heart Journal. 2018;39(16):1394-403.
51. Dib JG, Mohammed K, Momattin HI, Alshehri AM. Implementation of pharmacist-managed anticoagulation clinic in a Saudi Arabian health center. Hospital Pharmacy. 2014;49(3):260-8.
52. Dietrich E, Davis K, Talana A, Holland N, Akhavan N, Panna D, et al. Three‐year clinical interventions from an outpatient multidisciplinary direct oral anticoagulant monitoring service. Jaccp: Journal of the American College of Clinical Pharmacy. 2022;5(4):406-12.
53. Dignan R, Keech AC, Gebski VJ, Mann KP, Hughes CF, Warfarin SI. Is home warfarin self-management effective? Results of the randomised Self-Management of Anticoagulation Research Trial. Int J Cardiol. 2013;168(6):5378-84.
54. Ding Y, Jiang H, Liu J, Chen D, Yang F. Effects of the theory of planned behavior and nudge strategy-based intervention on the adherence to anticoagulation treatment in patients with non-valvular atrial fibrillation. Geriatr Nurs. 2023;51:17-24.
55. Dooley MJ, McGuiness JV, Choo S, Ngo-Thai L-L, Tong E, Neave K, et al. Successful Implementation of a Pharmacist Anticoagulant Dosing Service in Ambulatory Care. Journal of Pharmacy Practice and Research. 2011;41(3):208-11.
56. Dorsch MP, Chen CS, Allen AL, Sales AE, Seagull FJ, Spoutz P, et al. Nationwide Implementation of a Population Management Dashboard for Monitoring Direct Oral Anticoagulants: Insights From the Veterans Affairs Health System. Circ Cardiovasc Qual Outcomes. 2023;16(2):e009256.
57. Dreijer AR, Kruip M, Diepstraten J, Polinder S, Brouwer R, Mol PGM, et al. Effect of antithrombotic stewardship on the efficacy and safety of antithrombotic therapy during and after hospitalization. PLoS ONE. 2020;15(6):e0235048.
58. Duff J, Walker K. Improving the safety and efficacy of warfarin therapy in a metropolitan private hospital: a multidisciplinary practice improvement project. Contemp Nurse. 2010;35(2):234-44.
59. Duran-Parrondo C, Vazquez-Lago JM, Campos-Lopez AM, Figueiras A. Impact of a pharmacotherapeutic programme on control and safety of long-term anticoagulation treatment: a controlled follow-up study in Spain. Drug Saf. 2011;34(6):489-500.
60. Eckman MH, Lip GYH, Wise RE, Speer B, Sullivan M, Walker N, et al. Impact of an Atrial Fibrillation Decision Support Tool on thromboprophylaxis for atrial fibrillation. American Heart Journal. 2016;176:17-27.
61. Eitz T, Schenk S, Fritzsche D, Bairaktaris A, Wagner O, Koertke H, Koerfer R. International normalized ratio self-management lowers the risk of thromboembolic events after prosthetic heart valve replacement. Ann Thorac Surg. 2008;85(3):949-54; discussion 55.
62. Eldor A, Schwartz J. Self-management of oral anticoagulants with a whole blood prothrombin-time monitor in elderly patients with atrial fibrillation. Pathophysiol Haemost Thromb. 2002;32(3):99-106.
63. Falamic S, Lucijanic M, Hadziabdic MO, Marusic S, Bacic Vrca V. Pharmacist's interventions improve time in therapeutic range of elderly rural patients on warfarin therapy: a randomized trial. International Journal of Clinical Pharmacy. 2018;40(5):1078-85.
64. Falamic S, Lucijanic M, Ortner-Hadziabdic M, Marusic S, Bacic-Vrca V. Pharmacists' interventions improve health-related quality of life of rural older person on warfarin: a randomized controlled trial. Sci Rep. 2021;11(1):21897.*
65. Fariborz Farsad B, Dastan F, Salamzadeh J, Moghadamnia Z, Eskandari R, Fahimi F. Assessment of Outpatients' Knowledge and Adherence on Warfarin: The Impact of a Simple Educational Pamphlet. Iran J Pharm Res. 2019;18(Suppl1):315-20.
66. Feldeisen D, Alexandris-Souphis C, Haymart B, Gu X, Perry L, Watts S, et al. Higher OAK (Oral Anticoagulation Knowledge) score at baseline associated with better TTR (Time in Therapeutic Range) in patients taking warfarin. J Thromb Thrombolysis. 2023;55(1):141-8.
67. Finkelstein J, Khare R, Ansell J. Feasibility and patients' acceptance of Home Automated Telemanagement of oral anticoagulation therapy. AMIA Annu Symp Proc. 2003;Annual Symposium Proceedings/AMIA Symposium.:230-4.
68. Fitzmaurice DA, Hobbs FD, Murray ET, Holder RL, Allan TF, Rose PE. Oral anticoagulation management in primary care with the use of computerized decision support and near-patient testing: a randomized, controlled trial. Arch Intern Med. 2000;160(15):2343-8.
69. Fitzmaurice DA, Murray ET, Gee KM, Allan TF, Hobbs FD. A randomised controlled trial of patient self management of oral anticoagulation treatment compared with primary care management. J Clin Pathol. 2002;55(11):845-9.
70. Fitzmaurice DA, Murray ET, McCahon D, Holder R, Raftery JP, Hussain S, et al. Self management of oral anticoagulation: randomised trial. Bmj. 2005;331(7524):1057.
71. Franchi C, Antoniazzi S, Ardoino I, Proietti M, Marcucci M, Santalucia P, et al. Simulation-Based Education for Physicians to Increase Oral Anticoagulants in Hospitalized Elderly Patients with Atrial Fibrillation. Am J Med. 2019;132(8):e634-e47.
72. Gadisseur AP, Breukink-Engbers WG, van der Meer FJ, van den Besselaar AM, Sturk A, Rosendaal FR. Comparison of the quality of oral anticoagulant therapy through patient self-management and management by specialized anticoagulation clinics in the Netherlands: a randomized clinical trial. Arch Intern Med. 2003;163(21):2639-46.
73. Garcia CJ, Haynes K, Pokorney SD, Lin ND, McMahill-Walraven C, Nair V, et al. Practical challenges in the conduct of pragmatic trials embedded in health plans: Lessons of IMPACT-AFib, an FDA-Catalyst trial. Clin Trials. 2020;17(4):360-7.*
74. Gardiner C, Williams K, Longair I, Mackie IJ, Machin SJ, Cohen H. A randomised control trial of patient self-management of oral anticoagulation compared with patient self-testing. Br J Haematol. 2006;132(5):598-603.
75. Gardiner C, Longair I, Pescott MA, Erwin H, Hills J, Machin SJ, Cohen H. Self-monitoring of oral anticoagulation: does it work outside trial conditions? J Clin Pathol. 2009;62(2):168-71.
76. Gattellari M, Hayen A, Leung DYC, Zwar NA, Worthington JM. Supporting anticoagulant treatment decision making to optimise stroke prevention in complex patients with atrial fibrillation: a cluster randomised trial. BMC Fam Pract. 2020;21(1):102.
77. Gauci M, Wirth F, Azzopardi LM, Serracino-Inglott A. Clinical pharmacist implementation of a medication assessment tool for long-term management of atrial fibrillation in older persons. Pharm Pract (Granada). 2019;17(1):1349.
78. Grayson AD, Garnett F, Davies M, Connor N, Hughes C, Cooper JP. A consultant-led anticoagulation review of all patients in one clinical commissioning group to prevent atrial fibrillation related stroke. Int J Clin Pract. 2020;74(4):e13465.
79. Griffin BL, Burkiewicz JS, Peppers LR, Warholak TL. International Normalized Ratio values in group versus individual appointments in a pharmacist-managed anticoagulation clinic. Am J Health-Syst Pharm. 2009;66(13):1218-23.
80. Grunau BE, Wiens MO, Harder KK. Patient self-management of warfarin therapy: pragmatic feasibility study in Canadian primary care. Can Fam Physician. 2011;57(8):e292-8.
81. Gulpen AJW, Braeken DCW, Schalla S, Ten Cate H, Crijns HJ, Ten Cate-Hoek AJ. Long-Term Adherence to Direct Oral Anticoagulants in Patients with Atrial Fibrillation: A Comparative Cross-Sectional Study. Acta Haematol. 2022;145(5):476-83.
82. Guo Y, Chen Y, Lane DA, Liu L, Wang Y, Lip GYH. Mobile Health Technology for Atrial Fibrillation Management Integrating Decision Support, Education, and Patient Involvement: mAF App Trial. Am J Med. 2017;130(12):1388-96.e6.
83. Gutierrez JA, Christian RT, Aday AW, Gu L, Schulteis RD, Shihai L, et al. Electronic alerts to initiate anticoagulation dialogue in patients with atrial fibrillation. Am Heart J. 2022;245:29-40.
84. Hache J, Bonsu KO, Chitsike R, Nguyen H, Young S. Assessment of a Pharmacist-Led Direct Oral Anticoagulant Monitoring Clinic. Can J Hosp Pharm. 2021;74(1):7-14.
85. Harper P, Pollock D. Improved anticoagulant control in patients using home international normalized ratio testing and decision support provided through the Internet. Intern Med J. 2011;41(4):332-7.
86. Harrison J, Shaw JP, Harrison JE. Anticoagulation management by community pharmacists in New Zealand: an evaluation of a collaborative model in primary care. International Journal of Pharmacy Practice. 2015;23(3):173-81.
87. Hawes EM, Lambert E, Reid A, Tong G, Gwynne M. Implementation and evaluation of a pharmacist-led electronic visit program for diabetes and anticoagulation care in a patient-centered medical home. Am J Health-Syst Pharm. 2018;75(12):901-10.
88. Heaven B, Murtagh M, Rapley T, May C, Graham R, Kaner E, Thomson R. Patients or research subjects? A qualitative study of participation in a randomised controlled trial of a complex intervention. Patient Educ Couns. 2006;62(2):260-70.*
89. Heinrich K, Sanchez K, Hui C, Talabi K, Perry M, Qin H, et al. Impact of an electronic medium delivery of warfarin education in a low income, minority outpatient population: a pilot intervention study. BMC Public Health. 2019;19(1):1050.
90. Hendriks JM, de Wit R, Crijns HJ, Vrijhoef HJ, Prins MH, Pisters R, et al. Nurse-led care vs. usual care for patients with atrial fibrillation: results of a randomized trial of integrated chronic care vs. routine clinical care in ambulatory patients with atrial fibrillation. European Heart Journal. 2012;33(21):2692-9.
91. Holm T, Lassen JF, Husted SE, Christensen P, Heickendorff L. A randomized controlled trial of shared care versus routine care for patients receiving oral anticoagulant therapy. J Intern Med. 2002;252(4):322-31.
92. Holt TA, Dalton A, Marshall T, Fay M, Qureshi N, Kirkpatrick S, et al. Automated Software System to Promote Anticoagulation and Reduce Stroke Risk: Cluster-Randomized Controlled Trial. Stroke. 2017;48(3):787-90.
93. Holt TA, Kirkpatrick S, Hislop J, Kearley K, Mollison J, Yu LM, et al. Barriers to a software reminder system for risk assessment of stroke in atrial fibrillation: A process evaluation of a cluster randomised trial in general practice. British Journal of General Practice. 2018;68(677):E844-E51.*
94. Hua TD, Vormfelde SV, Abu Abed M, Schneider-Rudt H, Sobotta P, Friede T, Chenot JF. Practice nursed-based, individual and video-assisted patient education in oral anticoagulation--protocol of a cluster-randomized controlled trial. BMC Fam Pract. 2011;12:17.*
95. Hutchinson-Jones NW, Didcott SK, Jones MD, Crowe JN. Implementation of a standardised annual anticoagulation specialist review in primary care. Prim Health Care Res Dev. 2020;21:e17.
96. Inglis S, McLennan S, Dawson A, Birchmore L, Horowitz JD, Wilkinson D, Stewart S. A new solution for an old problem? Effects of a nurse-led, multidisciplinary, home-based intervention on readmission and mortality in patients with chronic atrial fibrillation. J Cardiovasc Nurs. 2004;19(2):118-27.
97. Jackson SL, Peterson GM, Bereznicki LR, Misan GM, Jupe DM, Vial JH. Improving the outcomes of anticoagulation in rural Australia: an evaluation of pharmacist-assisted monitoring of warfarin therapy. J Clin Pharm Ther. 2005;30(4):345-53.
98. Jenner KM, Simmons BJ, Delate T, Clark NP, Kurz D, Witt DM. An Education Program for Patient Self-Management of Warfarin. Perm. 2015;19(4):33-8.*
99. Jiang S, He Q, Yan J, Zhao L, Zheng Y, Chen P, Chen X. Evaluation of a Pharmacist-Led Remote Warfarin Management Model Using a Smartphone Application (Yixing) in Improving Patients' Knowledge and Outcomes of Anticoagulation Therapy. Front Pharmacol. 2021;12:677943.
100. Jiang S, Lv M, Zeng Z, Fang Z, Chen M, Qian J, et al. Efficacy and safety of app-based remote warfarin management during COVID-19-related lockdown: a retrospective cohort study. J Thromb Thrombolysis. 2022;54(1):20-8.
101. Kallal A, Griffen D, Jaeger C. Using Lean Six Sigma methodologies to reduce risk of warfarin medication omission at hospital discharge. BMJ Open Qual. 2020;9(2).
102. Kapoor A, Amroze A, Golden J, Crawford S, O'Day K, Elhag R, et al. SUPPORT-AF: Piloting a Multi-Faceted, Electronic Medical Record-Based Intervention to Improve Prescription of Anticoagulation. J Am Heart Assoc. 2018;7(17):e009946.
103. Kapoor A, Amroze A, Vakil F, Crawford S, Der J, Mathew J, et al. SUPPORT-AF II: Supporting Use of Anticoagulants Through Provider Profiling of Oral Anticoagulant Therapy for Atrial Fibrillation: A Cluster-Randomized Study of Electronic Profiling and Messaging Combined With Academic Detailing for Providers Making Decisions About Anticoagulation in Patients With Atrial Fibrillation. Circ Cardiovasc Qual Outcomes. 2020;13(2):e005871.
104. Karaoui LR, Ramia E, Mansour H, Haddad N, Chamoun N. Impact of pharmacist-conducted anticoagulation patient education and telephone follow-up on transitions of care: a randomized controlled trial. BMC Health Serv Res. 2021;21(1):151.
105. Karlsson LO, Nilsson S, Bang M, Nilsson L, Charitakis E, Janzon M. A clinical decision support tool for improving adherence to guidelines on anticoagulant therapy in patients with atrial fibrillation at risk of stroke: A cluster-randomized trial in a Swedish primary care setting (the CDS-AF study). PLoS Med. 2018;15(3):e1002528.
106. Koertke H, Zittermann A, Wagner O, Koerfer R. Self-management of oral anticoagulation therapy improves long-term survival in patients with mechanical heart valve replacement. Ann Thorac Surg. 2007;83(1):24-9.*
107. Khalil V. Redesign of computerized decision support system to improve Non Vitamin K oral anticoagulant prescribing-A pre and post qualitative and quantitative study. Int J Med Inform. 2021;152:104511.
108. Khalil V, Blackley S, Subramaniam A. Evaluation of a pharmacist-led shared decision-making in atrial fibrillation and patients' satisfaction-a before and after pilot study. Ir J Med Sci. 2021;190(2):819-24.
109. Khan TI, Kamali F, Kesteven P, Avery P, Wynne H. The value of education and self-monitoring in the management of warfarin therapy in older patients with unstable control of anticoagulation. Br J Haematol. 2004;126(4):557-64.
110. Kimmel SE, Troxel AB, Loewenstein G, Brensinger CM, Jaskowiak J, Doshi JA, et al. Randomized trial of lottery-based incentives to improve warfarin adherence. American Heart Journal. 2012;164(2):268-74.
111. Koplan KE, Brush AD, Packer MS, Zhang F, Senese MD, Simon SR. "Stealth" alerts to improve warfarin monitoring when initiating interacting medications. Journal of General Internal Medicine. 2012;27(12):1666-73.
112. Kortke H, Korfer R. International normalized ratio self-management after mechanical heart valve replacement: is an early start advantageous? Ann Thorac Surg. 2001;72(1):44-8.
113. Labovitz DL, Shafner L, Reyes Gil M, Virmani D, Hanina A. Using Artificial Intelligence to Reduce the Risk of Nonadherence in Patients on Anticoagulation Therapy. Stroke. 2017;48(5):1416-9.
114. Lakshmi R, James E, Kirthivasan R. Study on impact of clinical pharmacist's interventions in the optimal use of oral anticoagulants in stroke patients. Indian Journal of Pharmaceutical Sciences. 2013;75(1):53-9.
115. Lalonde L, Martineau J, Blais N, Montigny M, Ginsberg J, Fournier M, et al. Is long-term pharmacist-managed anticoagulation service efficient? A pragmatic randomized controlled trial. American Heart Journal. 2008;156(1):148-54.
116. Lee JA, Evangelista LS, Moore AA, Juth V, Guo Y, Gago-Masague S, et al. Feasibility Study of a Mobile Health Intervention for Older Adults on Oral Anticoagulation Therapy. Gerontology and Geriatric Medicine. 2016;2(no pagination).
117. Lee LC, Farwig P, Kirk L, Mitchell VD, Sabatino JA, Barnes KD. Impact of pharmacist intervention on anticoagulation management and risk for potential COVID-19 exposure during the COVID-19 pandemic. Thromb Res. 2022;217:52-6.
118. Lee SY, Cherian R, Ly I, Horton C, Salley AL, Sarkar U. Designing and Implementing an Electronic Patient Registry to Improve Warfarin Monitoring in the Ambulatory Setting. Joint Commission Journal on Quality and Patient Safety. 2017;43(7):353-60.
119. Li Y, Dong L, Xiang D, Zhang Y, Chen X, Long J, et al. Patient compliance with an anticoagulation management system based on a smartphone application. J Thromb Thrombolysis. 2019;48(2):263-9.
120. Liang JB, Lao CK, Tian L, Yang YY, Wu HM, Tong HH, Chan A. Impact of a pharmacist-led education and follow-up service on anticoagulation control and safety outcomes at a tertiary hospital in China: a randomised controlled trial. Int J Pharm Pract. 2020;28(1):97-106.
121. Lin SW, Kang WY, Lin DT, Lee J, Wu FL, Chen CL, Tseng YJ. Comparison of warfarin therapy clinical outcomes following implementation of an automated mobile phone-based critical laboratory value text alert system. BMC Med Genomics. 2014;7 Suppl 1:S13.
122. Lin SY, Chen YW, Kang HC, Wu YJ, Chen PZ, Wu CW, et al. Effects of a pharmacist-managed anticoagulation outpatient clinic in Taiwan: evaluation of patient knowledge, satisfaction, and clinical outcomes. Postgrad Med. 2021;133(8):964-73.
123. Loewen PS, Bansback N, Hicklin J, Andrade JG, Kapanen AI, Kwan L, et al. Evaluating the Effect of a Patient Decision Aid for Atrial Fibrillation Stroke Prevention Therapy. Annals of Pharmacotherapy. 2019.
124. Machtinger EL, Wang F, Chen LL, Rodriguez M, Wu S, Schillinger D. A visual medication schedule to improve anticoagulation control: a randomized, controlled trial. Jt Comm J Qual Patient Saf. 2007;33(10):625-35.
125. Maikranz V, Siebenhofer A, Ulrich LR, Mergenthal K, Schulz-Rothe S, Kemperdick B, et al. Does a complex intervention increase patient knowledge about oral anticoagulation? - a cluster-randomised controlled trial. BMC Fam Pract. 2017;18(1):15.
126. Manotti C, Moia M, Palareti G, Pengo V, Ria L, Dettori AG. Effect of computer-aided management on the quality of treatment in anticoagulated patients: a prospective, randomized, multicenter trial of APROAT (Automated PRogram for Oral Anticoagulant Treatment). Haematologica. 2001;86(10):1060-70.
127. Marcatto L, Boer B, Sacilotto L, Olivetti N, Darrieux FCC, Scanavacca MI, et al. Impact of adherence to warfarin therapy during 12 weeks of pharmaceutical care in patients with poor time in the therapeutic range. J Thromb Thrombolysis. 2021;51(4):1043-9.
128. Marco F, Sedano C, Bermudez A, Lopez-Duarte M, Fernandez-Fontecha E, Zubizarreta A. A prospective controlled study of a computer-assisted acenocoumarol dosage program. Pathophysiol Haemost Thromb. 2003;33(2):59-63.
129. Margolis A, Nagy M, Schoen R, Porter A. Provider comfort and confidence with an extended INR follow-up interval for veteran patients on stable doses of warfarin. American Journal of Hematology. 2018;93 (9):E44.*
130. Margolis AR, Porter AL, Staresinic CE, Ray CA. Impact of an extended International Normalized Ratio follow-up interval on healthcare use among veteran patients on stable warfarin doses. Am J Health Syst Pharm. 2019;76(22):1848-52.*
131. Marquez-Contreras E, Martell-Claros N, Marquez-Rivero S, Hermida-Campa E, Gracia-Diez C, Sanchez-Lopez E, Gil-Guillen V. Strategies for improving dabigatran adherence for stroke prevention in patients with non-valvular atrial fibrillation: education and drug intake reminders (FACILITA study). Current Medical Research and Opinion. 2018;34(7):1301-8
132. Matchar DB, Samsa GP, Cohen SJ, Oddone EZ, Jurgelski AE. Improving the quality of anticoagulation of patients with atrial fibrillation in managed care organizations: results of the managing anticoagulation services trial. Am J Med. 2002;113(1):42-51.
133. Matchar DB. Do anticoagulation management services improve care? Implications of the Managing Anticoagulation Services Trial. Card Electrophysiol Rev. 2003;7(4):379-81.*
134. Matchar DB, Dolor R, Jacobson A, Love S, Edson R, Uyeda L. More Frequent Self-Testing of Prothrombin Time Results in Improved Time in Target Range. Circulation Conference: American Heart Association. 2012;126(21 SUPPL. 1).*
135. Matchar DB, Love SR, Jacobson AK, Edson R, Uyeda L, Phibbs CS, Dolor RJ. The impact of frequency of patient self-testing of prothrombin time on time in target range within VA Cooperative Study #481: The Home INR Study (THINRS), a randomized, controlled trial. J Thromb Thrombolysis. 2015;40(1):17-25.
136. Mavri A, Ostasevski Fernandez N, Kramaric A, Kosmelj K. New educational approach for patients on warfarin improves knowledge and therapy control. Wien Klin Wochenschr. 2015;127(11-12):472-6.
137. Mazor KM, Baril J, Dugan E, Spencer F, Burgwinkle P, Gurwitz JH. Patient education about anticoagulant medication: is narrative evidence or statistical evidence more effective? Patient Educ Couns. 2007;69(1-3):145-57.
138. McAlister FA, Man-Son-Hing M, Straus SE, Ghali WA, Gibson P, Anderson D, et al. A randomized trial to assess the impact of an antithrombotic decision aid in patients with nonvalvular atrial fibrillation: the DAAFI trial protocol [ISRCTN14429643]. BMC Cardiovascular Disorders. 2004;4:5.
139. McNab D, McKay J, Bowie P. A before and after study of warfarin monitoring in a single region as part of the Scottish patient safety programme in primary care. Scott Med J. 2015;60(4):196-201.
140. Menendez-Jandula B, Souto JC, Oliver A, Montserrat I, Quintana M, Gich I, et al. Comparing self-management of oral anticoagulant therapy with clinic management: a randomized trial. Ann Intern Med. 2005;142(1):1-10.
141. Mertens BJ, Kwint HF, Belitser SV, van der Meer FJM, van Marum RJ, Bouvy ML. Effect of multidose drug dispensing on the time in therapeutic range in patients using vitamin-K antagonists: A randomized controlled trial. J Thromb Haemost. 2020;18(1):70-8.
142. Mertens C, Siebenhofer A, Berghold A, Pregartner G, Ulrich L-R, Mergenthal K, et al. Differences in the quality of oral anticoagulation therapy with vitamin K antagonists in German GP practices - results of the cluster-randomized PICANT trial (Primary Care Management for Optimized Antithrombotic Treatment). BMC Health Serv Res. 2019;19(1):539.*
143. Mifsud EM, Wirth F, Serracino-Inglott A, Azzopardi LM. Pharmacist-led medicine use review for patients on anticoagulation therapy. International Journal of Clinical Pharmacy. 2018;40 (1):213-4.
144. Miller RJH, Chew DS, Rezazadeh S, Klassen S, Pournazari P, Lang E, Quinn FR. Factors Influencing Oral Anticoagulation Prescription for Patients Presenting to Emergency Departments With Atrial Fibrillation and Flutter. The Canadian journal of cardiology. 2018;34(6):804-7.*
145. Moloney E, Craig D, Holdsworth N, Smithson J. Optimising clinical effectiveness and quality along the atrial fibrillation anticoagulation pathway: an economic analysis. BMC Health Serv Res. 2019;19(1):1007.
146. Montalescot G, Brotons C, Cosyns B, Crijns HJ, D'Angelo A, Drouet L, et al. Educational Impact on Apixaban Adherence in Atrial Fibrillation (the AEGEAN STUDY): A Randomized Clinical Trial. Am J Cardiovasc Drugs. 2020;20(1):61-71.
147. Moore SJ, Blair EA, Steeb DR, Reed BN, Hull JH, Rodgers JE. Impact of video technology on efficiency of pharmacist-provided anticoagulation counseling and patient comprehension. Ann Pharmacother. 2015;49(6):631-8.
148. Moraes RM, Winkelmann ER, Colet CdF. Use of a mobile application for the control of anticoagulation with warfarin: a cluster randomized controlled clinical trial - Ijui/RS/Brazil. Brazilian Journal of Pharmaceutical Sciences. 2022;58.
149. Moss RC, Lowe GC, Frampton CA, Revell P. A nurse-led randomised controlled trial of a structured educational programme for patients starting warfarin therapy. Journal of Research in Nursing. 2014;19(5):402-12.
150. Navin SF, Nardolillo J, Stambaugh A, Young C, Nguyen P, Apodaca M. Pharmacist monitoring of direct oral anticoagulants for American Indians and Alaska Natives in the outpatient setting. J Am Pharm Assoc (2003). 2022;62(2):598-603.
151. Nguyen VN, Stevens CA, Brambatti M, Smith M, Braun OO, Mariski M, Jr., et al. Improved Time in Therapeutic Range with International Normalized Ratio Remote Monitoring for Patients with Left Ventricular Assist Devices. ASAIO J. 2022;68(3):363-8.
152. Nieuwlaat R, Eikelboom JW, Schulman S, van Spall HG, Schulze KM, Connolly BJ, et al. Cluster randomized controlled trial of a simple warfarin maintenance dosing algorithm versus usual care among primary care practices. J Thromb Thrombolysis. 2014;37(4):435-42.
153. Nochowitz B, Shapiro NL, Nutescu EA, Cavallari LH. Effect of a warfarin adherence aid on anticoagulation control in an inner-city anticoagulation clinic population. Ann Pharmacother. 2009;43(7):1165-72.
154. O'Shea SI, Arcasoy MO, Samsa G, Cummings SE, Thames EH, Surwit RS, Ortel TL. Direct-to-patient expert system and home INR monitoring improves control of oral anticoagulation. J Thromb Thrombolysis. 2008;26(1):14-21.
155. Ono F, Akiyama S, Suzuki A, Ikeda Y, Takahashi A, Matsuoka H, et al. Impact of care coordination on oral anticoagulant therapy among patients with atrial fibrillation in routine clinical practice in Japan: a prospective, observational study. BMC Cardiovasc Disord. 2019;19(1):235.
156. Orchard J, Neubeck L, Freedman B, Li J, Webster R, Zwar N, et al. eHealth Tools to Provide Structured Assistance for Atrial Fibrillation Screening, Management, and Guideline-Recommended Therapy in Metropolitan General Practice: The AF - SMART Study. J Am Heart Assoc. 2019;8(1):e010959.
157. Parkash R, Magee K, McMullen M, Clory M, D'Astous M, Robichaud M, et al. The Canadian Community Utilization of Stroke Prevention Study in Atrial Fibrillation in the Emergency Department (C-CUSP ED). Annals of Emergency Medicine. 2019;73(4):382-92.
158. Patsiou V, Samaras A, Kartas A, Moysidis DV, Papazoglou AS, Bekiaridou A, et al. Prognostic implications of adherence to oral anticoagulants among patients with atrial fibrillation: Insights from MISOAC-AF trial. J Cardiol. 2023;81(4):390-6.*
159. Piazza G, Hurwitz S, Campia U, Bikdeli B, Lou J, Khairani CD, et al. Electronic alerts for ambulatory patients with atrial fibrillation not prescribed anticoagulation: A randomized, controlled trial (AF-ALERT2). Thromb Res. 2023;227:1-7.
160. Pokorney SD, Cocoros N, Al-Khalidi HR, Haynes K, Li S, Al-Khatib SM, et al. Effect of Mailing Educational Material to Patients With Atrial Fibrillation and Their Clinicians on Use of Oral Anticoagulants: A Randomized Clinical Trial. JAMA Netw Open. 2022;5(5):e2214321.
161. Porter AL, Margolis AR, Schoen RR, Staresinic CE, Ray CA, Fletcher CD. Use of an extended INR follow-up interval for Veteran patients in an anticoagulation clinic. J Thromb Thrombolysis. 2017;43(3):318-25.*
162. Porter AL, Margolis AR, Staresinic CE, Nagy MW, Schoen RR, Ray CA, Fletcher CD. Feasibility and safety of a 12-week INR follow-up protocol over 2 years in an anticoagulation clinic: a single-arm prospective cohort study. Journal of Thrombosis and Thrombolysis. 2019;47(2):200-8.
163. Price EL, Ansell J. Virtual Education for Patient Self-Testing for Warfarin Therapy Is Effective During the COVID-19 Pandemic. Jt Comm J Qual Patient Saf. 2022;48(4):214-21.
164. Price V, Collins M, Eordogh A, Oatley H, Shapiro S. Anticoagulation: supporting safe and optimal primary care prescribing in a rapidly changing field. Br J Haematol. 2019;186(3):e17-e20.
165. Putriana NA, Lestari K, Barliana MI, Hartini S. Effects of pharmacist counseling on compliance and international normalized ratio score on outpatients receiving warfarin at dr. Hasan sadikin bandung hospital west java, Indonesia. Asian Journal of Pharmaceutical and Clinical Research. 2017;10(Special Issue may):72-5.
166. Rao MP, Ciobanu AO, Lopes RD, Fox KA, Xian Y, Pokorney SD, et al. A clustered randomized trial to IMProve treatment with AntiCoagulanTs in patients with Atrial Fibrillation (IMPACT-AF): Design and rationale. American Heart Journal. 2016;176:107-13.*
167. Rapley T, May C, Heaven B, Murtagh M, Graham R, Kaner EFS, Thomson R. Doctor-patient interaction in a randomised controlled trial of decision-support tools. Social science & medicine (1982). 2006;62(9):2267-78.*
168. Reverdin S, Schnetzler B, Gagneux G, Gavignet C, Boehlen F, De Moerloose P. Implementation of an INR self-testing and self-management programme in common ambulatory private practice: Our experience with 90 patients. Swiss Medical Weekly. 2011;141(MAY).
169. Rezazadeh S, Chew DS, Miller RJH, Klassen S, Pournazari P, Bennett G, et al. Effects of a reminder to initiate oral anticoagulation in patients with atrial fibrillation/atrial flutter discharged from the emergency department: REMINDER study. Cjem. 2018;20(6):841-9.
170. Rivera-Caravaca JM, Gil-Perez P, Lopez-Garcia C, Veliz-Martinez A, Quintana-Giner M, Romero-Aniorte AI, et al. A nurse-led atrial fibrillation clinic: Impact on anticoagulation therapy and clinical outcomes. Int J Clin Pract. 2020;74(12):e13634.
171. Romanelli S, Rivera V. Converting patients from warfarin to non-vitamin K antagonist oral anticoagulants. J Am Assoc Nurse Pract. 2023;35(6):380-5.
172. Romano T, Rein L, Celebre G, Hardman J. Comparing therapy outcomes of patients before and after enrolling in an anticoagulation self‐testing program. Jaccp: Journal of the American College of Clinical Pharmacy. 2020;3(4):743-8.
173. Rose AE, Robinson EN, Premo JA, Hauschild LJ, Trapskin PJ, McBride AM. Improving Warfarin Management Within the Medical Home: A Health-System Approach. Am J Med. 2017;130(3):365.e7-.e12.
174. Roseau C, Richard C, Renet S, Kowal C, Eliahou L, Rieutord A, Chaumais MC. Evaluation of a program of pharmaceutical counseling for French patients on oral anticoagulant therapy. Int J Clin Pharm. 2020;42(2):685-94.
175. Rossier C, Spoutz P, Schaefer M, Allen A, Patterson ME. Working smarter, not harder: evaluating a population health approach to anticoagulation therapy management. J Thromb Thrombolysis. 2021;52(1):200-8.
176. Ru X, Wang T, Zhu L, Ma Y, Qian L, Sun H, Pan Z. Using a Clinical Decision Support System to Improve Anticoagulation in Patients with Nonvalve Atrial Fibrillation in China's Primary Care Settings: A Feasibility Study. Int J Clin Pract. 2023;2023:2136922.
177. Ryan F, Byrne S, O'Shea S. Randomized controlled trial of supervised patient self-testing of warfarin therapy using an internet-based expert system. J Thromb Haemost. 2009;7(8):1284-90.
178. Salvador CH, Ruiz-Sanchez A, Gonzalez de Mingo MA, Carmona Rodriguez M, Carrasco MP, Sagredo PG, et al. Evaluation of a telemedicine-based service for the follow-up and monitoring of patients treated with oral anticoagulant therapy. IEEE Trans Inf Technol Biomed. 2008;12(6):696-706.
179. Saokaew S, Sapoo U, Nathisuwan S, Chaiyakunapruk N, Permsuwan U. Anticoagulation control of pharmacist-managed collaborative care versus usual care in Thailand. International Journal of Clinical Pharmacy. 2012;34(1):105-12.
180. Saw Y, Yap SY, Tan YH. Evaluation of the clinical and safety outcomes of face‐to‐face vs a telephonic model of a pharmacist‐led outpatient anticoagulation service. Jaccp: Journal of the American College of Clinical Pharmacy. 2020;3(8):1444-50.
181. Schillig J, Kaatz S, Hudson M, Krol GD, Szandzik EG, Kalus JS. Clinical and safety impact of an inpatient pharmacist-directed anticoagulation service. J Hosp Med. 2011;6(6):322-8.
182. Schlemmer T. An anticoagulation safety program in home care: identifying areas for improvement with an educational program for registered nurses and physical therapists. Home Healthc Nurse. 2011;29(1):13-20.
183. Schoen RR, Nagy MW, Porter AL, Margolis AR. Patient Satisfaction With Extended International Normalized Ratio Follow-up Intervals in a Veteran Population. The Annals of pharmacotherapy. 2020;54(5):442-9.*
184. Schupbach RR, Bousum JM, Miller MJ. Demonstration of anticoagulation patient self-testing feasibility at an Indian Health Service facility: A case series analysis. Pharmacy Practice. 2013;11(1):30-7.
185. Sheibani R, Sheibani M, Heidari-Bakavoli A, Abu-Hanna A, Eslami S. The Effect of a Clinical Decision Support System on Improving Adherence to Guideline in the Treatment of Atrial Fibrillation: An Interrupted Time Series Study. Journal of Medical Systems. 2018;42 (2) (no pagination)(26).
186. Shiga T, Kimura T, Fukushima N, Yoshiyama Y, Iwade K, Mori F, et al. Effects of a Pharmacist-Led Educational Interventional Program on Electronic Monitoring-assessed Adherence to Direct Oral Anticoagulants: A Randomized, Controlled Trial in Patients with Nonvalvular Atrial Fibrillation. Clin Ther. 2022;44(11):1494-505.
187. Shilbayeh SAR. The Impact of a Pharmacist-led Warfarin Educational Video in a Saudi Setting. J Pharm Bioallied Sci. 2020;12(4):413-22.
188. Sidhu P, O'Kane HO. Self-managed anticoagulation: results from a two-year prospective randomized trial with heart valve patients. Ann Thorac Surg. 2001;72(5):1523-7.
189. Siebenhofer A, Rakovac I, Kleespies C, Piso B, Didjurgeit U. Self-management of oral anticoagulation in the elderly: rationale, design, baselines and oral anticoagulation control after one year of follow-up. A randomized controlled trial. Thromb Haemost. 2007;97(3):408-16.
190. Siebenhofer A, Rakovac I, Kleespies C, Piso B, Didjurgeit U. Self-management of oral anticoagulation reduces major outcomes in the elderly a randomized controlled trial. Thrombosis and Haemostasis. 2008;100(6):1089-98.*
191. Siebenhofer A, Ulrich LR, Mergenthal K, Roehl I, Rauck S, Berghold A, et al. Primary care management for optimized antithrombotic treatment [PICANT]: study protocol for a cluster-randomized controlled trial. Implement Sci. 2012;7:79.*
192. Siebenhofer A, Ulrich LR, Mergenthal K, Berghold A, Pregartner G, Kemperdick B, et al. Primary care management for patients receiving long-term antithrombotic treatment: A cluster-randomized controlled trial. PLoS ONE. 2019;14 (1) (no pagination)(e0209366).*
193. Silbernagel G, Spirk D, Hager A, Baumgartner I, Kucher N. Electronic Alert System for Improving Stroke Prevention Among Hospitalized Oral-Anticoagulation-Naive Patients With Atrial Fibrillation: A Randomized Trial. J Am Heart Assoc. 2016;5(7):22.
194. Simmons BJ, Jenner KM, Delate T, Clark NP, Kurz D, Witt DM. Pilot study of a novel patient self-management program for warfarin therapy using venipuncture-acquired international normalized ratio monitoring. Pharmacotherapy. 2012;32(12):1078-84.
195. Singh LG, Accursi M, Korch Black K. Implementation and outcomes of a pharmacist-managed clinical video telehealth anticoagulation clinic. Am J Health-Syst Pharm. 2015;72(1):70-3.
196. Skolarus LE, Morgenstern LB, Scott PA, Lisabeth LD, Murphy JB, Migda EM, Brown DL. An emergency department intervention to increase warfarin use for atrial fibrillation. J Stroke Cerebrovasc Dis. 2014;23(2):199-203.
197. Soliman Hamad MA, van Eekelen E, van Agt T, van Straten AH. Self-management program improves anticoagulation control and quality of life: a prospective randomized study. Eur J Cardiothorac Surg. 2009;35(2):265-9.
198. Solvik UO, Lokkebo E, Kristoffersen AH, Brodin E, Averina M, Sandberg S. Quality of Warfarin Therapy and Quality of Life are Improved by Self-Management for Two Years. Thromb Haemost. 2019;119(10):1632-41.
199. Stafford L, Peterson GM, Bereznicki LR, Jackson SL, Van Tienen EC. Training Australian pharmacists for participation in a collaborative, home-based post-discharge warfarin management service. Pharmacy World and Science. 2010;32(5):637-42.*
200. Stafford L, Peterson GM, Bereznicki LR, Jackson SL, van Tienen EC, Angley MT, et al. Clinical outcomes of a collaborative, home-based postdischarge warfarin management service. Ann Pharmacother. 2011;45(3):325-34.
201. Stafford L, van Tienen EC, Bereznicki LR, Peterson GM. The benefits of pharmacist-delivered warfarin education in the home. International Journal of Pharmacy Practice. 2012;20(6):384-9.*
202. Sun J, Chen GM, Huang J. Effect of Evidence-Based Pharmacy Care on Satisfaction and Cognition in Patients with Non-Valvular Atrial Fibrillation Taking Rivaroxaban. Patient Prefer Adherence. 2021;15:1661-70.
203. Swarna Nantha Y. Anticoagulant management of atrial fibrillation: the influence of dosing algorithm and recall schedule on time in therapeutic range. Fam Pract. 2015;32(5):514-9.
204. Taborski U, Voller H, Kortke H, Blunt J, Wegscheider K. Self Management of Oral Anticoagulation with the INRatio System: Accuracy and Reliability Following a Two-Day Structured Training Program. Laboratory Medicine. 2004;35(5):303-7.
205. Talboom-Kamp E, Verdijk NA, Kasteleyn MJ, Harmans LM, Talboom I, Numans ME, Chavannes NH. Effect of a combined education and eHealth programme on the control of oral anticoagulation patients (PORTALS study): a parallel cohort design in Dutch primary care. BMJ Open. 2017;7(9):e017909.
206. Talboom-Kamp EP, Verdijk NA, Talboom IJ, Harmans LM, Numans ME, Chavannes NH. PORTALS: design of an innovative approach to anticoagulation management through eHealth. BMC Health Serv Res. 2017;17(1):213.*
207. Tamayo Aguirre E, Galo-Anza A, Dorronsoro-Barandiaran O, Del Burgo EU-S, Ostiza Irigoyen A, Garcia-Carro A, et al. Oral anticoagulation with vitamin K inhibitors and determinants of successful self-management in primary care. BMC cardiovascular disorders. 2016;16(1):180.
208. Tang T, Zhang X, Tao L, Sun A, Qiu S, Zhang F, et al. Impact of the disease management model of "treatment-education-follow-up" on anticoagulant therapy in patients with stroke and atrial fibrillation. Biomedical Research (India). 2017;28(16):7195-201.
209. Thomson RG, Eccles MP, Steen IN, Greenaway J, Stobbart L, Murtagh MJ, May CR. A patient decision aid to support shared decision-making on anti-thrombotic treatment of patients with atrial fibrillation: randomised controlled trial. Qual Saf Health Care. 2007;16(3):216-23.
210. Toscos T, Coupe A, Wagner S, Ahmed R, Roebuck A, Flanagan M, et al. Engaging Patients in Atrial Fibrillation Management via Digital Health Technology: The Impact of Tailored Messaging. J Innov Card Rhythm Manag. 2020;11(8):4209-17.
211. Touchette DR, McGuinness ME, Stoner S, Shute D, Edwards JM, Ketchum K. Improving outpatient warfarin use for hospitalized patients with atrial fibrillation. Pharmacy Practice. 2008;6(1):43-50.
212. Tran AT, Okasha OM, Steinhaus DA, Yousuf OK, Giocondo MJ, Ramza BM, et al. Prospective evaluation of the effect of smartphone electrocardiogram usage on anticoagulant medication compliance. J Interv Card Electrophysiol. 2022;65(2):453-60.
213. Tran HN, Tafreshi J, Hernandez EA, Pai SM, Torres VI, Pai RG. A multidisciplinary atrial fibrillation clinic. Curr Cardiol Rev. 2013;9(1):55-62.
214. Triller D, Wymer S, Morris K, Farman G, Myrka A. Improving warfarin safety in long-term care. Consultant Pharmacist. 2014;29(7):453-68.
215. Turakhia M, Sundaram V, Smith SN, Ding V, Michael Ho P, Kowey PR, et al. Efficacy of a centralized, blended electronic, and human intervention to improve direct oral anticoagulant adherence: Smartphones to improve rivaroxaban ADHEREnce in atrial fibrillation (SmartADHERE) a randomized clinical trial. Am Heart J. 2021;237:68-78.
216. Tyedin AE, Taylor SE, Than J, Al‐Alawi R, O’Halloran E, Chau AH. Impact of proactive pharmacist‐assisted warfarin management using an electronic medication management system in Australian hospitalised patients. Journal of Pharmacy Practice and Research. 2020;50(2):144-51.
217. Tzikas A, Samaras A, Kartas A, Vasdeki D, Fotos G, Dividis G, et al. Motivational Interviewing to Support Oral AntiCoagulation adherence in patients with non-valvular Atrial Fibrillation (MISOAC-AF): a randomized clinical trial. Eur Heart J Cardiovasc Pharmacother. 2021;7(FI1):f63-f71.
218. Ulrich LR, Mergenthal K, Petersen JJ, Roehl I, Rauck S, Kemperdick B, et al. Anticoagulant treatment in German family practices - screening results from a cluster randomized controlled trial. BMC Fam Pract. 2014;15:170.*
219. Valencia D, Spoutz P, Stoppi J, Kibert JL, 2nd, Allen A, Parra D, Hough A. Impact of a Direct Oral Anticoagulant Population Management Tool on Anticoagulation Therapy Monitoring in Clinical Practice. Ann Pharmacother. 2019;53(8):806-11.
220. Vall Vargas A, Altes Hernandez A, Arnau A, Constans Cots M, Gallart Fernandez-Puebla A, de Juan Pardo MA. Effectiveness of a self-management programme in the treatment of antivitamin K oral anticoagulants. A feasibility study. Med Clin (Barc). 2020;154(10):388-93.
221. Van Beek A, Bowers M, Hall B, Meyer B. Implementation of home-based international normalized ratio testing in adult patients treated with warfarin. J Am Assoc Nurse Pract. 2019;33(7):563-9.
222. Verret L, Couturier J, Rozon A, Saudrais-Janecek S, St-Onge A, Nguyen A, et al. Impact of a pharmacist-led warfarin self-management program on quality of life and anticoagulation control: a randomized trial. Pharmacotherapy. 2012;32(10):871-9.
223. Vinereanu D, Lopes RD, Bahit MC, Xavier D, Jiang J, Al-Khalidi HR, et al. A multifaceted intervention to improve treatment with oral anticoagulants in atrial fibrillation (IMPACT-AF): an international, cluster-randomised trial. Lancet. 2017;390(10104):1737-46.
224. Vinereanu D, Al-Khalidi HR, Rao MP, He W, Lopes RD, Bahit CM, et al. Regional differences in presentation and antithrombotic treatment of patients with atrial fibrillation: Baseline characteristics from a clustered randomized trial to IMProve treatment with AntiCoagulanTs in patients with atrial fibrillation (IMPACT-AF). American Heart Journal. 2017;192:38-47.*
225. Viquez-Jaikel A, Victoria H-R, Ramos-Esquivel A. Improvement of time in therapeutic range with warfarin by pharmaceutical intervention. International Journal of Clinical Pharmacy. 2017;39(1):41-4.
226. Virdee MS, Stewart D. Optimizing the use of oral anticoagulant therapy for atrial fibrilation in primary care: a pharmacist-led intervention. International Journal of Clinical Pharmacy. 2017;39(1):173-80.
227. Volpp KG, Loewenstein G, Troxel AB, Doshi J, Price M, Laskin M, Kimmel SE. A test of financial incentives to improve warfarin adherence. BMC Health Serv Res. 2008;8 (no pagination)(272).
228. Vormfelde SV, Abu Abed M, Hua TD, Schneider S, Friede T, Chenot JF. Educating orally anticoagulated patients in drug safety: a cluster-randomized study in general practice. Dtsch. 2014;111(37):607-14.
229. Wang N, Qiu S, Yang Y, Zhang C, Gu ZC, Qian Y. Physician-Pharmacist Collaborative Clinic Model to Improve Anticoagulation Quality in Atrial Fibrillation Patients Receiving Warfarin: An Analysis of Time in Therapeutic Range and a Nomogram Development. Front Pharmacol. 2021;12:673302.
230. Wang PJ, Lu Y, Mahaffey KW, Lin A, Morin DP, Sears SF, et al. Randomized Clinical Trial to Evaluate an Atrial Fibrillation Stroke Prevention Shared Decision-Making Pathway. J Am Heart Assoc. 2023;12(3):e028562.
231. Wang SV, Rogers JR, Jin Y, Decicicchi D, DeJene SZ, Connors JM, et al. Intervention to increase appropriate anticoagulation in patients with atrial fibrillation. Pharmacoepidemiology and Drug Safety. 2017;26 (Supplement 2):10
232. Wang SV, Rogers JR, Jin Y, Deicicchi D, Dejene S, Connors JM, et al. Stepped-wedge randomised trial to evaluate population health intervention designed to increase appropriate anticoagulation in patients with atrial fibrillation. BMJ Quality and Safety. 2019;28(10):835-42.*
233. Wang Y, Bajorek B. Pilot of a Computerised Antithrombotic Risk Assessment Tool Version 2 (CARATV2.0) for stroke prevention in atrial fibrillation. Cardiol J. 2017;24(2):176-87.
234. Wang Y, Bajorek B. Clinical pre-test of a computerised antithrombotic risk assessment tool for stroke prevention in atrial fibrillation patients: giving consideration to NOACs. Journal of evaluation in clinical practice. 2016;22(6):892-8.*
235. Wang Y, Bajorek B. Selecting antithrombotic therapy for stroke prevention in atrial fibrillation: Health professionals' feedback on a decision support tool. Health informatics journal. 2018;24(3):309-22.*
236. Watzke HH, Forberg E, Svolba G, Jimenez-Boj E, Krinninger B. A prospective controlled trial comparing weekly self-testing and self-dosing with the standard management of patients on stable oral anticoagulation. Thromb Haemost. 2000;83(5):661-5.
237. Weir CJ, Lees KR, Sim I, Erwin L, McAlpine C, Rodger J, et al. Cluster-randomized, controlled trial of computer-based decision support for selecting long-term anti-thrombotic therapy after acute ischaemic stroke. QJM - Monthly Journal of the Association of Physicians. 2003;96(2):143-53.
238. Wijtvliet E, Tieleman RG, van Gelder IC, Pluymaekers N, Rienstra M, Folkeringa RJ, et al. Nurse-led vs. usual-care for atrial fibrillation. Eur Heart J. 2020;41(5):634-41.
239. Willeford A, Leiman V, Noel ZR. Impact of a pharmacist‐to‐dose direct oral anticoagulant protocol on medication errors at an academic medical center. Jaccp: Journal of the American College of Clinical Pharmacy. 2021;4(11):1392-400.
240. Wilson AS, Pham T, Mbusa D, Patel P, Chennupati S, Crawford S, Kapoor A. Pharmacist-led, checklist intervention did not improve adherence in ambulatory patients starting/resuming DOACs. J Am Pharm Assoc (2003). 2023;63(3):878-84 e3.
241. Wilson SJ, Wells PS, Kovacs MJ, Lewis GM, Martin J, Burton E, Anderson DR. Comparing the quality of oral anticoagulant management by anticoagulation clinics and by family physicians: a randomized controlled trial. Cmaj. 2003;169(4):293-8.
242. Yates NY, Hale SA, Clark NP. The Impact of Clinical Pharmacy Services on Direct Oral Anticoagulant Medication Selection and Dosing in the Ambulatory Care Setting. J Pharm Pract. 2024;37(3):671-6.
243. Yermiahu T, Arbelle JE, Shwartz D, Levy Y, Tractinsky N, Porath A. Quality assessment of oral anticoagulant treatment in the Beer-Sheba district. Int J Qual Health Care. 2001;13(3):209-13.
244. Yildirim JG, Bayik Temel A. The Effect of Nurse Home-Support Program on Self-Management of Patients Receiving Oral Anticoagulation (Warfarin) Therapy. Florence Nightingale J Nurs. 2020;28(1):13-22.
245. You JHS, Cheng G, Chan TYK. Comparison of a clinical pharmacist-managed anticoagulation service with routine medical care: Impact on clinical outcomes and health care costs. Hong Kong Medical Journal. 2008;14(3 Supplement 3):S23-S7.

**References to excluded studies (categorised by reason for exclusion):**

**Excludes**

Of the 204 excluded study records:

- 66 studies were excluded due to **absence of implementation:**

1. Alghadeeer S, Alzahrani AA, Alalayet WY, Alkharashi AA, Alarifi MN. Anticoagulation Control of Warfarin in Pharmacist-Led Clinics Versus Physician-Led Clinics: A Prospective Observational Study. Risk Manag Healthc Policy. 2020;13:1175-9.
2. Barrios V, Escobar C, Prieto L, Polo J, Muniz J, Anguita M, GY HL. A new index to predict quality of anticoagulation control in patients on vitamin K antagonists: the DAFNE score. Future Cardiol. 2021;17(4):685-92.
3. Bartoli-Abdou JK, Patel JP, Vadher B, Brown A, Roberts LN, Patel RK, et al. Long-term adherence to direct acting oral anticoagulants and the influence of health beliefs after switching from vitamin-K antagonists: Findings from the Switching Study. Thromb Res. 2021;208:162-9.
4. Bawazeer G, Sales I, Albogami H, Aldemerdash A, Mahmoud M, Aljohani MA, Alhammad A. Crossword puzzle as a learning tool to enhance learning about anticoagulant therapeutics. BMC Med Educ. 2022;22(1):267.
5. Boivin-Proulx LA, Potter BJ, Dorais M, Perreault S. Evolution of and Evidence-Practice Gaps in Antithrombotic Management of Atrial Fibrillation Patients After Percutaneous Coronary Intervention. CJC Open. 2023;5(1):15-23.
6. Bowman S, Weeks P, Chow E, Huang A, Nathan S, Radovancevic R, et al. Implementation of pharmacist‐managed anticoagulation in patients with continuous flow left ventricular assist devices. Jaccp: Journal of the American College of Clinical Pharmacy. 2019;2(6):623-7
7. Brizido C, Ferreira AM, Lopes P, Strong C, Sa Mendes G, Fernandes Gama F, et al. Medication adherence to direct anticoagulants in patients with non-valvular atrial fibrillation - A real world analysis. Rev Port Cardiol (Engl Ed). 2021;40(9):669-75.
8. Bungard TJ, Ritchie B, Garg S, Tsuyuki RT. Sustained impact of anticoagulant control achieved in an anticoagulation management service after transfer of management to the primary care physician. Pharmacotherapy. 2012;32(2):112-9.
9. Cao H, Wu T, Chen W, Fu J, Xia X, Zhang J. The effect of warfarin knowledge on anticoagulation control among patients with heart valve replacement. Int J Clin Pharm. 2020;42(3):861-70.
10. Capiau A, Mehuys E, Van Tongelen I, Christiaens T, De Sutter A, Steurbaut S, et al. Community pharmacy-based study of adherence to non-vitamin K antagonist oral anticoagulants. Heart. 2020;106(22):1740-6.
11. Comuth WJ, de Maat MPM, van de Kerkhof D, Malczynski J, Husted S, Kristensen SD, Munster AB. Adherence to dabigatran etexilate in atrial fibrillation patients intended to undergo electrical cardioversion. Eur Heart J Cardiovasc Pharmacother. 2019;5(2):91-9.
12. Corrochano M, Jimenez B, Millon J, Gich I, Rambla M, Gil E, et al. Patient self-management of oral anticoagulation with vitamin K antagonists in everyday practice: clinical outcomes in a single centre cohort after long-term follow-up. BMC Cardiovasc Disord. 2020;20(1):166.
13. Danchin N, Steg G, Mahe I, Hanon O, Jacoud F, Nolin M, et al. Comparative non-persistence in the first year of treatment with oral anticoagulants in patients with atrial fibrillation: A French comprehensive nationwide study. Arch Cardiovasc Dis. 2022;115(11):571-7.
14. El-Bardissy A, Elewa H, Khalil A, Mekkawi W, Mohammed S, Kassem M, et al. Assessing Pharmacists Knowledge and Attitude Toward the Direct Oral Anticoagulants in Qatar. Clin Appl Thromb Hemost. 2020;26:1076029620933946.
15. Fahmi AM, Mohamed A, Elewa H, Saad MO. Preemptive Dose Adjustment Effect on the Quality of Anticoagulation Management in Warfarin Patients With Drug Interactions: A Retrospective Cohort Study. Clin Appl Thromb Hemost. 2019;25:1076029619872554.
16. Forslund T, Komen JJ, Andersen M, Wettermark B, von Euler M, Mantel-Teeuwisse AK, et al. Improved Stroke Prevention in Atrial Fibrillation After the Introduction of Non-Vitamin K Antagonist Oral Anticoagulants. Stroke. 2018;49(9):2122-8.
17. Franklin JM, Donneyong MM, Desai RJ, Markson L, Girman CJ, McKay C, et al. Variation in adherence to medications across the healthcare system in two comparative effectiveness research cohorts. J Comp Eff Res. 2017;6(7):613-25.
18. Garrison SR, Green L, Kolber MR, Korownyk CS, Olivier NM, Heran BS, et al. The Effect of Warfarin Administration Time on Anticoagulation Stability (INRange): A Pragmatic Randomized Controlled Trial. Ann Fam Med. 2020;18(1):42-9.
19. Gray JA, Lugo RA, Patel VN, Pohland CJ, Stewart DW. First evidence for a pharmacist-led anticoagulant clinic in a medicare part A long term care environment. J Thromb Thrombolysis. 2019;48(4):690-3.
20. Holbrook A, Labiris R, Goldsmith CH, Ota K, Harb S, Sebaldt RJ. Influence of decision aids on patient preferences for anticoagulant therapy: a randomized trial. Cmaj. 2007;176(11):1583-7.
21. Juodvalkis A, West K, Eash T, Foster T, Bentfield B. Assessment of curbside anticoagulation pharmacy visits on clinical outcomes and patient satisfaction. Jaccp: Journal of the American College of Clinical Pharmacy. 2022;5(5):502-8.
22. Kaewsaengeak C, Pienputtarapong U, Tocharoenchok T. The effectiveness of split tablet dosing versus alternate-day dosing of warfarin: a randomized control trial. Sci Rep. 2021;11(1):24060.
23. Keller K, Gobel S, Ten Cate V, Panova-Noeva M, Eggebrecht L, Nagler M, et al. Telemedicine-Based Specialized Care Improves the Outcome of Anticoagulated Individuals with Venous Thromboembolism-Results from the thrombEVAL Study. J Clin Med. 2020;9(10):1-17.
24. Kimmel SE, French B, Kasner SE, Johnson JA, Anderson JL, Gage BF, et al. A pharmacogenetic versus a clinical algorithm for warfarin dosing. N Engl J Med. 2013;369(24):2283-93.
25. Kimmel SE, Troxel AB, French B, Loewenstein G, Doshi JA, Hecht TE, et al. A randomized trial of lottery-based incentives and reminders to improve warfarin adherence: the Warfarin Incentives (WIN2) Trial. Pharmacoepidemiol Drug Saf. 2016;25(11):1219-27.
26. Labovitz AJ, Rose DZ, Fradley MG, Meriwether JN, Renati S, Martin R, et al. Early Apixaban Use Following Stroke in Patients With Atrial Fibrillation: Results of the AREST Trial. Stroke. 2021;52(4):1164-71.
27. Lam ASM, Lee IMH, Mak SKS, Yan BPY, Lee VWY. Warfarin control in Hong Kong clinical practice: a single-centre observational study. Hong Kong Med J. 2020;26(4):294-303.
28. Liu X, Xie G, Li Y, Xiang D, Zhang Y, Chen X, Long J. Evaluation of the algorithm of Anticlot Assistant: an anticoagulant management system based on mobile health technology. Blood Coagul Fibrinolysis. 2021;32(3):221-4.
29. Metaxas C, Albert V, Habegger S, Messerli M, Hersberger KE, Arnet I. Patient Knowledge about Oral Anticoagulation Therapy Assessed during an Intermediate Medication Review in Swiss Community Pharmacies. Pharmacy (Basel). 2020;8(2).
30. Mills C, Snider MJ, Ortman TC, Dush A, Hevezi MS, Li J, et al. Trends in anticoagulation management services following incorporation of direct oral anticoagulants at a large academic medical center. J Thromb Thrombolysis. 2021;51(4):1050-8.
31. Mohamed S, Mei Fong C, Jie Ming Y, Naila Kori A, Abdul Wahab S, Mohd Ali Z. Evaluation of an Initiation Regimen of Warfarin for International Normalized Ratio Target 2.0 to 3.0. J Pharm Technol. 2021;37(6):286-92.
32. Nakajima M, Inatomi Y, Ueda A, Ito Y, Kouzaki Y, Takita T, et al. Preceding direct oral anticoagulant administration reduces the severity of stroke in patients with atrial fibrillation - K-PLUS registry. J Clin Neurosci. 2021;89:106-12.
33. Nigro SC, Boemio N. Using population health‐based strategies to optimize use of direct‐acting oral anticoagulants in atrial fibrillation. Jaccp: Journal of the American College of Clinical Pharmacy. 2022;5(10):1048-54.
34. Noor A, Khan MA, Warsi A, Aseeri M, Ismail S. Evaluation of a pharmacist vs. Haematologist-managed anticoagulation clinic: A retrospective cohort study. Saudi Pharm J. 2021;29(10):1173-80.
35. Noseworthy PA, Branda ME, Kunneman M, Hargraves IG, Sivly AL, Brito JP, et al. Effect of Shared Decision-Making for Stroke Prevention on Treatment Adherence and Safety Outcomes in Patients With Atrial Fibrillation: A Randomized Clinical Trial. J Am Heart Assoc. 2022;11(2):e023048.
36. Oh YH, Hwang SY. Individualized education focusing on self-management improved the knowledge and self-management behaviour of elderly people with atrial fibrillation: A randomized controlled trial. Int J Nurs Pract. 2021;27(4):e12902.
37. Olivia C, Hastie C, Farshid A. Adherence to guidelines regarding anticoagulation and risk factors for progression of atrial fibrillation in a nurse-led clinic. Intern Med J. 2021;51(7):1136-42.
38. Omoush A, Aloush S, M AL, Rayan A, Alkhawaldeh A, Eshah N, et al. Nurses' knowledge of anticoagulation therapy for atrial fibrillation patients: Effectiveness of an educational course. Nurs Forum. 2022;57(5):825-32.
39. Oskarsdottir AR, Gudmundsdottir BR, Jensdottir HM, Flygenring B, Palsson R, Onundarson PT. Ignoring instead of chasing after coagulation factor VII during warfarin management: an interrupted time series study. Blood. 2021;137(20):2745-55.
40. Ozdemir S., Unsal P. Warfarin treatment: home health services verses outpatient clinics. Erciyes Med J. 2021;43(5):438-42.
41. Piersma-Wichers M, van Miert JHA, Veeger N, Kooistra HAM, Meijer K. More precise dosing of acenocoumarol for better control in patients aged above 80 years, a randomised controlled pilot study. Thromb Res. 2020;196:536-8.
42. Poller L, Keown M, Ibrahim S, Lowe G, Moia M, Turpie AG, et al. An international multicenter randomized study of computer-assisted oral anticoagulant dosage vs. medical staff dosage. J Thromb Haemost. 2008;6(6):935-43.
43. Refaai MA, Jacobson AK, Rosenfeld JC, Orr RR. Performance of the microINR Point-of-Care System Used by Self-Testing Patients: A Multicenter Clinical Trial. TH Open. 2021;5(4):e577-e84.
44. Remer HB, Gu X, Haymart B, Barnes GD, Ali MA, Kline-Rogers E, et al. Management strategies following slightly out-of-range INRs: watchful waiting vs dose changes. Blood Adv. 2022;6(10):2977-80.
45. Riva N, Meli S, Borg Xuereb C, Vella K, Ageno W, Makris M, Gatt A. Anticoagulation control with the point-of-care INR: A retrospective pre-/post-analysis. Thromb Res. 2020;196:21-4.
46. Sadiq H, Hoque L, Shi Q, Manning G, Crawford S, McManus D, Kapoor A. SUPPORT-AF III: supporting use of AC through provider prompting about oral anticoagulation therapy for AF. J Thromb Thrombolysis. 2021;52(3):808-16.
47. Sharobeam A, Lin L, Lam C, Garcia-Esperon C, Gawarikar Y, Patel R, et al. Early anticoagulation in patients with stroke and atrial fibrillation is associated with fewer ischaemic lesions at 1 month: the ATTUNE study. Stroke Vasc Neurol. 2024;9(1):30-7.
48. Sherwin E, Schaefer M, Huffmyer M, Naseman K, Davis GA, Schadler A, et al. Evaluation of a pharmacist-led drive-up anticoagulation clinic during the coronavirus 2019 pandemic. Journal of the American Pharmacists Association. 2023;63(1):151-7.e2.
49. Shilbayeh RSA, Abutaily SA, Al Ghwairi LS, Al Madani WO, Almoussa AF, Alzahrani SA. Development and Testing of an Educational Mobile Application for Improving Knowledge among Saudi Patients Receiving Warfarin. International Research Journal of Pharmacy. 2019;10(5):213-21.
50. Rasmussen Skovgaard, Corell P, Madsen P, Overgaard K. Effects of computer-assisted oral anticoagulant therapy. Thrombosis journal. 2012;10(1):17.
51. Sluggett JK, Caughey GE, Air T, Moldovan M, Lang C, Martin G, et al. Medicines use before and after comprehensive medicines review among residents of long-term care facilities: a retrospective cohort study. BMC Geriatr. 2022;22(1):493.
52. Sridharan K, Banny RA, Husain A. Evaluation of Stable Doses of Warfarin in a Patient Cohort. Drug Res (Stuttg). 2020;70(12):570-5.
53. Talana AS, Huber K, Sorin M, Stalvey C, Davis K, Dietrich E. Patient-level adherence and interventions in an interdisciplinary DOAC clinic. Thromb Res. 2019;179:34-6.
54. Tran NT, Lin CH, Do NN, Muradian IK, Lu QD, Henderson SO. The Impact of Implementing an Advance Practice Pharmacist-Led Anticoagulation Clinic Within a Correctional Facility. J Pharm Pract. 2021;34(4):631-4.
55. Turgeon RD, Semchuk WM, Thomson P, Bungard TJ. Exploring discrepancies between pharmacists’ perceived and actual roles towards optimising care in patients prescribed direct oral anticoagulants: a survey. Journal of Pharmacy Practice and Research. 2019;49(4):324-30.
56. Turker M, Sancar M, Demirtunc R, Ucar N, Uzman O, Ay P, et al. Validation of a Knowledge Test in Turkish Patients on Warfarin Therapy at an Ambulatory Anticoagulation Clinic. Turk J Pharm Sci. 2021;18(4):445-51.
57. Van Leeuwen Y, Rombouts EK, Kruithof CJ, Van Der Meer FJM, Rosendaal FR. Improved control of oral anticoagulant dosing: A randomized controlled trial comparing two computer algorithms. Journal of Thrombosis and Haemostasis. 2007;5(8):1644-9.
58. van Miert JHA, Kooistra HAM, Veeger N, Westerterp A, Piersma-Wichers M, Meijer K. Choosing between continuing vitamin K antagonists (VKA) or switching to a direct oral anticoagulant in currently well-controlled patients on VKA for atrial fibrillation: a randomised controlled trial (GAInN). Br J Haematol. 2019;186(3):e21-e3.
59. van Rein N, de Geus KS, Cannegieter SC, Reitsma PH, van der Meer FJM, Lijfering WM. Multi-dose drug dispensing as a tool to improve medication adherence: A study in patients using vitamin K antagonists. Pharmacoepidemiology and Drug Safety. 2018;27(1):46-51.
60. van Zyl M, Wysokinski WE, Jaeger TM, Casanegra AI, Gersh BJ, McBane RD, 2nd. In-home Compared With In-Clinic Warfarin Therapy Monitoring in Mechanical Heart Valves: A Population-Based Study. Mayo Clin Proc Innov Qual Outcomes. 2020;4(5):511-20.
61. Wung JC, Lin HC, Hsu CC, Lin CC, Wang SY, Chang SL, Chang YL. Drug-related problem characterization and the solved status associated factor analysis in a pharmacist-managed anticoagulation clinic. PLoS ONE. 2022;17(8):e0270263.
62. Xian Y, Xu H, O'Brien EC, Shah S, Thomas L, Pencina MJ, et al. Clinical Effectiveness of Direct Oral Anticoagulants vs Warfarin in Older Patients With Atrial Fibrillation and Ischemic Stroke: Findings From the Patient-Centered Research Into Outcomes Stroke Patients Prefer and Effectiveness Research (PROSPER) Study. JAMA Neurol. 2019;76(10):1192-202.
63. Zhang C, Pan MM, Wang N, Wang WW, Li Z, Gu ZC, Lin HW. Feasibility and usability of a mobile health tool on anticoagulation management for patients with atrial fibrillation: a pilot study. Eur J Clin Pharmacol. 2022;78(2):293-304.
64. Zhang L, Li S, Li Z, Yu D, Wu H, Hua B, et al. Social App to Improve Warfarin Therapy in Post-MHVR Chinese Patients: A Randomized Controlled Trial. Cardiovasc Ther. 2023;2023:2342111.
65. Zhu Y, Xu C, Liu J. Randomized controlled trial of genotype-guided warfarin anticoagulation in Chinese elderly patients with nonvalvular atrial fibrillation. J Clin Pharm Ther. 2020;45(6):1466-73.
66. Zhu Z, Li C, Shen J, Wu K, Li Y, Liu K, et al. New Internet-Based Warfarin Anticoagulation Management Approach After Mechanical Heart Valve Replacement: Prospective, Multicenter, Randomized Controlled Trial. J Med Internet Res. 2021;23(8):e29529.

- 25 studies were excluded due to **wrong study design:**

1. Coleman CI, Bunz TJ, Ashton V. Adherence and persistence to rivaroxaban in non-valvular atrial fibrillation patients receiving 30- or 90-day supply prescription fills. Curr Med Res Opin. 2022;38(1):19-26.
2. Dai MF, Li SY, Zhang JF, Wang BY, Zhou L, Yu F, et al. Warfarin anticoagulation management during the COVID-19 pandemic: The role of internet clinic and machine learning. Front Pharmacol. 2022;13:933156.
3. Geng L, Gu J, Li M, Liu H, Sun H, Ni B, et al. Frequency of prothrombin time-international normalized ratio monitoring and the long-term prognosis in patients with mechanical valve replacement. BMC Cardiovasc Disord. 2023;23(1):322.
4. Gomes Freitas C, Walsh M, Coutinho EL, Vincenzo de Paola AA, Atallah AN. Examining therapeutic equivalence between branded and generic warfarin in Brazil: The WARFA crossover randomized controlled trial. PLoS ONE. 2021;16(4):e0248567.
5. Gordon J, Norman M, Hurst M, Mason T, Dickerson C, Sandler B, et al. Using machine learning to predict anticoagulation control in atrial fibrillation: A UK Clinical Practice Research Datalink study. Informatics in Medicine Unlocked. 2021;25.
6. Goto S, Goto S, Pieper KS, Bassand JP, Camm AJ, Fitzmaurice DA, et al. New artificial intelligence prediction model using serial prothrombin time international normalized ratio measurements in atrial fibrillation patients on vitamin K antagonists: GARFIELD-AF. Eur Heart J Cardiovasc Pharmacother. 2020;6(5):301-9.
7. Gray MP, Saba S, Zhang Y, Hernandez I. Outcomes of Patients With Atrial Fibrillation Newly Recommended for Oral Anticoagulation Under the 2014 American Heart Association/American College of Cardiology/Heart Rhythm Society Guideline. J Am Heart Assoc. 2018;7(1):04.
8. Gu ZC, Huang SR, Dong L, Zhou Q, Wang J, Fu B, Chen J. An Adapted Neural-Fuzzy Inference System Model Using Preprocessed Balance Data to Improve the Predictive Accuracy of Warfarin Maintenance Dosing in Patients After Heart Valve Replacement. Cardiovasc Drugs Ther. 2022;36(5):879-89.
9. Hammoudeh A, Khader Y, Tabbalat R, Badaineh Y, Kadri N, Shawer H, et al. One-Year Clinical Outcome in Middle Eastern Patients with Atrial Fibrillation: The Jordan Atrial Fibrillation (JoFib) Study. Int J Vasc Med. 2022;2022:4240999.
10. Hwang AY, Carris NW, Dietrich EA, Gums JG, Smith SM. Evaluation of SAMe-TT(2)R(2) Score on Predicting Success With Extended-Interval Warfarin Monitoring. Ann Pharmacother. 2018;52(11):1085-90.
11. Jonkman LJ, Gwanyanya MP, Kakololo MN, Verbeeck RK, Singu BS. Assessment of anticoagulation management in outpatients attending a warfarin clinic in Windhoek, Namibia. Drugs & Therapy Perspectives. 2019;35(7):341-6.
12. Karlsson LO, Nilsson S, Charitakis E, Bang M, Johansson G, Nilsson L, Janzon M. Clinical decision support for stroke prevention in atrial fibrillation (CDS-AF): Rationale and design of a cluster randomized trial in the primary care setting. American Heart Journal. 2017;187:45-52.
13. Katada Y, Yonezawa A, Utsumi M, Kitada N, Sato YK, Matsumura K, et al. Pharmacist-physician collaborative care for outpatients with left ventricular assist devices using a cloud-based home medical management information-sharing system: a case report. J Pharm Health Care Sci. 2021;7(1):5.
14. Newman TV, Gabriel N, Liang Q, Drake C, El Khoudary SR, Good CB, et al. Comparison of oral anticoagulation use and adherence among Medicare beneficiaries enrolled in stand-alone prescription drug plans vs Medicare Advantage prescription drug plans. J Manag Care Spec Pharm. 2022;28(2):266-74.
15. Salmasi S, Adelakun A, Safari A, Kwan L, MacGillivray J, Andrade JG, et al. Satisfaction With Oral Anticoagulants Among Patients With Atrial Fibrillation: A Prospective Observational Study. CJC Open. 2021;3(11):1347-56.
16. Sayin B, Okutucu S, Yilmaz MB, Ozdemir K, Aydinlar A, Sahin DY, et al. Antithrombotic treatment patterns and stroke prevention in patients with atrial fibrillation in TURKEY: inferences from GARFIELD-AF registry. Anatol J Cardiol. 2019;21(5):272-80.
17. Seagull FJ, Lanham MS, Pomorski M, Callahan M, Jones EK, Barnes GD. Implementing evidence-based anticoagulant prescribing: User-centered design findings and recommendations. Res Pract Thromb Haemost. 2022;6(6):e12803.
18. Selby R, Kaus L, Sealey F, Koo M, Parpia S, Chan B, et al. Quality of anticoagulant control and patient experience associated with long-term warfarin in Canadian patients with non-valvular atrial fibrillation: A multicentre, prospective study. PLoS ONE. 2023;18(4):e0284425.
19. Semakula JR, Mouton JP, Jorgensen A, Hutchinson C, Allie S, Semakula L, et al. A cross-sectional evaluation of five warfarin anticoagulation services in Uganda and South Africa. PLoS ONE. 2020;15(1):e0227458.
20. Shantsila A, Lip GYH, Lane DA. Contemporary management of atrial fibrillation in primary and secondary care in the UK: the prospective long-term AF-GEN-UK Registry. Europace. 2023;25(2):308-17.
21. Tiili P, Leventis I, Kinnunen J, Svedjeback I, Lehto M, Karagkiozi E, et al. Adherence to oral anticoagulation in ischemic stroke patients with atrial fibrillation. Ann Med. 2021;53(1):1613-20.
22. van Miert JHA, Veeger N, Meijer K. An easy-to-use tool to flag patients at risk of poor INR control: A streak of subtherapeutic INRs. Thromb Res. 2019;181:46-51.
23. Verheugt FWA, Ambrosio G, Atar D, Bassand J-P, Camm AJ, Costabel JP, et al. Outcomes in Newly Diagnosed Atrial Fibrillation and History of Acute Coronary Syndromes: Insights from GARFIELD-AF. The American Journal of Medicine. 2019;132(12):1431-40.e7.
24. Vinding NE, Butt JH, Olesen JB, Xian Y, Kristensen SL, Rorth R, et al. Association Between Inappropriately Dosed Anticoagulation Therapy With Stroke Severity and Outcomes in Patients With Atrial Fibrillation. J Am Heart Assoc. 2022;11(6):e024402.
25. Zobeck B, Carson E, MacDowell M, Hunt A, Reeder A. Appointment attendance and patient perception of drive-up INR testing in a rural anticoagulation clinic during the COVID-19 pandemic. J Am Coll Clin Pharm. 2021;4(4):459-64.

- 24 studies were excluded due to **wrong population**:

1. Attia E, Fuentes A, Vassallo M, Dobbs S, Nguyen P, Baker K. Establishing a multidisciplinary taskforce to improve anticoagulation safety at a large health system. Am J Health Syst Pharm. 2022;79(4):297-305.
2. Boriani G, Auricchio A, Botto GL, Joseph JM, Roberts GJ, Grammatico A, et al. Insertable cardiac monitoring results in higher rates of atrial fibrillation diagnosis and oral anticoagulation prescription after ischaemic stroke. Europace. 2023;25(9).
3. Bukovšek AH, Filej B. Empowering patients to self-control and self-management of anticoagulant therapy. Pielegniarstwo XXI wieku / Nursing in the 21st Century. 2023;22(2):79-84.
4. Chen X, Ma X, Cheng Y, Chen S, Shi Y, Ma L. Effect of whole-course nursing intervention on treatment compliance and patient satisfaction in warfarin treatment of patients with lower extremity venous thrombosis. Acta Medica Mediterranea. 2021;37:3197-202.
5. Desteghe L, Engelhard L, Vijgen J, Koopman P, Dilling-Boer D, Schurmans J, et al. Effect of reinforced, targeted in-person education using the Jessa Atrial fibrillation Knowledge Questionnaire in patients with atrial fibrillation: A randomized controlled trial. European Journal of Cardiovascular Nursing. 2019;18(3):194-203.
6. DiRenzo BM, Beam DM, Kline JA, Deodhar KS, Weber ZA, Davis CM, Walroth TA. Implementation and Preliminary Clinical Outcomes of a Pharmacist-managed Venous Thromboembolism Clinic for Patients Treated With Rivaroxaban Post Emergency Department Discharge. Acad Emerg Med. 2018;25(6):634-40.
7. Fraenkel L, Street RL, Jr., Towle V, O'Leary JR, Iannone L, Van Ness PH, Fried TR. A pilot randomized controlled trial of a decision support tool to improve the quality of communication and decision-making in individuals with atrial fibrillation. J Am Geriatr Soc. 2012;60(8):1434-41.
8. Geersing GJ, Hendriksen JMT, Zuithoff NPA, Roes KC, Oudega R, Takada T, et al. Effect of tailoring anticoagulant treatment duration by applying a recurrence risk prediction model in patients with venous thromboembolism compared to usual care: A randomized controlled trial. PLoS Med. 2020;17(6):e1003142.
9. Grant AM, Guthrie B, Dreischulte T. Developing a complex intervention to improve prescribing safety in primary care: Mixed methods feasibility and optimisation pilot study. BMJ Open. 2014;4 (1) (no pagination)(e004153).
10. Gronemann C, Hause S, Assmann A, Neumann J, Schreiber S, Heinze HJ, Goertler M. Modification of In-Hospital Recommendation and Prescription of Anticoagulants for Secondary Prevention of Stroke after Launch of Direct Oral Anticoagulants and Change of National Guidelines. Cerebrovasc Dis. 2020;49(4):412-8.
11. Haile ST, Joelsson-Alm E, Johansson UB, Loof H, Palmer-Kazen U, Gillgren P, Linne A. Effects of a person-centred, nurse-led follow-up programme on adherence to prescribed medication among patients surgically treated for intermittent claudication: randomized clinical trial. Br J Surg. 2022;109(9):846-56.
12. Hald K, Larsen FB, Nielsen KM, Meillier LK, Johansen MB, Larsen ML, et al. Medication adherence, biological and lifestyle risk factors in patients with myocardial infarction: a ten-year follow-up on socially differentiated cardiac rehabilitation. Scand J Prim Health Care. 2019;37(2):182-90.
13. Harding AM, Mitri E, Yeoh M. Improving the safety of anticoagulation initiation in patients discharged from the emergency department. Emerg Med Australas. 2023;35(1):162-4.
14. Javan L, Kazemnejad A, Nomali M, Zakerimoghadam M. Effect of Self-Management Program on Self-efficacy and Medication Adherence in Patients with Mechanical Heart Valve: a Randomized Clinical Trial. J Caring Sci. 2019;8(4):207-11.
15. Kapoor A, Landyn V, Wagner J, Burgwinkle P, Huang W, Gore J, et al. Supplying Pharmacist Home Visit and Anticoagulation Professional Consultation During Transition of Care for Patients With Venous Thromboembolism. J Patient Saf. 2020;16(4):e367-e75.
16. McFadzean IJ, Francis R, Fischetti C, Morton V, Goodfellow S. Direct oral anticoagulant (DOAC) monitoring within primary care: a quality improvement project. BMJ Open Qual. 2023;12(2):06.
17. Menendez-Jandula B, Garcia-Erce JA, Zazo C, Larrad-Mur L. Long-term effectiveness and safety of self-management of oral anticoagulants in real-world settings. BMC Cardiovasc Disord. 2019;19(1):186.
18. Mols RE, Hald M, Vistisen HS, Lomborg K, Maeng M. Nurse-led Motivational Telephone Follow-up After Same-day Percutaneous Coronary Intervention Reduces Readmission and Contacts to General Practice. J Cardiovasc Nurs. 2019;34(3):222-30.
19. Peasah SK, Hammonds T, Liu Y, Campbell V, Manolis C, Good CB. Economic assessment of changes to an existing medication therapy management program of a large regional health plan. J Manag Care Spec Pharm. 2021;27(2):147-56.
20. Perlman A, Horwitz E, Hirsh-Raccah B, Aldouby-Bier G, Fisher Negev T, Hochberg-Klein S, et al. Clinical pharmacist led hospital-wide direct oral anticoagulant stewardship program. Isr J Health Policy Res. 2019;8(1):19.
21. Rudd K, Winans A, Triller D. Standardizing periprocedural anticoagulation management: a stewardship initiative. J Thromb Thrombolysis. 2023;56(3):361-7.
22. Uppuluri EM, McComb MN, Shapiro NL. Implementation of a Direct Oral Anticoagulation Screening Service at a Large Academic Medical Center Provided by a Pharmacist-managed Antithrombosis Clinic as a Method to Expand Antithrombotic Stewardship Efforts. Journal of Pharmacy Practice. 2018.
23. Young J, Nalder MJ, Gorelik A, Elliott RA. Pharmacist-Facilitated Interactive E-Learning for Patients Newly Initiated on Warfarin: A Randomised Controlled Study. Pharmacy (Basel). 2021;10(1).
24. Zado ES, Pammer M, Parham T, Lin D, Frankel DS, Dixit S, Marchlinski FE. "As Needed" nonvitamin K antagonist oral anticoagulants for infrequent atrial fibrillation episodes following atrial fibrillation ablation guided by diligent pulse monitoring: A feasibility study. J Cardiovasc Electrophysiol. 2019;30(5):631-8.

- 19 studies were excluded due to a **combination of reasons:**

1. Claudia LG, Virginia SA, Jesica NR, Alicia CG, Maria Jose NV, Ricardo ZM, et al. Dose adjustment of direct oral anticoagulants dose in elderly institutionalized patients. European Journal of Clinical Pharmacy. 2020;22:178-82.
2. Dietrich F, Polymeris AA, Verbeek M, Engelter ST, Hersberger KE, Schaedelin S, et al. Impact of the COVID-19 lockdown on the adherence of stroke patients to direct oral anticoagulants: a secondary analysis from the MAAESTRO study. J Neurol. 2022;269(1):19-25.
3. Eckman MH, Costea A, Attari M, Munjal J, Wise RE, Knochelmann C, et al. Shared decision-making tool for thromboprophylaxis in atrial fibrillation - A feasibility study. American Heart Journal. 2018;199:13-21.
4. Elliott RA, Woodward MC, Oborne CA. Antithrombotic prescribing in atrial fibrillation: application of a prescribing indicator and multidisciplinary feedback to improve prescribing. Age Ageing. 2002;31(5):391-6.
5. Fatima S, Holbrook A, Schulman S, Park S, Troyan S, Curnew G. Development and validation of a decision aid for choosing among antithrombotic agents for atrial fibrillation. Thromb Res. 2016;145:143-8.
6. Gallagher C, Orchard J, Nyfort-Hansen K, Sanders P, Neubeck L, Hendriks JM. NursE led Atrial Fibrillation Management: The NEAT Study: A Randomized Controlled Trial. J Cardiovasc Nurs. 2020;35(5):456-67.
7. Greger J, Wojcik R, Westphal E, Aladeen T, Landolf K, Boyce S, et al. Pharmacist intervention and anti‐platelet medication monitoring in patients following stroke and transient ischemic attack. Jaccp: Journal of the American College of Clinical Pharmacy. 2020;4(3):311-7.
8. Gurwitz JH, Kapoor A, Garber L, Mazor KM, Wagner J, Cutrona SL, et al. Effect of a Multifaceted Clinical Pharmacist Intervention on Medication Safety After Hospitalization in Persons Prescribed High-risk Medications: A Randomized Clinical Trial. JAMA Intern Med. 2021;181(5):610-8.
9. Kaplan RM, Ziegler PD, Koehler J, Landman S, Sarkar S, Passman RS. Use of Oral Anticoagulation in a Real-World Population With Device Detected Atrial Fibrillation. J Am Heart Assoc. 2020;9(24):e018378.
10. Kovacevic MP, Lupi KE, Wong A, Gilmore JF, Malloy R. Evaluation of the Effect of Apixaban on INR in the Inpatient Population. J Cardiovasc Pharmacol Ther. 2019;24(4):355-8.
11. Kowal D, Katarzynska-Szymanska A, Prech M, Rubis B, Mitkowski P. Early Smartphone App-Based Remote Diagnosis of Silent Atrial Fibrillation and Ventricular Fibrillation in a Patient with Cardiac Resynchronization Therapy Defibrillator. J Cardiovasc Dev Dis. 2023;10(1).
12. McDerby N, Kosari S, Bail K, Shield A, Peterson G, Naunton M. The effect of a residential care pharmacist on medication administration practices in aged care: A controlled trial. J Clin Pharm Ther. 2019;44(4):595-602.
13. Mizobuchi M, Funatsu A, Kobayashi T, Nakamura S. How minimally interrupted direct oral anticoagulants affect intraprocedural anticoagulation during atrial fibrillation ablation? Insights from a Japanese single-center retrospective study. J Arrhythm. 2019;35(5):716-24.
14. Neil WP, Shiokari CE, Burchette RJ, Stapleton D, Ovbiagele B. Mail order pharmacy use and adherence to secondary prevention drugs among stroke patients. J Neurol Sci. 2018;390:117-20.
15. Nguyen A, Gibson S, Wembridge P. Improving medicine information on discharge summaries through implementation of a reconciliation‐based intervention. Journal of Pharmacy Practice and Research. 2022;52(6):454-7.
16. Proietti M, Rivera-Caravaca JM, Esteve-Pastor MA, Marin F, Lip GYH. Stroke and Thromboembolism in Warfarin-Treated Patients with Atrial Fibrillation: Comparing the CHA2DS2-VASc and GARFIELD-AF Risk Scores. Thromb Haemost. 2021;121(8):1107-14.
17. Schneider A, Lorenzoni Nunes P, Uhdich Kleibert KR, Colet CdF, Winkelmann ER. Pharmacotherapeutic follow-up of patients on warfarin in primary care: randomized clinical trial. Revista Colombiana de Ciencias Químico-Farmacéuticas. 2023;51(3):1399-417.
18. Seroussi B, Ouarrirh H, Elalamy I, Gerotziafas G, Debrix I, Bouaud J. Development and Assessment of RecosDoc-MTeV to Improve the Quality of Direct Oral Anticoagulant Prescription for Venous Thromboembolic Disease. Stud Health Technol Inform. 2019;264:793-7.
19. Showande SJ, Orok EN. Impact of pharmacists' training on oral anticoagulant counseling: A randomized controlled trial. Patient Educ Couns. 2021;104(5):1253-9.

- 17 studies were excluded due to **no relevant outcomes:**

1. Alhmoud E, Abdelsamad O, Soaly E, Enany RE, Elewa H. Anticoagulation clinic drive-up service during COVID-19 pandemic in Qatar. J Thromb Thrombolysis. 2021;51(2):297-300.
2. Arbel A, Abu-Ful Z, Preis M, Cohen S, Saliba W. Implementation of oral anticoagulation treatment guidelines in patients with newly diagnosed atrial fibrillation. Br J Clin Pharmacol. 2021;87(12):4747-55.
3. Austin JA, Barras MA, Woods LS, Sullivan CM. The Effect of Digitization on the Safe Management of Anticoagulants. Appl Clin Inform. 2022;13(4):845-56.
4. Bahit MC, Granger CB, Alexander JH, Mulder H, Wojdyla DM, Hanna M, et al. Regional variation in clinical characteristics and outcomes in patients with atrial fibrillation: Findings from the ARISTOTLE trial. Int J Cardiol. 2020;302:53-8.
5. Blythin K, Harries E. Medicines optimisation in East Sussex care homes. Prescriber. 2019;30(5):18-23.
6. Cox JL, Parkash R, Foster GA, Xie F, MacKillop JH, Ciaccia A, et al. Integrated Management Program Advancing Community Treatment of Atrial Fibrillation (IMPACT-AF): A cluster randomized trial of a computerized clinical decision support tool. Am Heart J. 2020;224:35-46.
7. Gardiner C, Williams K, Mackie IJ, Machin SJ, Cohen H. Can oral anticoagulation be managed using telemedicine and patient self-testing? A pilot study. Clin Lab Haematol. 2006;28(2):122-5.
8. Kish K, Lekic S. Implementation of warfarin to direct oral anticoagulant conversion initiative in pharmacist-run anticoagulation clinics during COVID-19 pandemic. J Am Coll Clin Pharm. 2021;4(9):1154-60.
9. Rezaei Bookani K, Minga I, Chander M, Hankewych K, Plassmeier M, Tafur A. Drive-Through Model for Anticoagulation Clinics During the COVID-19 Pandemic. Clin Appl Thromb Hemost. 2020;26:1076029620947476.
10. Spencer-Bonilla G, Thota A, Organick P, Ponce OJ, Kunneman M, Giblon R, et al. Normalization of a conversation tool to promote shared decision making about anticoagulation in patients with atrial fibrillation within a practical randomized trial of its effectiveness: a cross-sectional study. Trials. 2020;21(1):395.
11. Thomas H, Smyth L. Implementation and Evaluation of a Standardized Non-Vitamin K Oral Anticoagulant (NOAC) Patient Safety Alert Card Across the Northern Region of England. J Prim Care Community Health. 2020;11:2150132719894758.
12. Thompson JL, Sundt TM, Sarano ME, Santrach PJ, Schaff HV. In-Patient International Normalized Ratio Self-Testing Instruction After Mechanical Heart Valve Implantation. Ann Thorac Surg. 2008;85(6):2046-50.
13. Voller H, Dovifat C, Glatz J, Kortke H, Taborski U, Wegscheider K. Self management of oral anticoagulation with the IN Ratio system: impact of a structured teaching program on patient's knowledge of medical background and procedures. Eur J Cardiovasc Prev Rehabil. 2004;11(5):442-7.
14. Woo BFY, Lim TW, Tam WWS. The Translation of Knowledge Into Practice in the Management of Atrial Fibrillation in Singapore. Heart Lung Circ. 2019;28(4):605-14.
15. Yang Q, Quan X, Zhang Y, Feng G, Zhang T, Wang C, et al. An exploratory study of effectiveness and safety of rivaroxaban in patients with left ventricular thrombus (R-DISSOLVE). J Thromb Thrombolysis. 2023;55(4):649-59.
16. Zhao J, Wang H, Li X, Hu Y, Yan VKC, Wong CKH, et al. Importance of attributes and willingness to pay for oral anticoagulant therapy in patients with atrial fibrillation in China: A discrete choice experiment. PLoS Med. 2021;18(8):e1003730.
17. Zhu Z, Li C, Shen J, Wu K, Li Y, Liu K, et al. New Internet-Based Warfarin Anticoagulation Management Approach After Mechanical Heart Valve Replacement: Prospective, Multicenter, Randomized Controlled Trial. J Med Internet Res. 2021;23(8):e29529.

- 16 studies were excluded due to **duplicate record:**

1. Ashburner JM, Atlas SJ, Khurshid S, Weng LC, Hulme OL, Chang Y, et al. Electronic physician notifications to improve guideline-based anticoagulation in atrial fibrillation: a randomized controlled trial. J Gen Intern Med. 2018;33(12):2070-7.
2. Barnes GD, Kong X, Cole D, Haymart B, Kline-Rogers E, Almany S, et al. Extended International Normalized Ratio testing intervals for warfarin-treated patients. J Thromb Haemost. 2018;16(7):1307-12.
3. Bowman S, Weeks P, Chow E, Huang A, Nathan S, Radovancevic R, et al. Implementation of pharmacist-managed anticoagulation in patients with continuous flow left ventricular assist devices. JACCP Journal of the American College of Clinical Pharmacy. 2019.
4. Brasen CL, Madsen JS, Parkner T, Brandslund I. Home Management of Warfarin Treatment Through a Real-Time Supervised Telemedicine Solution: A Randomized Controlled Trial. Telemed J E Health. 2019;25(2):109-15.
5. Desteghe L, Vijgen J, Koopman P, Dilling-Boer D, Schurmans J, Dendale P, Heidbuchel H. Telemonitoring-based feedback improves adherence to non-vitamin K antagonist oral anticoagulants intake in patients with atrial fibrillation. Eur Heart J. 2018;39(16):1394-403.
6. Desteghe L, Engelhard L, Vijgen J, Koopman P, Dilling-Boer D, Schurmans J, et al. Effect of reinforced, targeted in-person education using the Jessa Atrial fibrillation Knowledge Questionnaire in patients with atrial fibrillation: A randomized controlled trial. European Journal of Cardiovascular Nursing. 2019;18(3):194-203.
7. Loewen PS, Bansback N, Hicklin J, Andrade JG, Kapanen AI, Kwan L, et al. Evaluating the Effect of a Patient Decision Aid for Atrial Fibrillation Stroke Prevention Therapy. Ann Pharmacother. 2019;53(7):665-74.
8. Marquez-Contreras E, Martell-Claros N, Marquez-Rivero S, Hermida-Campa E, Gracia-Diez C, Sanchez-Lopez E, et al. Strategies for improving dabigatran adherence for stroke prevention in patients with non-valvular atrial fibrillation: education and drug intake reminders (FACILITA study). Curr Med Res Opin. 2018;34(7):1301-8.
9. Mertens C, Siebenhofer A, Berghold A, Pregartner G, Ulrich LR, Mergenthal K, et al. Differences in the quality of oral anticoagulation therapy with vitamin K antagonists in German GP practices - results of the cluster-randomized PICANT trial (Primary Care Management for Optimized Antithrombotic Treatment). BMC Health Serv Res. 2019;19(1):539.
10. Mifsud EM, Wirth F, Camilleri L, Azzopardi LM, Serracino-Inglott A. Pharmacist-led medicine use review in community pharmacy for patients on warfarin. Int J Clin Pharm. 2019;41(3):741-50.
11. Refaai MA, Shah V, Fernando R. Performance of the microINR Point-of-Care System: A Multicenter Clinical Trial. Thromb Haemost. 2020;120(4):687-91.
12. Siebenhofer A, Ulrich LR, Mergenthal K, Berghold A, Pregartner G, Kemperdick B, et al. Primary care management for patients receiving long-term antithrombotic treatment: A cluster-randomized controlled trial. PLoS ONE. 2019;14(1):e0209366.
13. Taborski U, Voller H, Kortke H, Blunt J, Wegscheider K. Self Management of Oral Anticoagulation with the INRatio System: Accuracy and Reliability Following a Two-Day Structured Training Program. Laboratory Medicine. 2004;35(5):303-7.
14. Uppuluri EM, McComb MN, Shapiro NL. Implementation of a Direct Oral Anticoagulation Screening Service at a Large Academic Medical Center Provided by a Pharmacist-managed Antithrombosis Clinic as a Method to Expand Antithrombotic Stewardship Efforts. Journal of Pharmacy Practice. 2018.
15. van Rein N, de Geus KS, Cannegieter SC, Reitsma PH, van der Meer FJM, Lijfering WM. Multi-dose drug dispensing as a tool to improve medication adherence: A study in patients using vitamin K antagonists. Pharmacoepidemiology and Drug Safety. 2018;27(1):46-51.
16. Wang SV, Rogers JR, Jin Y, DeiCicchi D, Dejene S, Connors JM, et al. Stepped-wedge randomised trial to evaluate population health intervention designed to increase appropriate anticoagulation in patients with atrial fibrillation. BMJ Qual Saf. 2019;28(10):835-42.

- 14 studies were excluded due to **wrong setting (in patients only):**

1. Ahuja T, Raco V, Papadopoulos J, Green D. Antithrombotic Stewardship: Assessing Use of Computerized Clinical Decision Support Tools to Enhance Safe Prescribing of Direct Oral Anticoagulants in Hospitalized Patients. J Patient Saf. 2021;17(8):e1057-e61.
2. Brown A, Cavell G, Dogra N, Whittlesea C. The impact of an electronic alert to reduce the risk of co-prescription of low molecular weight heparins and direct oral anticoagulants. Int J Med Inform. 2022;164:104780.
3. Cook DA, Enders F, Caraballo PJ, Nishimura RA, Lloyd FJ. An automated clinical alert system for newly-diagnosed atrial fibrillation. PLoS ONE. 2015;10(4):e0122153.
4. Dreijer AR, Diepstraten J, Leebeek FWG, Kruip M, van den Bemt P. The effect of hospital-based antithrombotic stewardship on adherence to anticoagulant guidelines. Int J Clin Pharm. 2019;41(3):691-9.
5. Fontaine A, Mahe I, Bergmann JF, Fiessinger JN, Dhote R, Cohen P, Vinceneux P. Effectiveness of written guidelines on the appropriateness of thromboprophylaxis prescriptions for medical patients: a prospective randomized study. J Intern Med. 2006;260(4):369-76.
6. Harris JR, Hatch R, Vallabhajosyula P, Lo Y, Mowery D, Patel N. AM Versus PM Postoperative Administration of Warfarin With a Mechanical Mitral Valve. J Pharm Technol. 2021;37(2):89-94.
7. Kim JJ, Mohammad RA, Coley KC, Donihi AC. Use of an iPad to Provide Warfarin Video Education to Hospitalized Patients. J Patient Saf. 2015;11(3):160-5.
8. Kuhrau S, Masic D, Mancl E, Brailovsky Y, Porcaro K, Morris S, et al. Impact of Pulmonary Embolism Response Team on Anticoagulation Prescribing Patterns in Patients With Acute Pulmonary Embolism. J Pharm Pract. 2022;35(1):38-43.
9. Lachuer C, Benzengli H, Do B, Rwabihama JP, Leglise P. Oral anticoagulants: Interventional pharmaceutical study with reminder of good practices, and iatrogenic impact. Ann Pharm Fr. 2021;79(4):409-17.
10. McVannel T, Tangedal K, Haines A, Semchuk WM. Anticoagulation Interventions by Pharmacists in Acute Care. Can J Hosp Pharm. 2023;76(2):126-30.
11. Miele C, Taylor M, Shah A. Assessment of direct oral anticoagulant prescribing and monitoring pre- and post-implementation of a pharmacy protocol at a community teaching hospital. Hospital Pharmacy. 2017;52(3):207-13.
12. Quintens C, Verhamme P, Vanassche T, Vandenbriele C, Van den Bosch B, Peetermans WE, et al. Improving appropriate use of anticoagulants in hospitalised patients: A pharmacist-led Check of Medication Appropriateness intervention. Br J Clin Pharmacol. 2022;88(6):2959-68.
13. Sun MC, Hsiao PJ. In-hospital case management to increase anticoagulation therapy for stroke patients with atrial fibrillation: A hospital-based registry. Journal of the Formosan Medical Association. 2013;112(5):263-8.
14. To E, Jackevicius C. Quality improvement initiative for Pharmacist-Assisted Warfarin Dosing: Implementation and evaluation of a new protocol. Canadian Journal of Hospital Pharmacy. 2002;55(2):105-13.

- 6 studies were excluded due to **wrong drug:**

1. Li A, Del Olmo MG, Fong M, Sim K, Lymer SJ, Cunich M, Caterson I. Effect of a smartphone application (Perx) on medication adherence and clinical outcomes: a 12-month randomised controlled trial. BMJ Open. 2021;11(8):e047041.
2. Li PWC, Yu DSF, Yan BP. Nurse-led multi-component behavioural activation programme to improve health outcomes in patients with atrial fibrillation: a mixed-methods study and feasibility analysis. Eur J Cardiovasc Nurs. 2023;22(6):655-63.
3. Matsumoto Y, Fukushima S, Shimahara Y, Kawamoto N, Tadokoro N, Kuroda K, et al. Early postoperative heparinization reduce hemolysis in patients with HeartMate II devices. J Artif Organs. 2020;23(1):19-26.
4. Schurr JW, Stevens CA, Bane A, Culbreth SE, Miller AL, Connors JM, Sylvester KW. Evaluation of Compliance with a Weight-based Nurse-driven Heparin Nomogram in a Tertiary Academic Medical Center. Crit Pathw Cardiol. 2018;17(2):83-7.
5. Venechuk GE, Khazanie P, Page RL, 2nd, Knoepke CE, Helmkamp LJ, Peterson PN, et al. An Electronically delivered, Patient-activation tool for Intensification of medications for Chronic Heart Failure with reduced ejection fraction: Rationale and design of the EPIC-HF trial. Am Heart J. 2020;229:144-55.
6. Wessol JL. Feasibility and Acceptability of a SystemCHANGE(™) Inter vention to Improve Medication Adherence in Older Adult Stroke Survivors: A Pilot Randomized Controlled Trial. Feasibility & Acceptability of a SystemCHANGE(TM) Intervention to Improve Medication Adherence in Older Adult Stroke Survivors: A Pilot Randomized Controlled Trial. 2018:1-.

- 3 studies were **protocols:**

1. Chen L, Zhou YZ, Zhou XM, Liu LM, Xu P, Zhang X, Tan SL. Evaluation of the "safe multidisciplinary app-assisted remote patient-self-testing (SMART) model" for warfarin home management during the COVID-19 pandemic: study protocol of a multi-center randomized controlled trial. BMC Health Serv Res. 2021;21(1):875.
2. Costa JMD, Marcolino MS, Torres HC, Resende RE, Souza RP, Barbosa HC, et al. Protocol of a clinical trial study involving educational intervention in patients treated with warfarin. Medicine (Baltimore). 2019;98(22):e15829.
3. Jones AE, McCarty MM, Brito JP, Noseworthy PA, Cavanaugh KL, Cameron KA, et al. Randomized evaluation of decision support interventions for atrial fibrillation: Rationale and design of the RED-AF study. Am Heart J. 2022;248:42-52.

- 3 studies were **wrong outcomes:**

1. Neshewat J, Wasserman A, Alexandris-Souphis C, Haymart B, Feldeisen D, Kong X, et al. Reduction in epistaxis and emergency department visits in patients taking warfarin after implementation of an education program. Thromb Res. 2021;199:119-22.
2. Reinhardt SW, Desai NR, Tang Y, Jones PG, Ader J, Spertus JA. Personalizing the decision of dabigatran versus warfarin in atrial fibrillation: A secondary analysis of the Randomized Evaluation of Long-term anticoagulation therapY (RE-LY) trial. PLoS ONE. 2021;16(8):e0256338.
3. Woo BFY, Tam WWS, Rangpa T, Liau WF, Nathania J, Lim TW. A Nurse-Led Integrated Chronic Care E-Enhanced Atrial Fibrillation (NICE-AF) Clinic in the Community: A Preliminary Evaluation. Int J Environ Res Public Health. 2022;19(8).

- 3 studies contained **no disaggregated data:**

1. Najafi H, Rakhshan M. Effect of self-management interventions on complications of atrial fibrillation: A clinical trial. Biomedical Research (India). 2018;29(12):2484-9.
2. Xu N, Wang C, Wan J, Liu X, Li Z, Chen M. Effectiveness of pharmacist intervention in the management of coronary artery disease after index percutaneous coronary intervention: A single center randomized controlled trial. International Journal of Clinical and Experimental Medicine. 2019;12:7760-5.
3. Zhang ZX, Schroeder-Tanka J, Stooker W, Wissen S, Khorsand N. Management of combined oral antithrombotic therapy by an antithrombotic stewardship program: A prospective study. Br J Clin Pharmacol. 2022;88(9):4092-9.

- 3 studies were **beyond review scope**

1. Piccini JP, Xu H, Cox M, Matsouaka RA, Fonarow GC, Butler J, et al. Adherence to Guideline-Directed Stroke Prevention Therapy for Atrial Fibrillation Is Achievable. Circulation. 2019;139(12):1497-506.
2. Wan LH, Zhang XP, You LM, Ruan HF, Chen SX. The Efficacy of a Comprehensive Reminder System to Improve Health Behaviors and Blood Pressure Control in Hypertensive Ischemic Stroke Patients: A Randomized Controlled Trial. J Cardiovasc Nurs. 2018;33(6):509-17.
3. Spyropoulos AC, Giannis D, Cohen J, John S, Myrka A, Inlall D, et al. Implementation of the Management of Anticoagulation in the Periprocedural Period App Into an Electronic Health Record: A Prospective Cohort Study. Clin Appl Thromb Hemost. 2020;26:1076029620925910.

- 2 studies excluded due to **wrong timeframe:**

1. Sawicki PT, Glaser B, Kleespies C, Stubbe J, Schmitz N, Kaiser T, Didjurgeit U. Long-Term Results of Patients' Self-Management of Oral Anticoagulation. Journal of Clinical and Basic Cardiology. 2003;6(1-4):59-62.
2. White RH, McCurdy SA, von Marensdorff H, Woodruff DE, Jr., Leftgoff L. Home prothrombin time monitoring after the initiation of warfarin therapy. A randomized, prospective study. Ann Intern Med. 1989;111(9):730-7.

- 2 studies were **abstract only:**

1. Goudie BM, Danskin KL, Al-Agilly SS, Fairfield G, Cunningham AD, McGrego JE, Cachia PG. Near Patient Monitoring of Anticoagulant Therapy in General Practice. Scott Med J. 2016;49(2):70-.
2. Viswanathan K, Beith C, Pittaway L, Veevers W, Vickers C. An integrated multi-professional approach to AF management: Initial experience and short-term outcomes of a new rapid access clinic in secondary care. Europace. 2018;20 (Supplement 4):iv54.

- 1 study was a **secondary analysis**:

1. Christensen TD, Maegaard M, Sorensen HT, Hjortdal VE, Hasenkam JM. Self- versus conventional management of oral anticoagulant therapy: effects on INR variability and coumarin dose in a randomized controlled trial. Am J Cardiovasc Drugs. 2007;7(3):191-7.

**Appendix 5:** Examples of limited and detailed descriptions of implementation strategies.

- *Salient content in italics.*

**Limited:**

*Each patient was provided with* a CoaguChek XS monitor (Roche Diagnostics NZ Ltd). The testing devices and test‐strips were *provided free* by Roche Diagnostic for the trial period. The *patients were* *taught how to perform an INR test* using a finger prick blood sample. The patients used an *online decision support package* (INR Online Ltd, Palmerston North, NZ) to assist with dose recommendations. Each patient had *access to a secure website* protected by username and password. *Access was free* for the trial period. (Harper, 2011)

**Detailed:**

*The pharmacists were trained* in the use of the CoaguChek S INR monitor and *given educational material* relating to warfarin. The training typically involved *approximately 2–3h* with the pharmacists *discussing anticoagulation and the use of the INR monitor*. Pharmacists were *shown how to conduct INR tests* and were also *observed conducting tests* on consenting subjects or pharmacy staff. Problems or difficulties encountered by the researchers [through previous research activities (17, 18) and personal experience] were raised with the pharmacists and *potential solutions to these difficulties* were discussed. The pharmacists were *provided with a laminated colour brochure* on the INR monitor, and *ongoing assistance* if needed. *Pharmacists were provided with* INR monitors, tests strips and other consumables *free of charge* for the duration of the trial.

*Local GPs were visited, informed of the availability* of the CoaguChek S monitor in their region, and were invited to refer their patients to the pharmacy for POC testing. During the visits to the GPs, *the accuracy of the CoaguChek S monitor* (17, 18) and its use in several overseas countries *was discussed*.

*Patients* referred to the pharmacy or who were identified as taking warfarin *were given an information sheet* and gave written informed consent to undergo fingerprick testing at the pharmacy. Patients could have two types of testing performed in the pharmacy: comparison testing was defined as a pharmacy‐based test taken within 4 h of conventional laboratory testing, and additional testing was a pharmacy‐based test with no direct comparison laboratory test taken. All results were sent to the patient's GP via a specially designed fax form. The results of the testing, such as INR, time taken, outcome of test (dosage changes) were recorded. *It was recommended to pharmacists* that all results were recorded for patients in the standard warfarin educational booklet.

*Pharmacists and GPs were instructed* that this type of testing was not to replace conventional pathology testing. The service was *offered free of charge to patients* for the duration of the trial. *Pharmacies were remunerated at a rate of $4 per test* for the duration of the trial. (Jackson, 2005)

**Appendix 6:** Table 4: Factors associated with the use of implementation strategies based on ERIC Clusters

|  | Engage consumers | | Use evaluative & iterative strategies | | Change infrastructure | | Adapt & tailor to the context | | Develop stakeholder interrelationships | | Utilise financial strategies | | Support clinicians | | | Provide interactive assistance | | Train & educate stakeholders | |  |  |
| --- | --- | --- | --- | --- | --- | --- | --- | --- | --- | --- | --- | --- | --- | --- | --- | --- | --- | --- | --- | --- | --- |
|  | N  (%) | aOR  (95% CI) | N  (%) | aOR  (95% CI) | N  (%) | aOR  (95% CI) | N  (%) | aOR  (95% CI) | N  (%) | aOR  (95% CI) | N  (%) | aOR  (95% CI) | N% | aOR  (95% CI) | | N  (%) | aOR  (95% CI) | N  (%) | aOR  (95% CI) |  |  |
| **Study outcomes** | | | | | | | | | | | | | | | | | | | | | |
| Favourable | 52  (33.3) | 1.2  (.5-2.7) | 56  (35.9) | 1.9  (.8-4.7) | 46  (29.5) | 1.4  (.6-3.5) | 37  (23.7) | 1.9  (.7-4.9) | 46  (29.5) | 1.3  (.6-3.0) | 31  (19.9) | .5  (.2-1.1) | 60  (38.5) | 1.5  (.5-2.5) | | 50  (32.1) | **3.9**  **(1.5-9.7)*** | 108  (69.2) | 1.1  (.5-2.6) |  |  |
| **Identifies as an implementation study** | | | | | | | | | | | | | | | | | | | | | |
| Yes | 14  (23) | .7  (.3-1.6) | 30  (49.2) | 1.7  (.8-4) | 26  (42.6) | 1.8  (.8-4) | 22  (36.1) | **2.6 (1.2-6.1)*** | 24  (39.3) | 1.4  (.7-3) | 18  (29.5) | 1.0  (.4-2.3) | 28  (45.9) | 1.3  (.7-2.7) | | 17  (27.9) | .9  (.4-1.9) | 32  (52.5) | **.4**  **(.2-.8)*** |  |  |
| **Year of publication** | | | | | | | | | | | | | | | | | | | | | |
| 2000-2004 | 10  (45.5) | ref | 3  (13.6) | ref | 3  (13.6) | ref | 3  (13.6) | ref | 1  (4.5) | ref | 1  (4.5) | ref | 6  (27.3) | ref | | 3  (13.6) | ref | 19  (86.4) | ref |  |  |
| 2005-2009 | 11  (37.9) | 1.0  (.3-3.5) | 5  (17.2) | 2.5  (.4-14.2) | 5  (17.2) | 1.2  (.2-6.5) | 5  (17.2) | 1.2  (.2-6.4) | 5  (17.2) | 4.3  (.4-42.9) | 2  (6.9) | 1.5  (.1- 19.4) | 8  (27.6) | .8  (.2-3.0) | | 3  (10.3) | .9  (.2-5.1) | 22  (75.9) | .4  (.1-2.2) |  |  |
| 2010-2014 | 12  (29.3) | .77  (.2-2.7) | 10  (24.4) | 3.6  (.7-18.9) | 8  (19.5) | 1.9  (.4-10) | 3  (7.3) | .40  (.1-2.5) | 11  (26.8) | 5.91  (.7-53.3) | 4  (9.8) | 1.3  (.1-13.9) | 11  (26.8) | .8  (.2-2.8) | | 13  (31.7) | **4.5**  **(1.0-19.9)*** | 29  (70.7) | .4  (.1-1.7) |  |  |
| 2015-2019 | 12  (20.7) | .5  (.1-1.9) | 14  (24.1) | 1.8  (.3-10.1) | 15  (25.9) | 2.0  (.4-10.3) | 11  (19) | .8  (.2-4.1) | 20  (34.5) | 7.3  (.8-65.8) | 13  (22.4) | 3.0  (.3-29.3) | 26  (44.8) | 1.3  (.4-4.6) | | 20  (34.5) | **6.0**  **(1.3-27.6)*** | 36  (62.1) | .3  (.1-1.6) |  |  |
| 2020-2023 | 25  (37.9) | 1.2  (.3-4.6) | 39  (59.1) | **11.1**  **(2.0-61.1)*** | 26  (39.4) | 3.4  (.7-17.6) | 24  (36.4) | 1.6  (.3-8.0) | 22  (33.3) | 6.6  (.7-60.8) | 28  (42.4) | 8.9  (.9-84.2) | 26  (39.4) | .9  (.3-3.4) | | 21  (31.8) | **5.7**  **(1.2-27.2)*** | 41  (62.1) | .5  (.1-2.8) |  |  |
| **Study Design** | | | | | | | | | | | | | | | | | | | | | |
| Observational | 13  (27.7) | ref | 23  (48.9) | ref | 18  (38.3) | ref | 17  (36.2) | ref | 17  (36.2) | ref | 14  (29.8) | Ref | 22  (46.8) | | ref | 13  (27.7) | ref | 25  (53.2) | ref |  |  |
| Quasi Experimental | 22  (27.2) | .7  (.3-2.0) | 29  (35.8) | 1.2  (.4-3.2) | 23  (28.4) | .90  (.3-2.3) | 15 (18.5) | **.34**  **(.1-1.0)*** | 28  (34.6) | .88  (.4-2.2) | 21 (25.9) | 1.1  (.4- 3.1) | 33  (40.7) | | .7  (.3-1.7) | 25  (30.9) | 1.0  (.4-2.6) | 60  (74.1) | 2.0  (.8-5.1) |  |  |
| RCT or variant-RCT | 35  (39.8) | 1.2  (.4-3.2) | 19  (21.6) | .7  (.2-2.1) | 16  (18.2) | .7  (.3-2.0) | 14  (15.9) | .5  (.2-1.5) | 14  (15.9) | .4  (.2-1.2) | 13  (14.8) | .4  (.1-1.2) | 22  (25) | | **.4**  **(.2-1.0)*** | 22  (25) | 1.2  (.5-3.4) | 62  (70.5) | 1.1  (.4-2.9) |  |  |
| **Type of OAC** | | | | | | | | | | | | | | | | | | | | | |
| VKAs only (Warfarin) | 47  (33.6) | ref | 35  (25) | ref | 33  (23.6) | ref | 24  (17.1) | ref | 31  (22.1) | ref | 23  (16.4) | ref | 45  (32.1) | ref | | 37  (26.4) | ref | 103  (73.6) | ref |  |  |
| VKAs and DOACS | 14  (35) | 1.6  (.5-4.5) | 25  (62.5) | **9.2**  **(2.8-30.4)**** | 14  (35) | 1.1  (.4-3.3) | 11  (27.5) | 1.5  (.5-4.6) | 15  (37.5) | 1.2  (.4-3.3) | 15  (37.5) | 1.6  (.5-4.6) | 21  (52.5) | 1.6  (.6-4.1) | | 13  (32.5) | 1.0  (.4-2.7) | 25  (62.5) | .6  (.2-1.8) |  |  |
| DOACs only | 6  (26) | .9  (.2-3.3) | 10  (43.5) | 2.1  (.5-7.8) | 7  (30.4) | .6  (.2-2.0) | 9  (39.1) | 1.6  (.5-5.3) | 10  (43.5) | 2.7  (.9-8.4) | 8  (34.8) | 1.3  (.4-4.3) | 6  (26.1) | .5  (.2-1.7) | | 6  (26.1) | 1.0  (.3-3.6) | 11  (47.8) | .5  (.2-1.7) |  |  |
| **Population** | | | | | | | | | | | | | | | | | | | | | |
| AF + other indication | 35  (33.7) | ref | 35  (33.7) | ref | 32  (30.8) | ref | 23  (22.1) | ref | 24  (23.1) | ref | 19  (18.3) | Ref | 39  (37.5) | ref | | 30  (28.8) | ref | 72  (69.2) | ref |  |  |
| AF only | 18  (29) | .91  (.4-2.2) | 15  (24.2) | **.3**  **(.1-1.0)*** | 13  (21) | .5  (.1-1.2) | 13  (21) | 1.2  (.4-3.0) | 19  (30.6) | 1.5  (.6-3.8) | 13  (21) | 1.5  (.5-4.2) | 22  (35.5) | .8  (.3-1.7) | | 17  (27.4) | 1.1  (.5-2.8) | 43  (69.4) | 1.8  (.7-4.5) |  |  |
| NVAF | 4  (40) | .8  (.1-4.0) | 5  (50) | .5  (.1-3.4) | 4  (40) | 1.3  (.2-6.7) | 3  (30) | 1.0  (.2-5.0) | 4  (40) | 1.7  (.4-8.4) | 6  (60) | 3.9  (.8-19.7) | 5  (50) | 2.3  (.5-12.0) | | 3  (30) | .6  (.1-3.2) | 5  (50) | .4  (.1-1.8) |  |  |
| Other indications | 6  (33.3) | .9  (.3-3.2) | 7  (38.9) | 2.8  (.8-10.7) | 2  (11.1) | .3  (.0-1.5) | 1  (5.6) | .2  (.0-2.3) | 5  (27.8) | 2.0  (.5-7.2) | 4  (22.2) | 3.0  (.7-13.2) | 3  (16.7) | .3  (.1-1.4) | | 5  (27.8) | 1.0  (.3-3.7) | 13  (72.2) | .8  (.2-3.2) |  |  |
| **Setting** | | | | | | | | | | | | | | | | | | | | | |
| Primary and secondary care | 12  (40) | ref | 15 (50) | ref | 8  (26.7) | ref | 5  (16.7) | ref | 7  (23.3) | ref | 5  (16.7) | ref | 11  (36.7) | Ref | | 5  (16.7) | Ref | 15  (50) | ref |  |  |
| Secondary care | 45  (33.6) | 1.4  (.4-4.5) | 38 (28.4) | 2.7  (.8 – 8.8) | 30  (22.4) | .9 (.3-2.8) | 29  (21.6) | .6  (.1-1.8) | 34  (25.4) | .6 (.2-2.0) | 26  (19.4) | .3 (.1-1.1) | 43  (31.1) | .8 (.3-2.2) | | 39  (29.1) | .5 (.1-1.6) | 98  (73.1) | **.3 (.1-.9)*** |  |  |
| Primary care | 13  (25) | .8  (.3-2.0) | 18  (34.6) | .5  (.2 – 1.4) | 19  (36.5) | .8 (.3-1.8) | 12  (23.1) | 1.11 (.4-2.9) | 18  (34.6) | .7 (.3-1.5) | 17  (32.7) | **.3  (.1-.7)*** | 23  (44.1) | .8 (.4-1.8) | | 16  (30.8) | .8 (.3-1.8) | 34  (65.4) | .9 (.4-2.0) |  |  |
| **Number of interventions tested** | | | | | | | | | | | | | | | | | | | | | |
| *M* (SD)  Range [1-6] |  | 1.4  (1.0-2.1) |  | 1.1  (.7-1.7) |  | 1.0  (.7-1.6) |  | 1.1  (.7-1.7) |  | **1.51**  **(1.0-2.2)*** |  | 1.30  (.9-2.0) |  | 1.2  (.9-1.8) | |  | 1.3  (.3-2.0) |  | 1.4  (.9-2.2) | |  |
| **Intervention type** | | | | | | | | | | | | | | | | | | | | | |
| Service Reorganisation | 9  (20) | ref | 12  (26.7) | ref | 14  (31.1) | ref | 6  (13.3) | ref | 18  (40) | ref | 9  (20) | ref | 21  (46.7) | ref | | 13  (28.9) | ref | 32  (71.1) | ref | |  |
| Provider-focused | 9  (13.2) | .5  (.2-1.6) | 29  (42.6) | 2.2  (.7-6.4) | 25  (36.8) | 1.3  (.5-3.4) | 21  (30.9) | 2.4  (.7-7.7) | 18  (26.5) | .4  (.1-1.0)* | 16  (23.5) | .90  (.3-2.9) | 27  (39.7) | .7  (.3-1.8) | | 11  (16.2) | .4  (.1-1.1) | 33  (48.5) | .4  (.2-1.1) | |  |
| Patient-focused | 43  (48.9) | **3.7**  **(1.4-9.7)*** | 23  (26.1) | 1.3  (.5-4.0) | 10 (11.4) | .3  (.1-.9)* | 14  (15.9) | 1.5  (.4-5.2) | 19  (21.6) | .4  (.2-1.2) | 19  (21.6) | 1.1  (.3-3.9) | 23  (26.1) | .5  (.2-1.1) | | 31  (35.2) | 1.6  (.6-4.2) | 70  (79.5) | 1.3  (.5-3.5) | |  |
| Multi-category | 9  (60) | 3.9  (.9-16.4) | 7  (46.7) | 4.8  (.8-30.2) | 8  (53.3) | 3.4  (.8-14.8) | 5  (33.3) | 2.3  (.4-12.1) | 4  (26.7) | .3  (.1-1.6) | 4  (26.7) | .9  (.2-5.2) | 6  (40) | .8  (.2-3.2) | | 5  (33.3) | .9  (.2-4.3) | 12  (80) | 1.2  (.2-6.4) | |  |

Note; ***** p≤0.05 ******p≤0.001. Key: aOR: adjusted Odds Ratio; CI: Confidence Interval; DOACs: Direct Oral Anticoagulants; M: Mean; NVAF: Non-Valvular Atrial Fibrillation; OR: Odds Ratio; SD: Standard Deviation; RCT: Randomised Controlled Trial; VKAs: Vitamin K Antagonists
