# Supplementary material for: Optimisation of oral anticoagulation for stroke prevention: a scoping review of factors influencing implementation
Source: BMJ Open. 2025 Dec 29;15(12):e097847. doi: 10.1136/bmjopen-2024-097847 (PMC12750798; doi:10.1136/bmjopen-2024-097847)
Supplement: Supplementary file 2 [file bmjopen-2024-097847.reviewer_comments.pdf]

## PEER REVIEW HISTORY

BMJ Open publishes all reviews undertaken for accepted manuscripts. Reviewers are asked to complete a checklist review form and are provided with free text boxes to elaborate on their assessment. These free text comments are reproduced below.

### ARTICLE DETAILS

**Title (Provisional)**

Optimisation of oral anticoagulation for stroke prevention: a scoping review of factors influencing implementation

**Authors**

Weldon, Jo Catherine; Bray, Emma P; Gibson, Josephine; Bangee, Munirah; Chesworth, Brigit; Doherty, Alison; Hirst, Yasemin; Lane, Deirdre; Harris, Cath; Patel, Aasima Saeed; Watkins, Caroline

---

### VERSION 1 - REVIEW

---

| | |
|---|---|
| **Reviewer** | **1** |
| **Name** | **Serpa, Frans** |
| **Affiliation** | **The University of Texas Southwestern Medical Center, Internal Medicine** |
| **Date** | **01-Jan-2025** |
| **COI** | **None** |

---

The increasing use of oral anticoagulants makes identifying effective implementation strategies a highly relevant topic. This study provides a comprehensive review of the existing literature and clearly identifies gaps in how implementation studies are reported. By applying the ERIC implementation strategy clusters, the authors outline and describe the key features of the current body of research. The findings may encourage readers and stakeholders to explore the relationships between specific strategy clusters and outcomes in future studies.

---

| | |
|---|---|
| **Reviewer** | **2** |
| **Name** | **Levine, Steven** |
| **Affiliation** | **SUNY Downstate Medical Center College of Medicine, Neurology** |
| **Date** | **17-Sep-2025** |
| **COI** | **None** |

---

This is a comprehensive scoping review of an important clinical topic with practical implications for patient benefit. It is well written and the methodology is clear and appropriate.

---

## VERSION 1 - AUTHOR RESPONSE

Thank you for considering our manuscript and for the reviewer comments.
